# Damage recognition by intestinal stem cells via Draper promotes adult *Drosophila* midgut regeneration

**DOI:** 10.1038/s44318-026-00890-1

**Published:** 2026-08-05

**Authors:** Martina Legido, Marie Srotyr, Rebecca Wafer, Parthive H Patel

**Affiliations:** School of Biochemistry and Cellular and Molecular Medicine, Faculty of Health and Life Sciences, University of Bristol, University Walk, Bristol, UK

**Keywords:** Immunology, Signal Transduction, Stem Cells & Regenerative Medicine

## Abstract

Tissue regeneration after injury is crucial for restoring epithelial structure and function. Upon damage, a regenerative microenvironment forms that provides signalling cues that stimulate stem cell proliferation to replace lost cells. While this process is well understood, how stem cells themselves sense damage, translate this input into their proliferation and shape the regenerative microenvironment remains unclear. Here we show that Draper-Src-Shark signalling in *Drosophila* intestinal stem cells (ISCs) recognises tissue damage by sensing externalised phosphatidylserine on dying midgut epithelial cells and is required for STAT activation in ISCs to promote their proliferation. Unlike its role in phagocytosis, Draper in ISCs does not promote the clearance of these apoptotic enterocytes but rather facilitates ISC proliferation in the presence of damaged enterocytes. Moreover, Draper-Src-Shark signalling in progenitors regulates STAT transcriptional activity in the adjacent visceral muscle, indicating that progenitors can shape the regenerative microenvironment beyond tissue boundaries. As Src and STAT are also activated in the mammalian intestinal epithelium after damage and tumour formation, these findings may help develop therapies for tissue regeneration, inflammatory diseases and cancer.

## Synopsis

Somatic stem cell proliferation after damage is thought to rely mainly on proliferative cues from the regenerative microenvironment. This study shows that adult *Drosophila* intestinal stem cells (ISCs) directly sense damage to facilitate their own proliferation and shape local tissue responses.

- Phosphatidylserine externalisation by apoptotic midgut enterocytes after damage triggers STAT-induced ISC proliferation.
- Draper-Src-Shark signalling stimulates STAT activity in ISCs, promoting their proliferation following midgut damage.
- Unlike its role in phagocytosis, Draper signalling in ISCs does not promote apoptotic enterocyte clearance.
- Draper signalling in progenitors non-autonomously promotes STAT activation in the visceral muscle after midgut damage, thus shaping the regenerative microenvironment.

## Abstract (web summary)

Somatic stem cells in the gut directly sense damage to facilitate proliferation and tissue recovery.

## Graphical abstract

**Figure:**
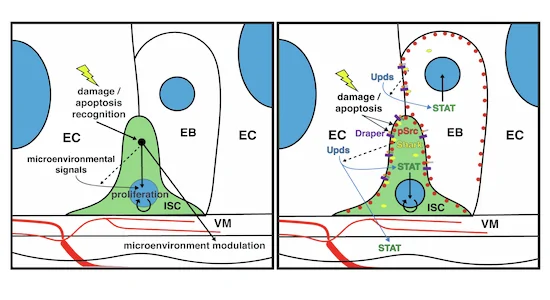

## Introduction

A failure to properly maintain or regenerate an epithelium results in the loss of its integrity, architecture and function, and thus also organismal homeostasis (Poss and Tanaka, 2024; Turner, 2009). In many adult epithelia, stem cells divide to replace damaged or lost tissue cell types (Blanpain et al, 2007; Pellettieri and Sanchez Alvarado, 2007). Stem cells are coaxed to proliferate by signalling cues provided by a regenerative microenvironment that forms shortly after injury (Hageman et al, 2020; Lane et al, 2014; Palikuqi et al, 2022). This microenvironment can include other epithelial cells as well as other cell types and tissues associated with the organ. While much is known about how signalling cues provided by the regenerative microenvironment promote stem cell proliferation in several adult epithelia, we know less about how stem cells sense damage and how they modify their behaviour, such as their proliferation, to support regeneration. Furthermore, even less is understood about how stem cells modulate their microenvironment after tissue damage to support regeneration.

**Figure 1:**
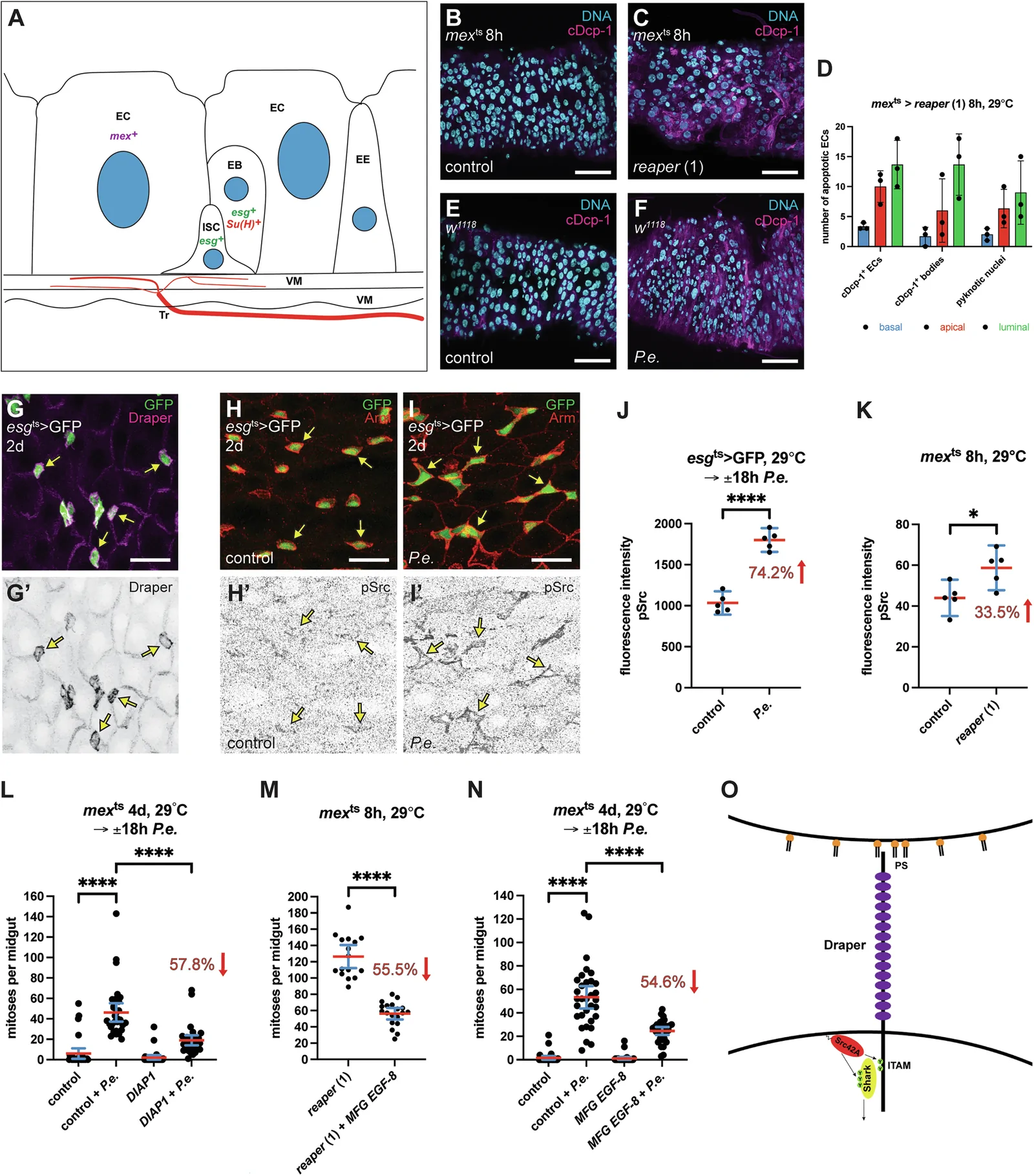
Phosphatidylserine externalisation after damage-induced apoptosis triggers ISC proliferation in the adult *Drosophila* midgut. The adult *Drosophila* midgut epithelium (**A**) is maintained by intestinal stem cells (ISCs, escargot-positive, *esg*^*+*^). ISCs divide to produce enteroblasts (EBs, *esg*^+^ and *Suppressor of Hairless*-positive (*Su(H)*^+^)) that differentiate into enterocytes (ECs, *malic enzyme modifier*-positive (*mex*^+^)). ISCs also produce *prospero*-positive enteroendocrine cells (EEs). The midgut epithelium is enveloped by the visceral muscle (VM) and trachea (Tr). Apoptotic ECs are eliminated into the lumen (**B**–**D**). Cleaved Death Caspase-1 (cDcp-1)-positive apoptotic ECs and bodies (magenta) increased in midguts expressing *reaper* in ECs (**C**) compared to control midguts (**B**). cDcp-1^+^ ECs, cDcp-1^+^ apoptotic bodies and EC pyknotic nuclei are found on the basal and apical sides of the epithelium and in the lumen. The mean number of apoptotic ECs progressively increased from the basal side of the epithelium to the lumen (**D**). Pathogenic bacterial infection increases cDcp1^+^ apoptotic epithelial cells (**E**, **F**). cDcp-1^+^ midgut epithelial cells increased after *P.e*. infection (**F**) compared to uninfected control midguts (**E**). High levels of Draper (magenta) are found in progenitors (esg>GFP^+^, green; yellow arrows) (**G**–**G’**). Activated Src accumulates cortically in progenitors after midgut damage (**H**–**J**). Phosphorylated Src Y419 (pSrc) levels increased in progenitors (*esg* > GFP^+^, green; Arm^high^, red; yellow arrows) of *P.e*.-infected midguts (**I**, **I’**) compared to those in uninfected control midguts (**H**, **H’**). The mean cortical pSrc levels increased in progenitors of *P.e*.-infected midguts (*n* = 5 midguts from one of three experiments) compared to uninfected control midguts (*n* = 5 midguts from one of three experiments) (unpaired *t* test; *P* < 0.0001) (**J**). EC apoptosis increases cortical pSrc accumulation in progenitors (**K**). The mean cortical pSrc levels increased in progenitors of midguts expressing *reaper* in ECs (*n* = 5 midguts from one of two experiments) compared to control midguts (*n* = 5 midguts from one of two experiments) (unpaired *t* test; *P* = 0.0201). Blocking EC apoptosis inhibits ISC proliferation after midgut damage (**L**). The mean mitoses per midgut increased in *P.e*.-infected control midguts (*n* = 33 midguts from two experiments) compared to uninfected control midguts (*n* = 31 midguts from two experiments) (Mann–Whitney; *P* < 0.0001). The mean mitoses per midgut decreased in *P.e*.-infected midguts expressing DIAP1 in ECs (*n* = 33 midguts from two experiments) compared to *P.e*.-infected control midguts (Mann–Whitney; *P* < 0.0001). Phosphatidylserine externalisation triggers ISC proliferation after EC apoptosis and midgut damage (**M**, **N**). The mitoses per midgut decreased in midguts expressing *reaper* and *milk fat globule EGF-8* (*MFG EGF-8*) in ECs (*n* = 19 midguts from one of two experiments) compared to midguts expressing *reaper* alone in ECs (*n* = 16 midguts from one of two experiments) (Mann–Whitney; *P* < 0.0001) (**M**). The mean mitoses per midgut increased in *P.e*.-infected control midguts (*n* = 32 midguts from two experiments) compared to uninfected control midguts (*n* = 31 midguts from two experiments) (Mann–Whitney; *P* < 0.0001). The mean mitoses per midgut decreased in *P.e*.-infected midguts expressing *MFG EGF-8* in ECs (*n* = 32 midguts from two experiments) compared to *P.e*.-infected control midguts (Mann–Whitney; *P* < 0.0001) (**N**). Phosphatidylserine-Draper-Src42A-Shark signalling (**O**). Externalised phosphatidylserine (PS) on an apoptotic cell binds to Draper. Phosphorylation of tyrosines (Y) in the immunoreceptor tyrosine-based activation motif (ITAM) of Draper by Src42A recruits Shark, leading to Shark activation and signalling. Mean (red line) and 95% confidence interval (blue bars) are shown. Scale bars in (**B**, **C**, **E**, **F**) are 50 μm and in (**G**, **H**, **I**) are 20 μm. Significance levels are indicated by *P ≤ 0.05, **P ≤ 0.01, ***P ≤ 0.001, ****P ≤ 0.0001, and ns, for not significant (*P* > 0.05). Source data are available online for this figure.

The mammalian intestinal epithelium is maintained by intestinal stem cells (ISCs) that give rise to differentiated absorptive and secretory epithelial cells (Beumer and Clevers, 2021). Upon damage to the mammalian intestine, several signalling cues are received by ISCs that stimulate their proliferation, including epidermal growth factor (EGF), cytokines and Wnts (Hageman et al, 2020; Palikuqi et al, 2022). These cues are provided by cells within the regenerative microenvironment, which include resident epithelial cells, immune cells, mesenchyme, vasculature and enteric glial cells (Hageman et al, 2020; Palikuqi et al, 2022). Similar to the mammalian intestine, the adult *Drosophila* midgut epithelium (Fig. 1A) is maintained by ISCs that produce two main epithelial cell types, absorptive enterocytes (ECs) and secretory enteroendocrine cells (EEs) (Micchelli and Perrimon, 2006; Miguel-Aliaga et al, 2018; Ohlstein and Spradling, 2006). ISCs divide to produce an enteroblast (EB) about 90–95% of the time (Biteau and Jasper, 2014; Miguel-Aliaga et al, 2018; Ohlstein and Spradling, 2007). The EB receives a high Notch signal from the ISC and differentiates into an EC (Beehler-Evans and Micchelli, 2015; Biteau and Jasper, 2014). On the other hand, EEs arise from ISCs via an enteroendocrine precursor (EEp) and by direct ISC differentiation into an EE (Zeng and Hou, 2015). Together, ISCs, EBs and EEps are considered adult midgut progenitors and express the progenitor marker, *escargot* (*esg*) (Micchelli and Perrimon, 2006). The fly midgut epithelium is surrounded by two layers of visceral muscle (VM) and oxygen-carrying trachea (Miguel-Aliaga et al, 2018; Perochon et al, 2021; Tamamouna et al, 2021). Together, the EBs, ECs, EEs, the VM and trachea contribute to the regenerative microenvironment for ISCs after midgut damage by producing signalling cues that directly stimulate ISC proliferation. These cues include the unpaired cytokines Upd1-3, EGFs, Wnt, Hedgehog, fibroblast growth factor and PTK7/Off-track (Biteau and Jasper, 2011; Cordero et al, 2012; Hu et al, 2021; Jiang et al, 2011; Jiang et al, 2009; Osman et al, 2012; Perochon et al, 2021; Tamamouna et al, 2021; Tian et al, 2015; Zhou et al, 2013).

Many of the signals received by fly ISCs are also received by mammalian ISCs from the regenerative microenvironment after damage. One such microenvironment-provided cue promoting regeneration of both the *Drosophila* and mammalian intestines is cytokine-STAT signalling. STAT (Stat92E) activity in *Drosophila* ISCs is required for their proliferation after damage caused by pathogenic bacteria (Buchon et al, 2009a; Jiang et al, 2009). STAT activation within progenitors is through cytokine-like Unpaireds (Upd1-3) produced by enteroblasts and damaged enterocytes upon pathogenic bacterial infection (Jiang et al, 2009; Osman et al, 2012; Tian et al, 2015; Zhou et al, 2013). Furthermore, cytokine expression is thought to be largely induced by damage-sensing pathways (JNK, p38, Hippo) in ECs (Herrera and Bach, 2019; Patel et al, 2019; Zhang and Edgar, 2022; Zhou and Boutros, 2023). In the mammalian intestine, STAT3 is activated after epithelial damage by either γ-irradiation or infection by the mouse pathogen, *Citrobacter* *rodentium* (Cordero et al, 2014; Wittkopf et al, 2015) and is required for epithelial regeneration after intestinal damage (Oshima et al, 2019; Pickert et al, 2009). STAT3 is thought to be partly activated by cytokines produced by macrophages and type 3 innate lymphoid cells (Hageman et al, 2020; Palikuqi et al, 2022).

Whilst much is known about the signals received by ISCs from the regenerative microenvironment upon damage, some recent evidence from both invertebrates and vertebrates has shown that epithelial damage sensing directly by stem cells themselves is essential for stem cell-mediated regeneration (Guo et al, 2024; Liu et al, 2023). In the wounded mouse skin, epidermal stem cells at the wound margin detect hypoxia caused by local blood vessel damage and respond by producing the cytokine, IL-24. This cytokine activates STAT3 in epidermal stem cells, stimulating their proliferation (Liu et al, 2023). Similarly, in the adult *Drosophila* and mouse intestines, ISCs sense an increase in very long-chain fatty acids within the regenerative microenvironment after injury. This is mediated by peroxisome proliferator-activated receptors (PPARs), which promote differentiation by increasing peroxisome numbers and Sox21 activity (Guo et al, 2024). These studies strongly indicate that stem cells can directly sense tissue damage and activate intrinsic mechanisms that promote regeneration without signalling cues from the regenerative microenvironment.

Damaged cells may undergo apoptosis or necrosis after tissue injury. Apoptosis is observed in the midgut epithelium after damage caused by pathogenic bacterial infection and detergents (Buchon et al, 2009b; Loudhaief et al, 2017; Petsakou et al, 2023). EC apoptosis in the adult *Drosophila* midgut is known to trigger ISC proliferation by stimulating cytokine (Upd1-3) production in the midgut, which stimulates STAT activity in ISCs (Jiang et al, 2009). An early event of apoptosis and necrosis is the externalisation of phosphatidylserine, a phospholipid normally found on the intracellular side of the plasma membrane (Nagata et al, 2016). It is therefore possible that phosphatidylserine and other damage-associated molecular patterns (DAMPs) from dying cells (Ma et al, 2024) may be recognised by midgut ISCs, possibly altering their behaviour, but to date this has remained unexplored.

It therefore remains unclear how, mechanistically, ISCs sense intestinal damage and translate this into their proliferation to regenerate the intestinal epithelium. The non-receptor tyrosine kinase Src is a crucial intrinsic factor required by both adult *Drosophila* and mammalian ISCs in response to tissue damage to induce ISC proliferation. In adult *Drosophila*, Src42A signalling is required in progenitors for ISC proliferation after pathogenic bacterial infection (Cordero et al, 2014; Kohlmaier et al, 2015). The *Drosophila* genome encodes two Src family kinases, Src42A and Src64B. Whereas Src64B expression can stimulate ISC proliferation in unchallenged midguts, its requirement for ISC proliferation after midgut damage is not clear (Cordero et al, 2014; Kohlmaier et al, 2015). Src is activated in progenitors by Wnt/Wingless (Wg) and receptor tyrosine kinase Ret signalling after midgut damage (Cordero et al, 2014) (Perea et al, 2017). Constitutive activation of Src42A in progenitors strongly induces ISC proliferation, which requires STAT (Cordero et al, 2014; Kohlmaier et al, 2015). Similarly, in the mouse intestine, both Src and STAT3 are activated after γ-irradiation, and STAT3 activation after intestinal damage partly required Src (Cordero et al, 2014). However, it is not entirely understood in both the mouse intestine and the adult *Drosophila* midgut how Src is activated after damage and how Src activates STAT3 and Stat92E, respectively.

In *Drosophila*, Src42A signalling can activate STAT via Shark signalling. To do so, Src family kinases phosphorylate tyrosines within immunoreceptor tyrosine-based activation motifs (ITAMs) on immune receptors, such as Draper (Mocsai et al, 2010; Williamson and Vale, 2018; Ziegenfuss et al, 2008). Upon ligand binding, Draper proteins microcluster on the plasma membrane. These microclusters exclude bulky transmembrane phosphatases, enabling Draper to remain phosphorylated by Src42A. Src42A-mediated phosphorylation of tyrosine residues within Draper ITAMs then allows the non-receptor tyrosine kinase, Spleen tyrosine kinase (Syk)—Shark in *Drosophila*—to dock onto ITAMs in Draper via their Src-homology 2 (SH2) domains (Williamson and Vale, 2018; Ziegenfuss et al, 2008). This allows for full SYK activation by enabling phosphorylation of the linker region between its SH2 and kinase domains by Src family kinases (Mansueto et al, 2019; Mocsai et al, 2010) (Fig. 1O). In *Drosophila*, non-professional phagocytes, such as glia and epithelial cells (Etchegaray et al, 2012; Han et al, 2014; Kurant et al, 2008; MacDonald et al, 2006), use Draper to detect and clear apoptotic corpses or debris (Melcarne et al, 2019). Indeed, Draper-Src-Shark signalling is used by glial cells to clear severed axons from neurons after injury (MacDonald et al, 2006; Melcarne et al, 2019; Ziegenfuss et al, 2008). Furthermore, glia activate STAT through Draper-Src-Shark signalling after adult *Drosophila* olfactory receptor neuron (ORN) axonal injury (Doherty et al, 2014). Draper recognises dying cells through binding of exposed phosphatidylserine on the outside surface of apoptotic cells, which promotes Draper phosphorylation and phagocytosis (Ji et al, 2023; Tung et al, 2013; Williamson and Vale, 2018).

Thus, Draper-Src-Shark signalling might be utilised by ISCs to detect apoptotic cells within damaged midguts and couple this to ISC proliferation, but such a role of Draper-Src-Shark-STAT signalling has not yet been explored. While the mammalian Draper homologue, MEGF10, is required in muscle satellite cells for skeletal muscle regeneration (Li et al, 2021), very little is known about the role of MEGF10 or SYK in stem cells during regeneration.

Here, we explore how adult *Drosophila* midgut ISCs sense damage after midgut injury and translate this into a regenerative response. We find that Draper-Src-Shark signalling is required within ISCs for adult *Drosophila* midgut regeneration by recognising midgut damage and translating it to ISC proliferation. Mechanistically, Draper-Src-Shark signalling recognises externalised phosphatidylserine on dying ECs and stimulates ISC proliferation by facilitating STAT signalling in ISCs. Furthermore, we show that Draper-Src-Shark signalling in progenitors activates STAT in the visceral muscle, demonstrating how progenitors shape the regenerative microenvironment to support epithelial regeneration.

## Results

### Phosphatidylserine externalisation by enterocytes after midgut damage triggers ISC proliferation

Enterocyte apoptosis in the midgut triggers ISC proliferation via epithelial cytokine Upd1-3 production that activates JAK-STAT signalling in ISCs (Jiang et al, 2009). Expression of the pro-apoptotic factor, *reaper*, in ECs strongly increases the number of cleaved Dcp-1-positive (cDcp1^+^) apoptotic cells in the midgut epithelium compared to control (Fig. 1B,C). Specifically, we observed cDcp-1^+^ ECs, cDcp-1^+^ apoptotic bodies and pyknotic nuclei in the midgut epithelium after EC ablation via *reaper* expression. Furthermore, we found apoptotic ECs basally in the midgut epithelium near the progenitors, apically in the epithelium closer to the lumen and in the lumen (Fig. 1D). We determined the number of cDcp-1^+^ ECs, cDcp-1^+^ apoptotic bodies and pyknotic nuclei in each of these locations. Whilst all three classes of apoptotic ECs were found in these locations, the number of each class increased progressively from the basal epithelium towards the lumen (Fig. 1D). These data indicate that apoptotic ECs are primarily eliminated into the lumen.

Oral infection of adult flies with pathogenic bacteria induces apoptosis in the midgut epithelium (Buchon et al, 2010; Buchon et al, 2009b; Loudhaief et al, 2017; Zhai et al, 2018). Indeed, we similarly found an increase in cDcp-1^+^ apoptotic cells in the midgut epithelium after *Pseudomonas entomophila* (*P.e*.) infection (Fig. 1E,F). An early event of apoptosis is the externalisation of phosphatidylserine, a phospholipid found normally on the inner side of the plasma membrane (Nagata et al, 2016). The immunoreceptor Draper has been shown to recognise phosphatidylserine (Melcarne et al, 2019; Tung et al, 2013); thus, we determined whether midgut epithelial cells expressed Draper. We found that *esg*^*+*^ progenitors express high levels of Draper on their plasma membrane, suggesting they could sense exposed phosphatidylserine on apoptotic ECs (Fig. 1G,G’). Draper was found on the plasma membrane of progenitors facing towards other midgut epithelial cells but not the visceral muscle. We also found a lower expression of Draper on the lateral membrane of ECs (Fig. 1G,G’). These data suggest that midgut progenitors may be able to sense exposed phosphatidylserine through Draper after midgut damage.

**Figure EV1:**
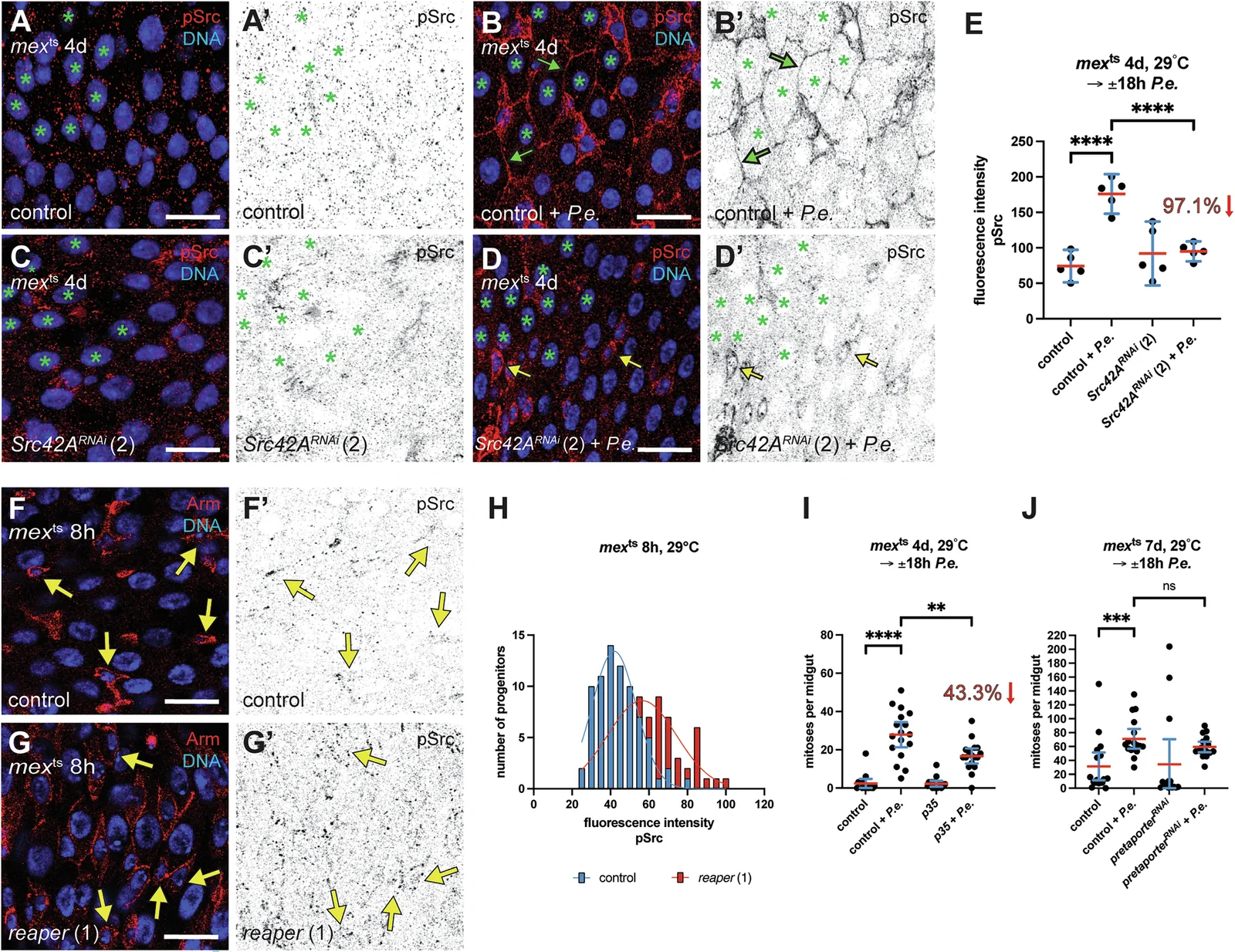
Midgut damage and enterocyte apoptosis activate Src in progenitors. Anti-phosphorylated human Src Y419 (pSrc) antibody detects Src42A activation (**A**–**E**). Levels of pSrc (**A**, **B**; red) increased on the lateral membranes (green arrows) of enterocytes (ECs, green asterisks) and cortically within progenitors after *P.e*. infection (**B**–**B’**) compared to uninfected midguts (**A**–**A’**). ECs expressing *Src42A*^*RNAi*^ had decreased pSrc in their lateral membranes after *P.e*. infection compared to control *P.e*.-infected midguts (**D**–**D’**, **B**–**B’**). Note in (**D**–**D’**), progenitors (yellow arrows) still cortically accumulated pSrc after *P.e*. infection (**D**–**D’**). Mean pSrc levels increased in the epithelium of *P.e*.-infected midguts (*n* = 5 midguts from one experiment) compared to uninfected control midguts (*n* = 5 midguts from one experiment) (unpaired *t* test; *P* < 0.0001). Mean cortical pSrc levels decreased in the epithelium of *P.e*.-infected midguts expressing *Src42A*^*RNAi*^ in ECs (*n* = 5 midguts from one experiment) compared to *P.e*.-infected control midguts (unpaired *t* test; *P* < 0.0001) (**E**). Apoptosis increases cortical pSrc accumulation in progenitors (**F**–**H**). Levels of pSrc (**F’**, **G’**) increased cortically within progenitors (yellow arrows) after *reaper* expression in ECs (**G’**) compared to control midguts (**F’**). Mean cortical pSrc levels increased in progenitors of midguts expressing *reaper* in ECs (*n* = 75 cells from five midguts from one of two experiments) (**H**, red) compared to control midguts (*n* = 75 cells from five midguts from one of two experiments) (**H**, blue). Blocking apoptosis in ECs inhibits ISC proliferation after *P.e*. infection (**I**). The mean mitoses per midgut increased in *P.e*.-infected control midguts (*n* = 17 midguts from one experiment) compared to uninfected control midguts (*n* = 16 midguts from one experiment) (Mann–Whitney; *P* < 0.0001). The mean mitoses per midgut decreased in *P.e*.-infected midguts expressing p35 in ECs (*n* = 17 midguts from one experiment) compared to *P.e*.-infected control midguts (Mann–Whitney; *P* = 0.0052). Pretaporter is not required in dying ECs for ISC proliferation after *P.e*. infection (**J**). The mean mitoses per midgut increased in *P.e*.-infected control midguts (*n* = 17 midguts from one experiment) compared to uninfected control midguts (*n* = 17 midguts from one experiment) (Mann–Whitney; *P* = 0.0003). The mean mitoses per midgut did not significantly decrease in *P.e*.-infected midguts expressing *pretaporter*^*RNAi*^ in ECs (*n* = 17 midguts from one experiment) compared to *P.e*.-infected control midguts (Mann–Whitney; *P* = 0.1619). Mean (red line) and 95% confidence intervals (blue bars) are shown. Scales bars in (**A**, **B**, **C**, **D**, **F**, **G**) are 20 μm. Significance levels are indicated by *P ≤ 0.05, **P ≤ 0.01, ***P ≤ 0.001, ****P ≤ 0.0001, and ns, for not significant (*P* > 0.05). Source data are available online for this figure.

The cytoplasmic portion of Draper contains an immunoreceptor tyrosine-based activation motif (ITAM) with tyrosines known to be phosphorylated by Src42A (Williamson and Vale, 2018; Ziegenfuss et al, 2008). Thus, we next determined whether Src42A is activated in progenitors after *P.e*. infection. Oral infection of adult flies with gram-negative pathogenic bacteria (*P.e*. or *Erwinia caratovora caratovora 15*, *Ecc15*) has been shown to increase the levels of activated Src in the midgut epithelium, including within *esg*^*+*^ progenitors (Cordero et al, 2014; Houtz et al, 2017). However, the subcellular localisation of activated Src was not clear. Using an antibody recognising activated Src (phosphorylated tyrosine 419 on human Src, pSrc) (Cordero et al, 2014; Perea et al, 2017), we found that Src is activated after *P.e*. infection in *esg*^+^ progenitors and in ECs (Figs. 1H–I’ and EV1A–D’). Moreover, we found a strong cortical increase by 74.2% of activated Src in *esg*^*+*^ progenitors (Fig. 1J), which partially overlapped with Armadillo (Arm). High levels of Arm are found at the cortex of adult midgut progenitors (Fig. 1H,I) (Chen et al, 2018; Maeda et al, 2008), and progenitors were marked by expressing GFP with the progenitor-specific driver *esg-GAL4 tubGAL80*^ts^ (*esg*^ts^) (Micchelli and Perrimon, 2006). We validated that the pSrc antibody is specific for activated Src42A: whereas depletion of Src42A with RNAi in ECs with the EC-specific driver, *mex-GAL4 tubGAL80*^ts^ (*mex*^ts^) (Phillips and Thomas, 2006), decreased pSrc levels in ECs after *P.e*. infection, activated Src42A levels still increased in progenitors (Fig. EV1A–E). These data suggest that activated Src42A accumulates in the cortex of progenitors after pathogenic bacterial infection. Interestingly, we observed an increase in pSrc levels (33.5%) in progenitors after EC ablation by expressing *reaper* with *mex*^ts^ (Figs. 1K and EV1F–H). These data suggest that EC apoptosis can increase pSrc levels within progenitors. Since ISC proliferation is strongly induced after EC ablation (Fig. 1M), these data further suggest that ISC proliferation after damage requires only mild increases in pSrc.

EC apoptosis triggers ISC proliferation to promote midgut epithelial regeneration (Jiang et al, 2009). Inhibition of apoptosis by the expression of p35 in ECs can partially inhibit ISC proliferation that occurs after DNA damage (Amcheslavsky et al, 2009). Thus, we tested whether blocking apoptosis in ECs inhibits ISC proliferation after *P.e*. infection by expressing the apoptosis inhibitors, DIAP1 and p35, with *mex*^ts^. We found that DIAP and p35 expression in ECs indeed partially blocked ISC proliferation by 57.8% and 43.3%, respectively, in *P.e*.-infected midguts compared to control infected midguts (Figs. 1L and EV1I), suggesting that EC apoptosis is required for ISC proliferation after midgut damage by *P.e*. infection.

Draper is an immunoreceptor known to detect phosphatidylserine on apoptotic cells (Melcarne et al, 2019; Tung et al, 2013). Because Draper is highly expressed on the plasma membrane of progenitors (Fig. 1G,G’) and the levels of activated Src increased in progenitors after *P.e*. infection (Fig. 1H–J), we determined if phosphatidylserine on dying ECs could be detected by ISCs and affect their proliferation. To test this, we masked externalised phosphatidylserine on dying ECs by expressing milk fat globule EGF-8 (Hakim-Mishnaevski et al, 2019; Tung et al, 2013). To test if phosphatidylserine exposure on ECs was important for ISC proliferation after EC ablation, we co-expressed milk fat globule EGF-8 and the pro-apoptotic factor, Reaper. As expected, we found an increase in ISC proliferation after EC ablation but also found that co-expressing milk fat globule EGF-8 decreased ISC proliferation by 55.5% (Fig. 1M). These data suggest that neighbouring ISCs may sense exposed phosphatidylserine on apoptotic ECs in the microenvironment. Since we had shown that *P.e*. infection could induce apoptosis in the midgut epithelium, we tested whether phosphatidylserine externalisation on ECs was required for ISC proliferation after midgut damage by *P.e*. infection. Masking externalised phosphatidylserine on ECs with milk fat globule EGF-8 decreased ISC proliferation after *P.e*. infection by 54.6% compared to control infected midguts (Fig. 1N). In contrast, we found that depleting another Draper ligand, Pretaporter (Kuraishi et al, 2009), in ECs, did not inhibit ISC proliferation after *P.e*. infection (Fig. EV1J). Together, these data indicate that externalised phosphatidylserine on apoptotic ECs is required for ISC proliferation after midgut damage.

### Draper-Src-Shark signalling promotes ISC proliferation after midgut damage

**Figure 2:**
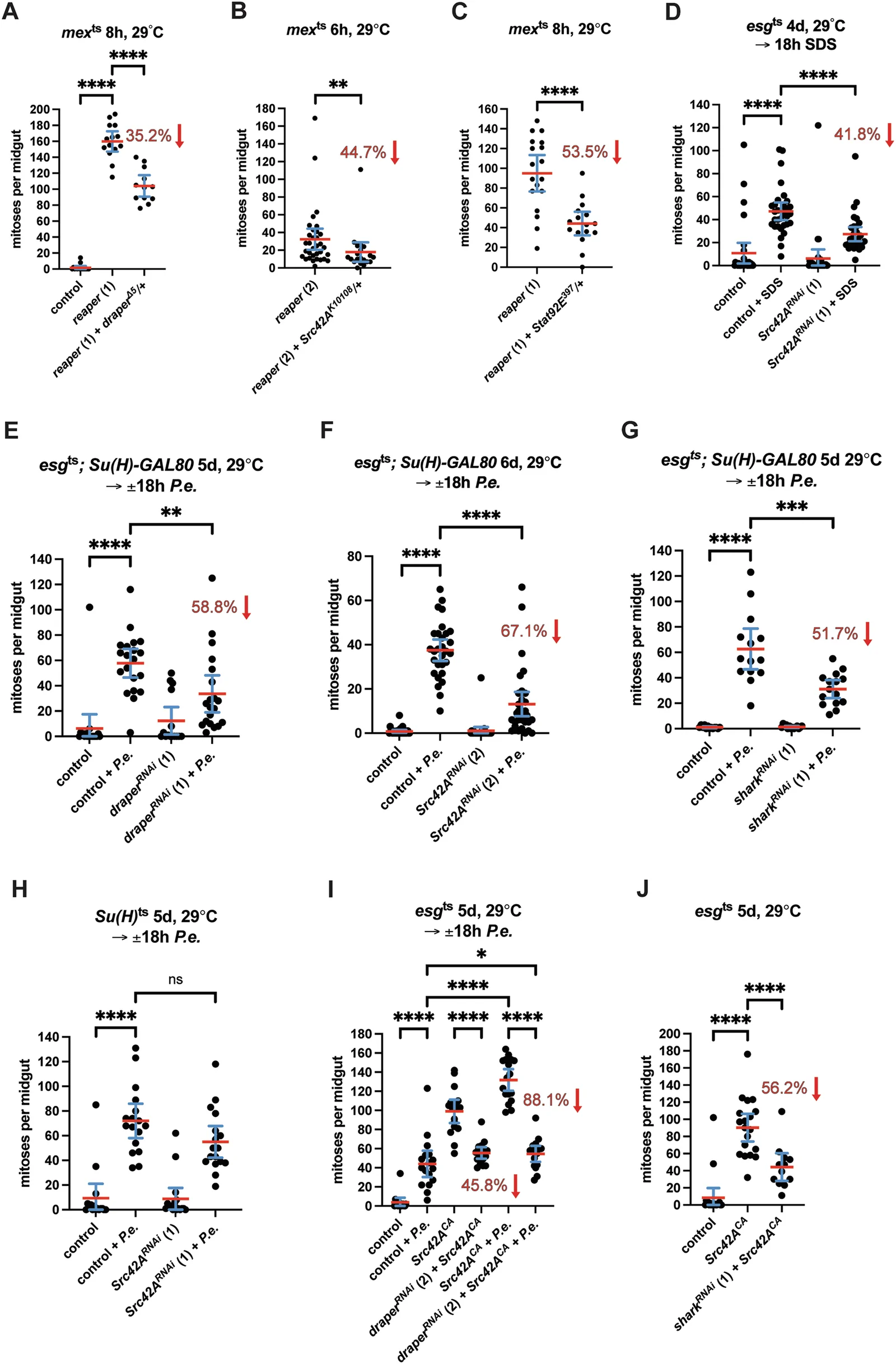
Draper-Src42A-Shark signalling promotes adult *Drosophila* intestinal stem cell proliferation after midgut damage. Draper, Src42A and Stat92E are required to promote ISC proliferation after enterocyte (EC) apoptosis (**A**–**C**). The mean mitoses per midgut increased in midguts expressing *reaper* in ECs (*n* = 14 midguts from one of two experiments) compared to control midguts (*n* = 16 from one of two experiments) (Mann–Whitney; *P* < 0.0001) (**A**). The mean mitoses per midgut increased in midguts expressing *reaper* in ECs (*n* = 33 midguts from one experiment (**B**) and *n* = 18 midguts from one of two experiments (**C**)). Mean mitoses per midgut decreased in *draper*^*Δ5/+*^ (*n* = 13 midguts from one of two experiments), *Src42A*^*K10108/+*^ (*n* = 20 midguts from one experiment) or *Stat92E*^*397/+*^ (*n* = 16 midguts from one of two experiments) heterozygous midguts expressing *reaper* in ECs compared to midguts expressing *reaper* alone in ECs (Mann–Whitney; *P* < 0.0001, *P* = 0.0065 and *P* < 0.0001, A-C, respectively) (**A**–**C**). Src42A is required in progenitors to promote ISC proliferation after detergent-induced damage (**D**). The mitoses per midgut increased in SDS-fed control midguts (*n* = 30 midguts from two experiments) compared to undamaged control midguts (*n* = 30 midguts from two experiments) (Mann–Whitney; *P* < 0.0001). The mean mitoses per midgut decreased in SDS-fed midguts expressing *Src42A*^*RNAi*^ in progenitors (*n* = 30 midguts from two experiments) compared to SDS-fed control midguts (Mann–Whitney; *P* < 0.0001). Draper, Src42A and Shark promote ISC proliferation after midgut damage (**E**–**G**). The mean mitoses increased in *P.e*.-infected control midguts (*n* = 20 midguts from one of two experiments (**E**), *n* = 29 midguts from two experiments (**F**) and *n* = 14 midguts from one of two experiments (**G**)) compared to uninfected control midguts (*n* = 19 midguts from one of two experiments (**E**), *n* = 29 midguts from two experiments (**F**) and *n* = 13 midguts from one of two experiments (**G**)) (Mann–Whitney; *P* < 0.0001 (**E**), *P* < 0.0001 (**F**) and *P* < 0.0001 (**G**)). The mean mitoses per midgut decreased in *P.e*.-infected midguts expressing *draper*^*RNAi*^, *Src42A*^*RNAi*^ or *shark*^*RNAi*^ in ISCs (*n* = 20 midguts from one of two experiments (**E**), *n* = 32 midguts from two experiments (**F**) and *n* = 15 midguts from one of two experiments (**G**)) compared to *P.e*.-infected control midguts (Mann–Whitney; *P* = 0.0026 (**E**), *P* < 0.0001 (**F**) and *P* = 0.0003 (**G**)). Src42A is not required in EBs to promote ISC proliferation after midgut damage (**H**). The mean mitoses per midgut increased in *P.e*.-infected control midguts (*n* = 17 midguts from one of two experiments) compared to uninfected control midguts (*n* = 16 midguts from one of two experiments) (Mann–Whitney; *P* < 0.0001). The mean mitoses per midgut did not significantly decrease in *P.e*.-infected midguts expressing *Src42A*^*RNAi*^ in EBs (*n* = 17 midguts from one of two experiments) compared to *P.e*.-infected control midguts (Mann–Whitney; *P* < 0.0585). Draper and Shark are required for Src42A to promote ISC proliferation (**I**, **J**). The mean mitoses per midgut increased in *P.e*.-infected midguts (*n* = 17 midguts from one of two experiments) and in midguts expressing constitutively active Src42A (*Src42A*^*CA*^) in progenitors (*n* = 17 midguts from one of two experiments) compared to control midguts (*n* = 15 midguts from one of two experiments) (Mann–Whitney; *P* < 0.0001 and *P* < 0.0001, respectively) (**I**). The mean mitoses per midgut decreased in midguts expressing *draper*^*RNAi*^ and *Src42A*^*CA*^ in progenitors (*n* = 17 midguts from one of two experiments) compared to midguts with progenitors expressing *Src42A*^*CA*^ alone (Mann–Whitney; *P* < 0.0001). The mean mitoses per midgut further increased in *P.e*.-infected midguts expressing *Src42A*^*CA*^ in progenitors (*n* = 17 midguts from one of two experiments) compared to control *P.e*.-infected midguts (Mann–Whitney; *P* < 0.0001). The mean mitoses per midgut strongly decreased in *P.e*.-infected midguts expressing *draper*^*RNAi*^ and *Src42A*^*CA*^ in progenitors (*n* = 17 midguts from one of two experiments) compared to *P.e*.-infected midguts expressing *Src42A*^*CA*^ in progenitors (Mann–Whitney; *P* < 0.0001) (**I**). The mean mitoses per midgut increased in midguts expressing *Src42A*^*CA*^ (*n* = 19 midguts from one of two experiments) compared to control midguts (*n* = 20 midguts from one of two experiments) (Mann–Whitney; *P* < 0.0001) (**J**). The mean mitoses per midgut decreased in midguts expressing *shark*^*RNAi*^ and *Src42A*^*CA*^ in progenitors (*n* = 12 midguts from one of two experiments) compared to midguts expressing *Src42A*^*CA*^ alone in progenitors (Mann–Whitney; *P* < 0.0001) (**J**). Mean (red line) and 95% confidence interval (blue bars) are shown. Significance levels are indicated by *P ≤ 0.05, **P ≤ 0.01, ***P ≤ 0.001, ****P ≤ 0.0001, and ns, for not significant (*P* > 0.05). Source data are available online for this figure.

Since externalised phosphatidylserine on dying ECs was required for ISC proliferation, and Draper is highly expressed on progenitors, we determined whether Draper was required for ISC proliferation after EC ablation. To do this, we expressed *reaper* in ECs with *mex*^ts^ in *draper* heterozygotes. Whereas EC ablation increased ISC proliferation, we found that ISC proliferation decreased by 35.2% after EC ablation in midguts of *draper* heterozygotes (Fig. 2A). These data suggest that Draper is required for ISC proliferation after EC ablation. Since we found that pSrc accumulates cortically in progenitors after *P.e*. infection, possibly phosphorylating the ITAM in Draper, we similarly tested whether Src42A was required for ISC proliferation after EC ablation. Indeed, ISC proliferation decreased by 44.7% in midguts of *Src42A* heterozygotes after EC ablation (Fig. 2B). The expression of constitutively active Src42A in midgut progenitors strongly induces ISC proliferation that requires STAT (Cordero et al, 2014; Kohlmaier et al, 2015). Furthermore, in adult *Drosophila* glia, Src42A signalling can activate STAT to promote phagocytosis of injured axons of ORNs (Doherty et al, 2014). Thus, we also tested whether STAT was required for ISC proliferation after EC ablation. ISC proliferation decreased in midguts of *Stat92E* heterozygotes by 53.5% after EC ablation (Fig. 2C). Together, these data suggest that phosphatidylserine exposure on apoptotic ECs activates Draper-Src-STAT signalling that is required for ISC proliferation. Because Draper is highly expressed in progenitors, Draper-Src-STAT signalling is likely to be required in progenitors to recognise exposed phosphatidylserine on midgut epithelial cells after damage.

**Figure EV2:**
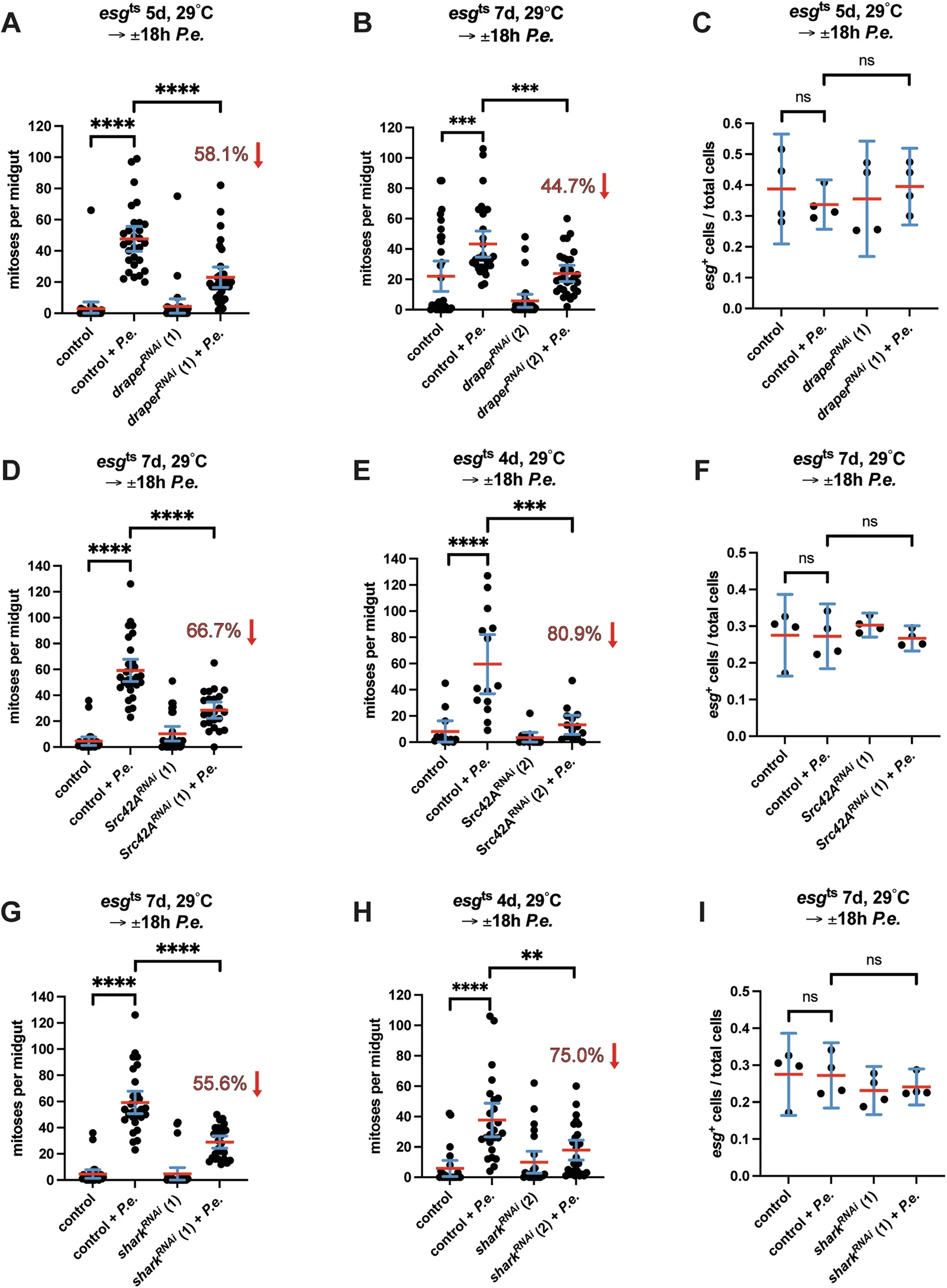
Draper-Src-Shark signalling in progenitors promotes ISC proliferation after midgut damage. Draper is required in progenitors to promote ISC proliferation after *P.e*. infection (**A**, **B**). The mean mitoses per midgut increased in *P.e*.-infected control midguts (*n* = 29 midguts from two experiments (**A**) and *n* = 31 midguts from two experiments (**B**)) compared to uninfected control midguts (*n* = 31 midguts from two experiments (**A**) and *n* = 32 midguts from two experiments (**B**)) (Mann–Whitney; *P* < 0.0001 (**A**) and *P* = 0.0009 (**B**)). The mean mitoses per midgut decreased in *P.e*.-infected midguts expressing *draper*^*RNAi*^ in progenitors (*n* = 32 midguts from two experiments (**A**) and *n* = 30 midguts from two experiments (**B**)) compared to *P.e*.-infected control midguts (Mann–Whitney; *P* < 0.0001 (**A**) and *P* = 0.0001 (**B**)). Depleting *draper* from progenitors does not affect their number after *P.e*.-infection (**C**). There was no significant difference between the mean ratio of *esg*^*+*^ cells/total cells in *P.e*.-infected control midguts (*n* = 4 midguts from one of two experiments) compared to uninfected control midguts (*n* = 4 midguts from one of two experiments) (unpaired *t* test; *P* = 0.4419). The mean ratio of *esg*^*+*^ cells/total cells was similar in *P.e*.-infected midguts expressing *draper*^*RNAi*^ in progenitors (*n* = 4 midguts from one of two experiments) compared to *P.e*.-infected control midguts (unpaired *t* test; *P* = 0.2557). Src is required in progenitors to promote ISC proliferation after *P.e*. infection (**D**, **E**). The mean mitoses per midgut increased in *P.e*.-infected control midguts (*n* = 29 midguts from two experiments (**D**) and *n* = 14 midguts from one experiment (**E**)) compared to uninfected control midguts (*n* = 27 midguts from two experiments (**D**) and *n* = 13 midguts from one experiment (**E**)) (Mann–Whitney; *P* < 0.0001 (**D**) and *P* < 0.0001 (**E**)). The mean mitoses per midgut decreased in *P.e*.-infected midguts expressing *Src42A*^*RNAi*^ in progenitors (*n* = 22 midguts from two experiments (**D**) and *n* = 14 midguts from one experiment (**E**)) compared to *P.e*.-infected control midguts (Mann–Whitney; *P* < 0.0001 (**D**) and *P* = 0.0001 (**E**)). Depleting *Src42A* in progenitors does not affect their number after *P.e*. infection (**F**). There was no significant difference between the mean ratio *esg*^*+*^ cells/total cells in *P.e*.-infected control midguts (*n* = 4 midguts from one of two experiments) compared to uninfected control midguts (*n* = 4 midguts from one of two experiments) (unpaired *t* test; *P* = 0.9484). The mean ratio *esg*^*+*^ cells/total cells was similar in *P.e*.-infected midguts expressing *Src42A*^*RNAi*^ in progenitors (*n* = 4 midguts from one of two experiments) compared to *P.e*.-infected control midguts (unpaired *t* test; *P* = 0.8602). Shark is required in progenitors to promote ISC proliferation after *P.e*. infection (**G**, **H**). The mean mitoses per midgut increased in *P.e*.-infected control midguts (*n* = 29 midguts from two experiments (**G**) and *n* = 25 midguts from two experiments (**H**)) compared to uninfected control midguts (*n* = 27 midguts from two experiments (**G**) and *n* = 23 midguts from two experiments (**H**)) (Mann–Whitney; *P* < 0.0001 (**G**) and *P* < 0.0001 (**H**)). The mean mitoses per midgut decreased in *P.e*.-infected midguts expressing *shark*^*RNAi*^ in progenitors (*n* = 27 midguts from two experiments (**G**) and *n* = 27 midguts from two experiments (**H**)) compared to *P.e*.-infected control midguts (Mann–Whitney; *P* < 0.0001 (**G**) and *P* = 0.0013 (**H**)). Depleting *shark* in progenitors does not affect their number after *P.e*.-infection (**I**). There was no significant difference between the mean ratio of *esg*^*+*^ cells/total cells in *P.e*.-infected control midguts (*n* = 4 midguts from one of two experiments) compared to uninfected control midguts (*n* = 4 midguts from one of two experiments) (unpaired *t* test; *P* = 0.9484). The mean ratio *esg*^*+*^ cells/total cells in *P.e*.-infected midguts expressing *shark*^*RNAi*^ in progenitors (*n* = 4 midguts from one of two experiments) was similar to the ratio in *P.e*.-infected control midguts (unpaired *t* test; *P* = 0.3649). Mean (red line) and 95% confidence intervals (blue bars) are shown. Significance levels are indicated by *P ≤ 0.05, **P ≤ 0.01, ***P ≤ 0.001, ****P ≤ 0.0001, and ns, for not significant (*P* > 0.05). Source data are available online for this figure.

Since Draper is highly expressed in midgut progenitors and both Draper and Src42A are required for ISC proliferation after EC ablation, we tested whether Draper, Src42A and a known effector of Draper signalling, Shark, are required specifically in progenitors for ISC proliferation after damage by depleting *draper, Src42A* and *shark* from midgut progenitors with *esg*^ts^. We found that Draper, Src42A and Shark are required in progenitors for ISC proliferation after *P.e*. infection. Depleting *draper* from progenitors by RNAi with *esg*^ts^ resulted in a 58.1% and 44.7% (RNAi (1) and RNAi (2), respectively) decrease in ISC proliferation after *P.e*. infection compared to infected control midguts (Fig. EV2A,B). Src42A was previously shown to be required in midgut progenitors for ISC proliferation after *P.e*. infection (Cordero et al, 2014; Kohlmaier et al, 2015). We confirmed that depletion of *Src42A* from progenitors with *esg*^ts^ blocked ISC proliferation after *P.e*. infection by 66.7% and 80.9%, for RNAis (1) and (2), respectively (Fig. EV2D,E). Further, depleting *shark* in progenitors by RNAi with *esg*^ts^ resulted in a 55.6% and 75.0% (RNAi (1) and (2), respectively) decrease in ISC proliferation after *P.e*. infection compared to infected control midguts (Fig. EV2G,H). Moreover, the decrease in ISC proliferation after depleting *draper*, *Src42A* and *shark* within progenitors and *P.e*. infection was not due to reduced progenitor cell number (Fig. EV2C,F,I). Together, these data indicate that Draper-Src-Shark signalling in progenitors promotes adult *Drosophila* midgut regeneration upon damage.

Src42A is required in midgut progenitors for ISC proliferation after *P.e*. infection (Cordero et al, 2014; Kohlmaier et al, 2015); however, it is not known whether Src42A is required for ISC proliferation after other types of midgut damage, such as chemical stress. To ascertain this, we depleted *Src42A* in progenitors with *esg*^ts^ and fed these flies the detergent, sodium dodecyl sulfate (SDS) (Patel et al, 2019). While SDS exposure increased ISC proliferation in control midguts, we found that *Src42A* depletion in progenitors blocked ISC proliferation by 41.8% after detergent-induced damage (Fig. 2D). Together, these data suggest that Draper-Src-Shark signalling is required in progenitors to sense midgut damage caused by pathogenic bacterial infection and chemical (detergent) stress.

*esg*^*+*^ midgut progenitors include ISCs, EBs and EEps. Thus, it was uncertain in which progenitor cell type Draper-Src-Shark signalling was required for ISC proliferation after *P.e*.-induced damage. To determine this, we depleted *draper*, *Src42A* and *shark* from ISCs using an ISC-specific driver, *esg-GAL4 Su(H)-GAL80 tubGAL80*^ts^ (*esg*^ts^ *Su(H)-GAL80*) (Wang et al, 2014). We found that *draper*, *Src42A* and *shark* depletion in ISCs strongly blocked ISC proliferation after *P.e*. by 58.8%, 67.1% and 51.7%, respectively (Fig. 2E–G). These data indicate that Draper, Src42A and Shark act mainly in ISCs to respond to midgut damage by proliferating. To determine whether Draper-Src-Shark signalling in EBs regulated ISC proliferation after midgut damage, we depleted *Src42A* in EBs using an EB-specific driver, *Su(H)-GAL4 tubGAL80*^ts^ (*Su(H)*^ts^)(Zeng et al, 2010). We found that EB-specific depletion of *Src42A* did not significantly decrease ISC proliferation after *P.e*. infection (Fig. 2H). These data suggest that Src42A acts mainly in ISCs to respond to midgut damage.

Src family kinases are inhibited by C-terminal tyrosine phosphorylation by the C-terminal Src kinase (Csk). Expression of a constitutively active Src42A (Y511F), which is refractory to Csk, in progenitors with *esg*^ts^ stimulates ISC proliferation in the absence of damage (Cordero et al, 2014; Kohlmaier et al, 2015). To determine whether Draper and Shark are required for Src42A to promote ISC proliferation, we depleted *draper* and *shark* in progenitors with RNAi and simultaneously expressed constitutively active Src42A in progenitors using *esg*^ts^. As expected, expression of constitutively active Src42A in progenitors stimulated ISC proliferation (Fig. 2I,J). However, in combination with an RNAi towards *draper* or *shark* in progenitors, ISC proliferation was blocked by 45.8% and 56.2%, respectively, compared to constitutively active Src42A expression alone (Fig. 2I,J). These data suggest that Draper-Shark signalling is partially required for Src42A to promote ISC proliferation. The binding of phosphatidylserine to Draper is thought to activate it by making its ITAM amenable to phosphorylation by Src42A (Williamson and Vale, 2018). Thus, we wondered whether damaging the midgut, and thus causing phosphatidylserine exposure, influenced constitutively active Src42A signalling through Draper. To test this, we expressed constitutively active Src42A in progenitors with *esg*^ts^ in *P.e*.-infected midguts. As expected, we observed an increase in ISC proliferation in *P.e*.-infected midguts and a further increase in ISC proliferation in infected midguts expressing constitutively active Src42A in progenitors. Interestingly, we found, however, an 88.1% decrease in ISC proliferation in infected midguts where we depleted *draper* and simultaneously expressed constitutively active Src42A in progenitors compared to infected midguts expressing constitutively active Src42A alone (Fig. 2I). These data indicate that unlike in unchallenged midguts, constitutively active Src42A primarily drives ISC proliferation through Draper in damaged midguts.

### Draper-Src-Shark signalling promotes ISC proliferation through STAT activation

JAK-STAT signalling is crucial for adult *Drosophila* ISCs to proliferate after midgut damage by pathogenic bacteria (Buchon et al, 2009a; Jiang et al, 2009). STAT activity can be observed in the midgut using the reporter, *10X STAT-dGFP* (STAT-GFP)(Bach et al, 2007; Beebe et al, 2010; Jiang et al, 2009). This reporter consists of multimerised 10X STAT92E binding sites fused together with a destabilised GFP. STAT activity has been observed in progenitors in homeostatic midguts but not within ECs and EEs (Beebe et al, 2010; Jiang et al, 2009). Further, JAK-STAT signalling increases within midgut progenitors and the visceral muscle after pathogenic bacterial infection (Jiang et al, 2009; Osman et al, 2012; Zhou et al, 2013).

**Figure 3:**
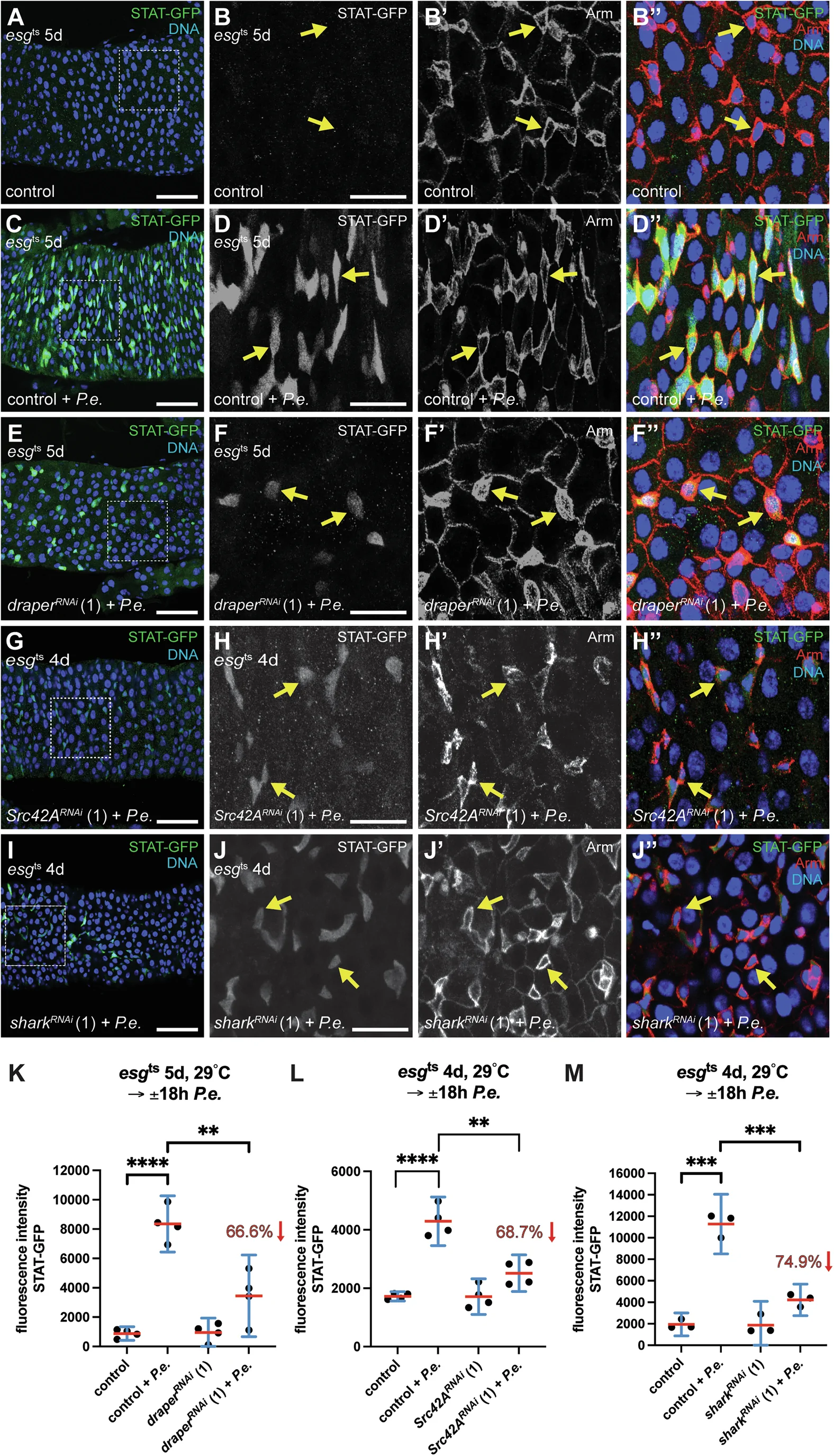
Draper-Src-Shark signalling promotes STAT activity in progenitors after midgut damage. STAT is activated in progenitors (*esg*^+^, Arm^high^; red; yellow arrows) after *P.e*. infection (**A**–**D**). STAT activity (STAT-GFP (*10X STAT-dGFP*); green) increased in progenitors of *P.e*.-infected control midguts (**C**–**D”**; green) compared to uninfected control midguts (**A**–**B”**; green). Draper, Src42A and Shark are required to activate STAT in progenitors after *P.e*. infection (**E**–**M**). STAT activation decreased in progenitors of *P.e*.-infected midguts expressing either *draper*^*RNAi*^ (**E**–**F”**; green), *Src42A*^*RNAi*^ (**G**–**H”**; green) or *shark*^*RNAi*^ (**I**–**J”**; green) in progenitors compared to *P.e*.-infected control midguts (**C**–**D”**; green). Mean STAT activity levels increased in progenitors of *P.e*.-infected control midguts (*n* = 4 midguts from one of two experiments (**K**), *n* = 4 midguts from one of two experiments (**L**) and *n* = 3 midguts from one of three experiments (**M**)) compared to uninfected control midguts (*n* = 4 midguts from one of two experiments (**K**), *n* = 4 midguts from one of two experiments (**L**) and *n* = 3 from one of three experiments (**M**)) (unpaired *t* tests; *P* < 0.0001 (**K**), *P* < 0.0001 (**L**) and *P* = 0.0002 (**M**)). Mean STAT activity decreased in progenitors of *P.e*.-infected midguts expressing *draper*^*RNAi*^, *Src42A*^*RNAi*^ or *shark*^*RNAi*^ with *esg*^ts^ (*n* = 4 midguts from one of two experiments (**K**), *n* = 4 midguts from one of two experiments (**L**) and *n* = 3 midguts from one of three experiments (**M**)) compared to infected control midguts (unpaired *t* test; *P* = 0.0036 (**K**), *P* = 0.0016 (**L**) and *P* = 0.0006 (**M**)). STAT activity in uninfected midguts expressing *draper*^*RNAi*^, *Src42A*^*RNAi*^ or *shark*^*RNAi*^ in progenitors (*n* = 4 midguts from one of two experiments (**K**), *n* = 4 midguts from one of two experiments (**L**) and *n* = 3 midguts from one of three experiments (**M**)) was similar to uninfected control midguts (**K**–**M**). Mean (red line) and 95% confidence interval (blue bars) are shown. DNA is in blue in (**A**, **B”**, **C**, **D”**, **E**, **F”**, **G**, **H”**, **I**, **J”**). Scale bars in (**A**, **C**, **E**, **G**, **I**) are 50 μm and in (**B**, **D**, **F**, **H**, **J**) are 20 μm. Significance levels are indicated by *P ≤ 0.05, **P ≤ 0.01, ***P ≤ 0.001, ****P ≤ 0.0001, and ns, for not significant (*P* > 0.05). Controls for *draper*^*RNAi*^, *Src42A*^*RNAi*^ and *shark*^*RNAi*^ are shown in Fig. EV3. Histograms of STAT activity per progenitor for all genotypes are shown in Appendix Fig. S1. Source data are available online for this figure.

**Figure EV3:**
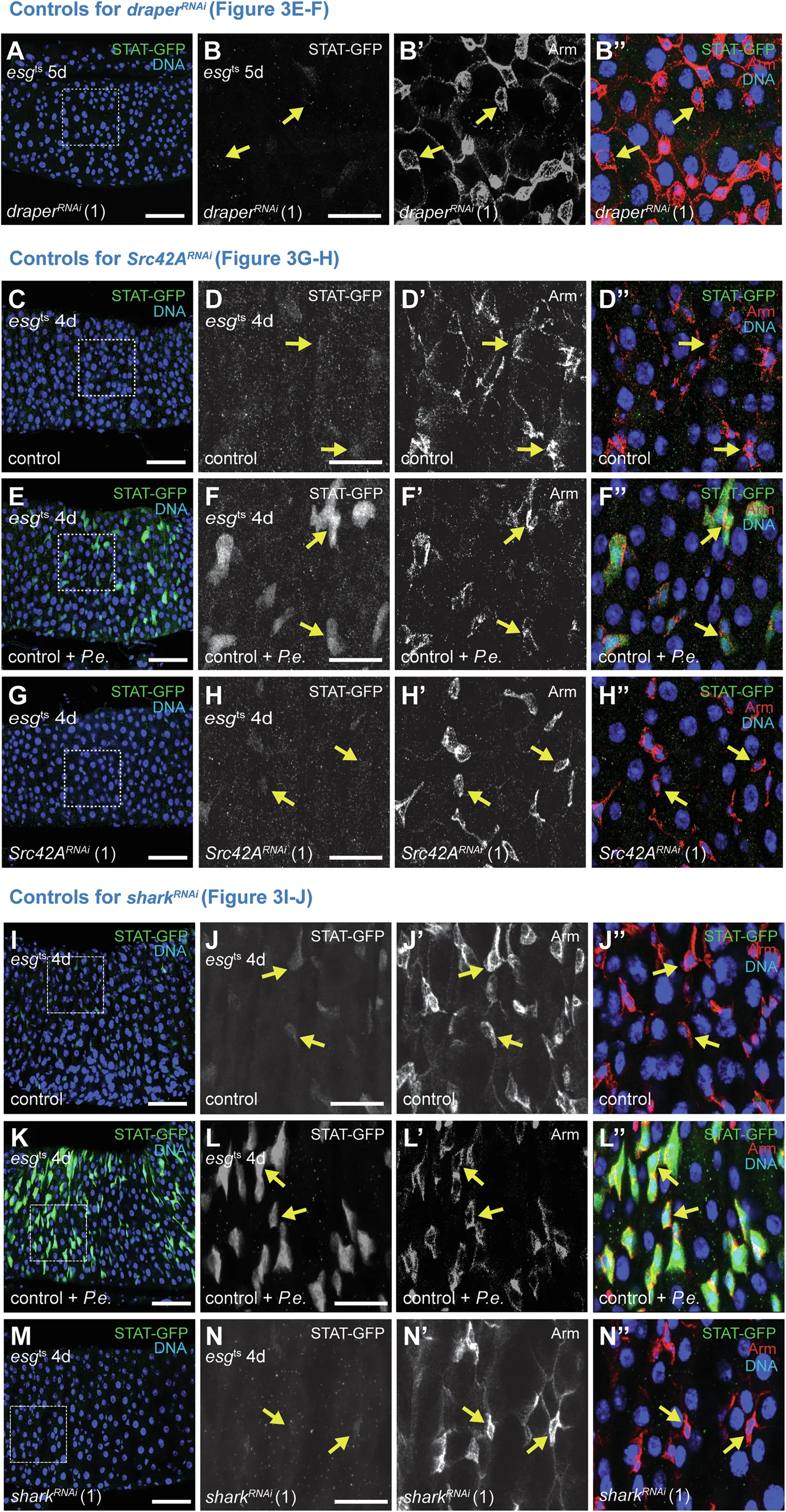
Draper-Src-Shark signalling promotes STAT activity in progenitors after midgut damage; inhibiting Draper-Src-Shark signalling does not affect basal STAT activity in progenitors. Draper does not affect basal STAT activity in progenitors (**A**, **B**). STAT activity (STAT-GFP (*10X STAT-dGFP*); green) in progenitors (Arm^high^, red; yellow arrows) expressing *draper*^*RNAi*^ in uninfected midguts (**A**–**B”**) was similar to control uninfected midguts. STAT is activated in progenitors after *P.e*. infection (**C**–**F**, **I**–**L**). STAT activity increased in progenitors of *P.e*.-infected control midguts (**E**–**F”**, **K**–**L”**) compared to uninfected control midguts (**C**–**D”**, **I**–**J”**). Depleting Src and Shark in progenitors does not affect their basal STAT activity (**G**–**H**, **M**–**N**). STAT activity in progenitors expressing *Src42A*^*RNAi*^ in uninfected midguts (**G**–**H”**) was similar to that in uninfected control midguts (**C**–**D”**). STAT activity in progenitors expressing *shark*^*RNAi*^ in uninfected midguts (**M**–**N”**) was similar to that in uninfected control midguts (**I**–**J”**). Scale bars in (**A**, **C**, **E**, **G**, **I**, **K**, **M**) are 50 μm and in (**B**, **D**, **F**, **H**, **J**, **L**, **N**) are 20 μm. Significance levels are indicated by *P ≤ 0.05, **P ≤ 0.01, ***P ≤ 0.001, ****P ≤ 0.0001, and ns, for not significant (*P* > 0.05). Source data are available online for this figure.

**Figure EV4:**
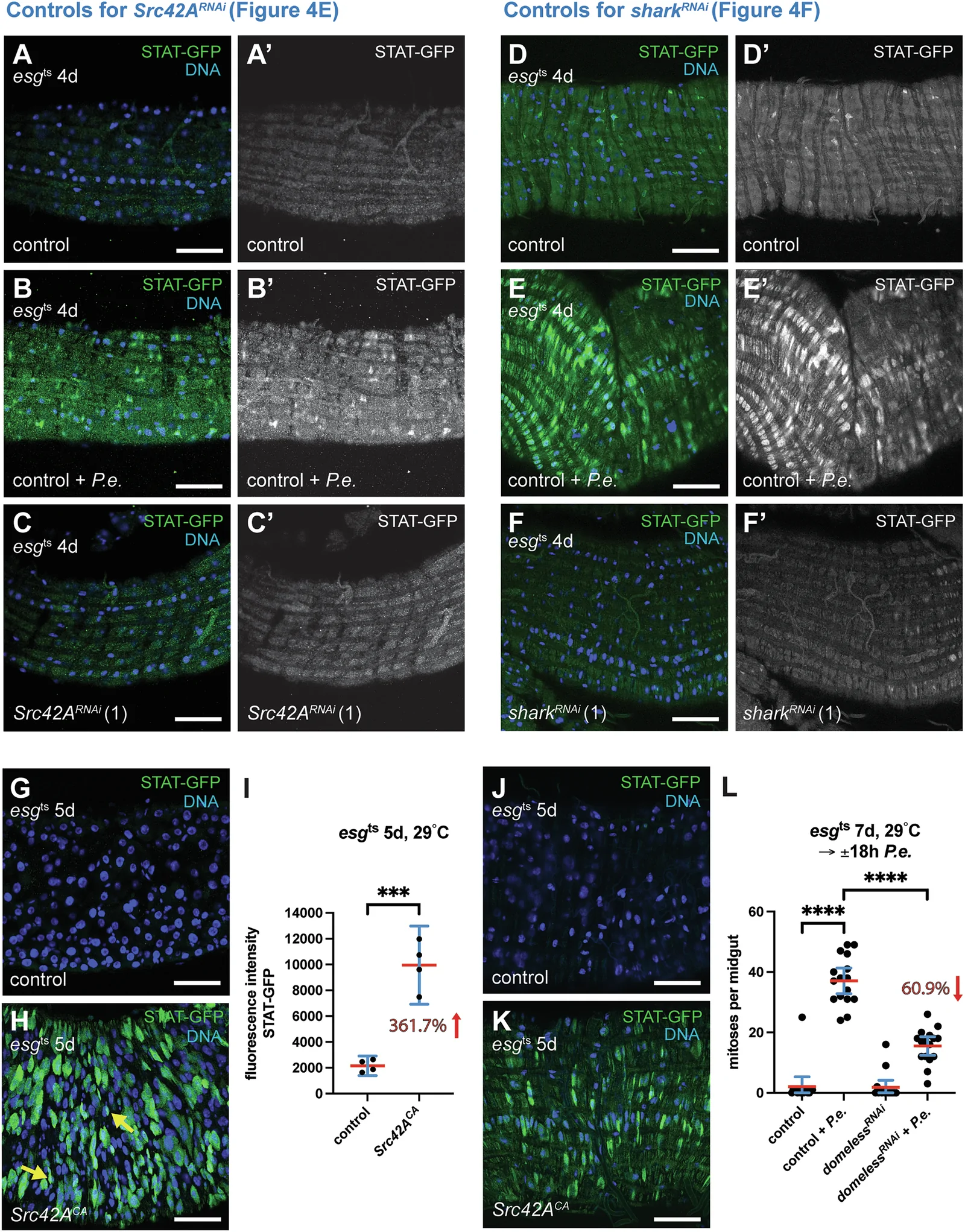
Draper-Src-Shark signalling is sufficient to increase STAT activity in progenitors and in the visceral muscle; inhibiting Src-Shark signalling in midgut progenitors does not affect basal STAT activity in the visceral muscle. STAT is activated in the visceral muscle (VM) after *P.e*. infection (**A**–**B**, **D**–**E**). STAT activity (STAT-GFP (*10X STAT-dGFP*); green) increased in the VM of *P.e*.-infected control midguts (**B**–**B’**, **E**–**E’**) compared to uninfected control midguts (**A**–**A’**, **D**–**D’**). Depleting *Src42A* and *shark* does not affect basal STAT activity in the VM (**C**, **F**). STAT activity in the VM of uninfected midguts expressing *Src42A*^*RNAi*^ or *shark*^*RNAi*^ in progenitors (**C**–**C’**, **F**–**F’**) was similar to the STAT activity in the VM of uninfected control midguts (**A**–**A’**, **D**–**D’**). Constitutively active Src42A signalling in progenitors induces STAT in both the progenitors and in the VM (**G**–**K**). STAT activation increased in progenitors (yellow arrows) expressing *Src42A*^*CA*^ (**H**) compared to control midguts (**G**). Mean STAT levels increased in progenitors expressing *Src42A*^*CA*^ (*n* = 4 midguts from one experiment) compared to those in control midguts (*n* = 4 midguts from one experiment) (unpaired *t* tests; *P* = 0.0002) (**I**). STAT activation increased in the VM of midguts expressing *Src42A*^*CA*^ in progenitors (**K**) compared to control midguts (**J**). Domeless is required in progenitors to promote ISC proliferation after midgut damage (**L**). The mean mitoses per midgut increased in *P.e*.-infected control midguts (*n* = 16 midguts from one experiment) compared to uninfected control midguts (*n* = 16 midguts from one experiment) (Mann–Whitney; *P* < 0.0001). The mean mitoses per midgut decreased in *P.e*.-infected midguts expressing *dome*^*RNAi*^ in progenitors (*n* = 16 midguts from one experiment) compared to *P.e*.-infected control midguts (Mann–Whitney; *P* < 0.0001). Mean (red line) and 95% confidence intervals (blue bars) are shown. Scale bars in (**A**, **B**, **C**, **D**, **E**, **F**, **G**, **H**, **J**, **K**) are 50 μm. Significance levels are indicated by *P ≤ 0.05, **P ≤ 0.01, ***P ≤ 0.001, ****P ≤ 0.0001, and ns, for not significant (*P* > 0.05). Source data are available online for this figure.

Given that expression of constitutively active Src42A in progenitors induces ISC proliferation, which requires STAT (Cordero et al, 2014; Kohlmaier et al, 2015), we wondered whether STAT activity in progenitors is regulated by Draper-Src42A-Shark signalling. To test whether Draper, Src42A and Shark are required for STAT activation in progenitors after *P.e*. infection, we depleted *draper, Src42A* and *shark* from progenitors with *esg*^ts^ and infected flies with *P.e*.. Midgut progenitors can be identified by their high levels of cortical ß-catenin/Armadillo (Arm), whereas Arm is found at the adherens junctions of EEs and ECs (Chen et al, 2018; Maeda et al, 2008). We therefore measured STAT-GFP within *esg*^*+*^ Arm^high+^ progenitors. We found an increase in STAT activity in the posterior midgut (R4-R5) after *P.e*. infection. Whereas STAT activity increased in the progenitors in *P.e*.-infected control midguts (Figs. 3C–D”,K–M and EV3E–F”,K–L”) compared to uninfected control midguts (Figs. 3A–B”,3K–M and EV3C–D”,I–J”), we found that STAT activity did not similarly increase in *draper*-, *Src42A-* and *shark*-depleted progenitors in *P.e.-*infected midguts (Fig. 3E–J”,K–M). Indeed, we found a 66.6%, 68.7% and 74.9% decrease in STAT activity in *draper-*, *Src42A*- and *shark*-depleted progenitors, respectively, after *P.e*. infection (Fig. 3K–M). Histogram analysis of STAT activity in progenitors showed an increase in STAT activity with *P.e*. infection in control midguts compared to progenitors in uninfected midguts (Appendix Fig. S1). However, progenitors depleted for *draper*, *Src42A* and *shark* had lower STAT activity after infection compared to progenitors in infected control midguts (Appendix Fig. S1). These data indicate that Draper-Src-Shark signalling promotes ISC proliferation through STAT activation. This is consistent with the requirement of STAT for ISC proliferation in midguts after EC ablation by *reaper* expression, which should activate Draper-Src-Shark signalling (Fig. 2C). We next determined whether Src42A signalling was sufficient to promote STAT activity in progenitors. STAT activity increased by 361.7% in progenitors expressing constitutively active Src42A (Fig. EV4G–I), indicating that Draper-Src-Shark signalling is sufficient to promote STAT activity. Interestingly, STAT activation decreased in both *esg*^*+*^ Arm^high+^ ISCs and EBs. These data further suggest that Draper-Src-Shark signalling cell-autonomously regulates STAT activity in EBs. Furthermore, we found similar levels of STAT activity in *draper*-, *Src42A*- and *shark*-depleted progenitors of uninfected midguts (Figs. 3K–M and EV3A–B”,G–H”,M–N”) as to progenitors in uninfected control midguts (Figs. 3A–B”,K–M and EV3C–D”,I–J”). These data suggest that Draper-Src-Shark signalling is not required for STAT activity and ISC proliferation during homeostasis and rather functions exclusively as a damage-sensing pathway.

**Figure 4:**
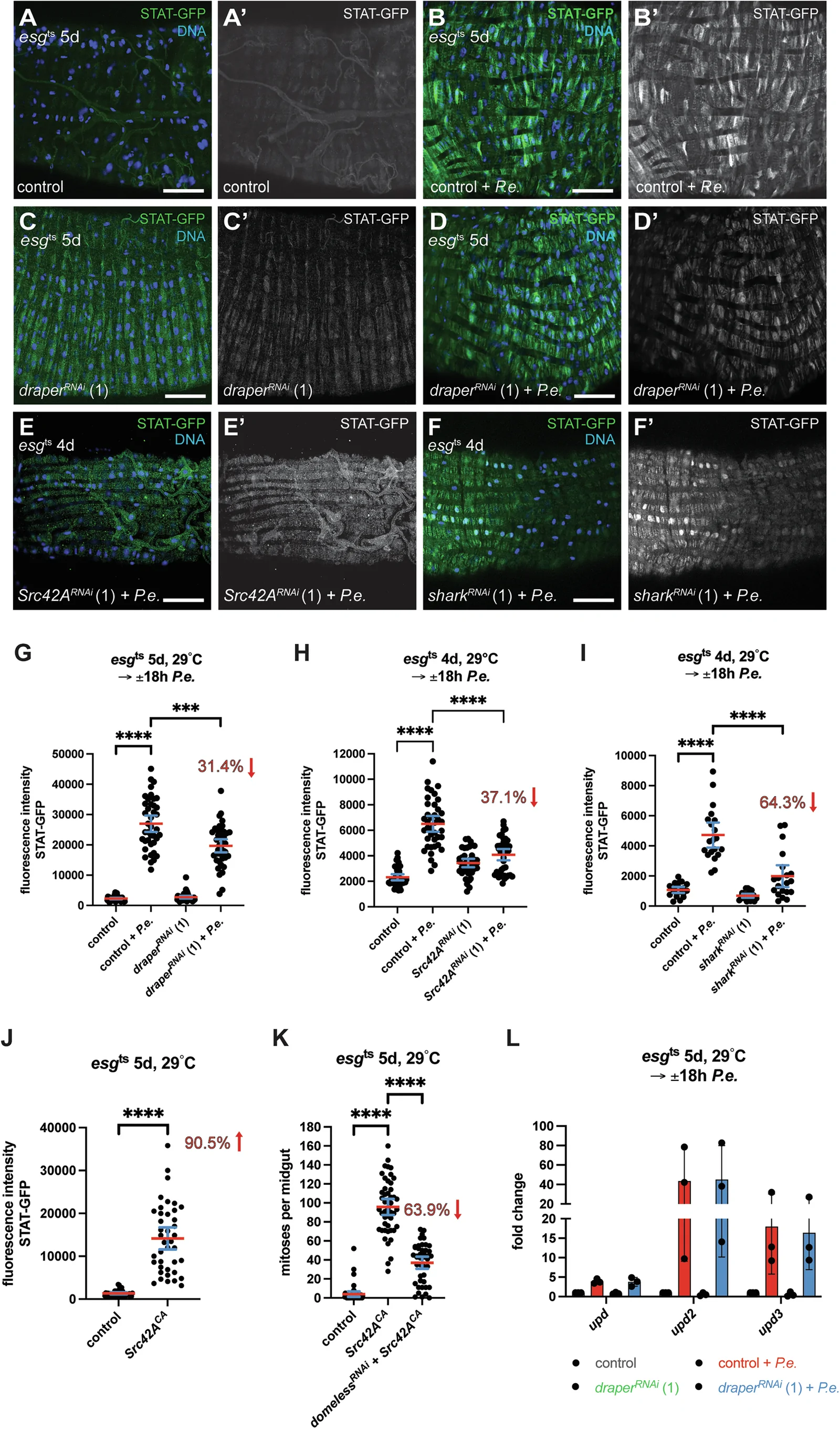
Draper-Src-Shark signalling in progenitors promotes STAT activation in the visceral muscle after midgut damage. STAT is activated in the visceral muscle (VM) after *P.e*.-induced damage (**A**, **B**). STAT activity (STAT-GFP (*10X STAT-dGFP*); green) increased in the VM of *P.e*.-infected control midguts (**B**–**B’**) compared to uninfected control midguts (**A**–**A’**). Draper, Src42A and Shark in progenitors are required to activate STAT in the VM after *P.e*. infection (**C**–**I**). STAT activation decreased in the VM of *P.e*.-infected midguts expressing *draper*^*RNAi*^ (**D**–**D’**; green), *Src42A*^*RNAi*^ (**E**–**E’**; green) or *shark*^*RNAi*^ (**F**–**F’**; green) in progenitors compared to *P.e*.-infected control midguts (**B**–**B’**; green). Mean STAT activity (STAT-GFP fluorescence intensity) increased in the VM of *P.e*.-infected control midguts (*n* = 40 cells from four midguts from one of two experiments (**G**), *n* = 40 cells from four midguts from one of two experiments (**H**) and *n* = 20 cells from two midguts from one of three experiments (**I**)) compared to uninfected control midguts (*n* = 40 cells from four midguts from one of two experiments (**G**), *n* = 40 cells from four midguts from one of two experiments (**H**) and *n* = 20 cells from two midguts from one of three experiments (**I**)) (Mann–Whitney; *P* < 0.0001 (**G**), *P* = 0.0001 (**H**) and *P* < 0.0001(**I**)). Mean STAT activity decreased in the VM of *P.e*.-infected midguts expressing *draper*^*RNAi*^, *Src42A*^*RNAi*^ or *shark*^*RNAi*^ in progenitors (*n* = 40 cells from four midguts from one of two experiments (**G**), *n* = 40 cells from four midguts from one of two experiments (**H**) and *n* = 20 cells from two midguts from one of three experiments (**I**)) compared to infected control midguts (Mann–Whitney; *P* = 0.0003 (**G**), *P* = 0.0001 (**H**) and *P* < 0.0001 (**I**)). Src42A signalling in progenitors activates STAT in the VM (**J**). Mean STAT activity (STAT-GFP fluorescence intensity) increased in the VM of midguts expressing *Src42A*^*CA*^ in progenitors (*n* = 40 cells from four midguts from one of two experiments) compared to control midguts (*n* = 40 cells from four midguts from one of two experiments) (Mann–Whitney; *P* < 0.0001). Src42A requires Domeless to promote ISC proliferation (**K**). The mean mitoses per midgut increased in midguts expressing *Src42A*^*CA*^ (*n* = 48 midguts from two of three experiments) compared to control midguts (*n* = 44 midguts from two of three experiments) (Mann–Whitney; *P* < 0.0001). The mean mitoses per midgut decreased in midguts expressing *dome*^*RNAi*^ and *Src42A*^*CA*^ in progenitors (*n* = 43 midguts from two of three experiments) compared to midguts expressing *Src42A*^*CA*^ alone in progenitors (Mann–Whitney; *P* < 0.0001). Draper signalling in progenitors does not regulate cytokine expression (*upd1-3*) after midgut damage (**L**). The mean fold change in *upd1-3* expression was 3.9, 43.5 and 18.0, respectively, in infected control midguts (*n* = 3 experiments) compared to uninfected control midguts (*n* = 3 experiments). The mean fold change in *upd1-3* expression was 3.8, 45.0 and 16.4, respectively, in infected midguts expressing *draper*^*RNAi*^ in progenitors (*n *= 3 experiments) compared to control uninfected midguts. Mean (red line) and 95% confidence interval (blue bars) are shown. DNA is in blue in (**A**, **B**, **C**, **D**, **E**, **F**). Scale bars in (**A**, **B**, **C**, **D**, **E**, **F**) are 50 μm. Significance levels are indicated by *P ≤ 0.05, **P ≤ 0.01, ***P ≤ 0.001, ****P ≤ 0.0001, and ns, for not significant (*P* > 0.05). Controls for *Src42A*^*RNAi*^ and *shark*^*RNAi*^ (**E**–**F’**) and images of STAT activation in the VM after *Src42A*^*CA*^ expression in progenitors are shown in Fig. EV4. Source data are available online for this figure.

We next examined STAT activation in the visceral muscle (VM)—part of the regenerative ISC microenvironment—of infected midguts. Compared to control uninfected midguts, STAT activity increased within midgut VM upon *P.e*.-infection (Figs. 4A–B’,G–I and EV4A–B’,D–E’). Surprisingly, we found a 31.4%, 37.1%, and 64.3% decrease in STAT activity in the VM of *P.e*.-infected midguts containing *draper*-, *Src42A-* and *shark*-depleted progenitors, respectively, compared to *P.e*.-infected control midguts (Figs. 4B–B’,D–F’,G–I and EV4B,B’,E,E’). Moreover, STAT activity was similar between the VM of control midguts and those in midguts with progenitors depleted for *draper*, *Src42A* and *shark* (Figs. 4A,A’,C,C’,G–I and EV4A,A’,C,C’,D,D’,F,F’), suggesting that Draper-Src42A-Shark signalling is required in progenitors of damaged midguts to regulate VM STAT activity. These data indicate that STAT activity in the VM is regulated cell non-autonomously by Draper-Src-Shark signalling in ISCs, EBs, or both ISCs and EBs after midgut damage. We next increased Draper-Src-Shark signalling in progenitors by expressing constitutively active Src42A with *esg*^ts^ and assessed STAT activity in the VM. This increased STAT activity in the VM by 90.5% compared to progenitors in control midguts (Figs. 4J and EV4J,K). These data indicate that Draper-Src-Shark signalling in progenitors is sufficient to regulate STAT activity non-autonomously in the VM.

Nevertheless, it was unclear how Draper-Src-Shark signalling modulates STAT activity in progenitors and in the VM. Src has been reported to promote ISC proliferation via STAT independently of cytokines (Cordero et al, 2014; Kohlmaier et al, 2015). As previously described (Buchon et al, 2009a; Jiang et al, 2009), we found that depletion of the cytokine receptor *domeless* in progenitors inhibited ISC proliferation by 60.9% after pathogenic bacterial infection (Fig. EV4L). Surprisingly, we found that simultaneously expressing constitutively active Src42A and depleting *domeless* in progenitors blocked ISC proliferation by 63.9% compared to progenitors expressing constitutively active Src42A alone (Fig. 4K). This suggests that Draper-Src-Shark signalling promotes STAT activation through Domeless signalling, possibly by regulating cytokine *upd1-3* expression in the midgut epithelium. To test if *upd1-3* expression is regulated by Draper signalling in progenitors, we performed quantitative PCR for *upds1-3* on infected midguts with progenitors depleted for *draper*. Draper signalling was not required in progenitors to regulate *upd1-3* expression in the midgut epithelium after *P.e*. infection (Fig. 4L). These data suggest that Draper signalling promotes STAT activity in progenitors and the VM through Domeless signalling, but without regulating *upds1-3* expression levels in the midgut epithelium.

Together, our data indicate that ISCs sense midgut damage using Draper-Src-Shark signalling, which promotes ISC proliferation through STAT activation. Furthermore, Draper-Src-Shark signalling in ISCs, EBs or in both progenitor cells non-autonomously activates STAT in the VM upon midgut damage. Moreover, our data suggest that STAT activation in both progenitors and the VM is potentially through cytokine-Domeless signalling.

### ISC Draper-Src-Shark signalling activates STAT without clearing apoptotic cells

**Figure 5:**
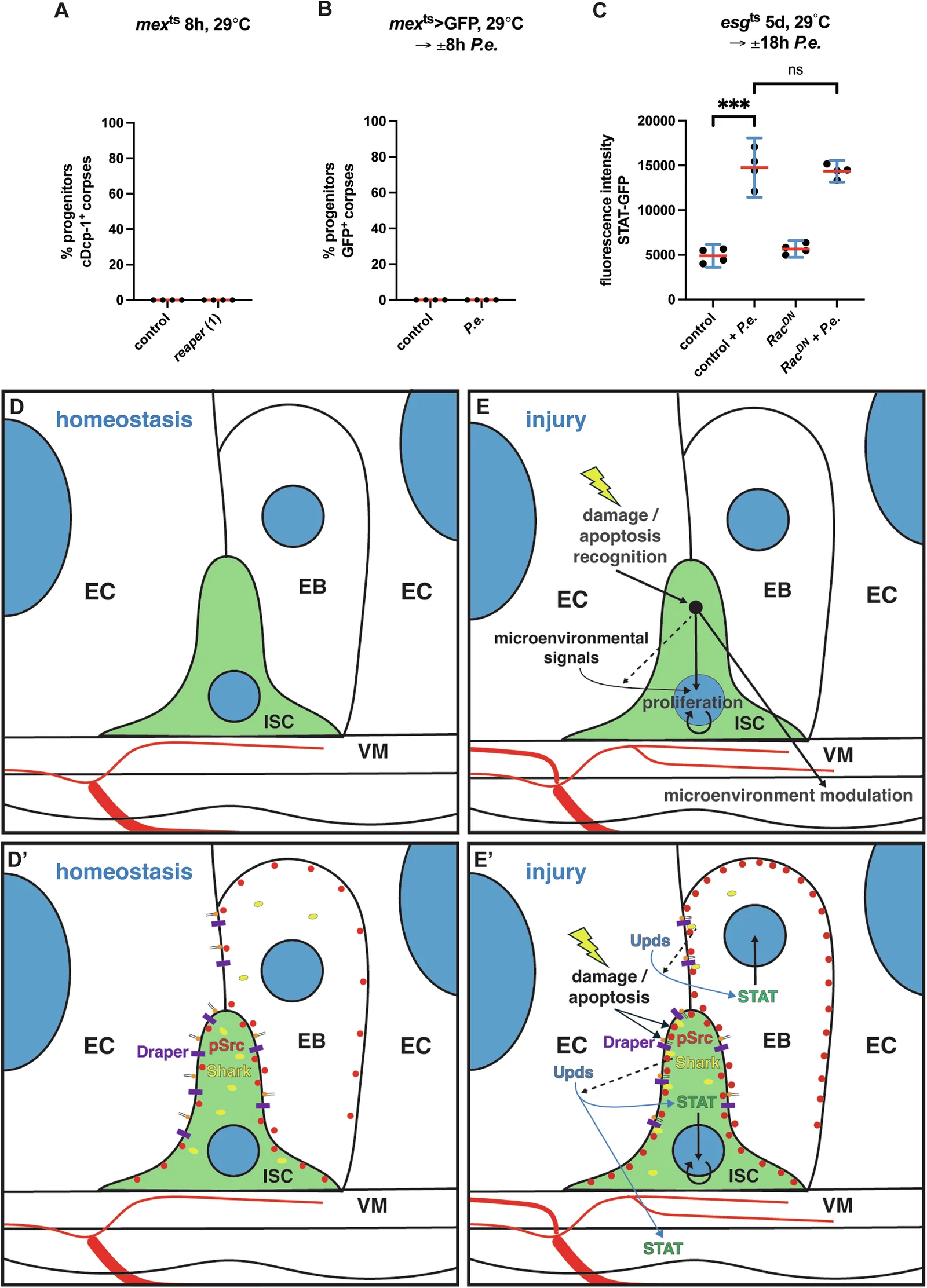
Progenitors do not clear apoptotic enterocytes after midgut damage. Cleaved Death Caspase-1 (cDcp-1)-positive enterocyte (EC) corpses are not cleared by progenitors (**A**). Mean percent of progenitors containing cDcp-1^+^ corpses in midguts expressing *reaper* in ECs (*n* = 4 midguts from one experiment) was similar to that of control midguts (*n* = 4 midguts from one experiment). EC corpses are not cleared by progenitors after *P.e*. infection (**B**). Mean percent of progenitors containing GFP^+^ corpses in midguts infected with *P.e*. (*n* = 4 midguts from one experiment) was similar to uninfected control midguts (*n* = 4 midguts from one experiment). Rac activity is not required for STAT activation in progenitors after *P.e*. infection (**C**). Mean STAT activity levels increased in progenitors of *P.e*.-infected control midguts (*n* = 4 midguts from one experiment) compared to uninfected control midguts (*n* = 4 midguts from one experiment) (unpaired *t* tests; *P* = 0.0001). Mean STAT activity level in progenitors of *P.e*.-infected midguts expressing *Rac*^*DN*^ with *esg*^ts^ (*n* = 4 midguts from one experiment) was similar to that in infected control midguts (unpaired *t* test; *P* = 0.7288). Damage recognition by Draper-Src-Shark-STAT signalling in intestinal stem cells promotes adult *Drosophila* midgut regeneration (**D**–**E’**). Under homeostasis, a low level of phosphorylated Src is found cortically within progenitors (ISCs and EBs). The immunoreceptor Draper is highly expressed on progenitors but does not detect phosphatidylserine in undamaged cells, and the Spleen tyrosine kinase homologue, Shark, is not recruited to Draper (**D**, **D’**). After injury, damage-induced apoptosis is recognised by ISCs, which stimulates their proliferation and modulates the regenerative microenvironment (**E**). Draper in progenitors detects exposed phosphatidylserine on dying enterocytes (ECs) after midgut damage, and a higher level of phosphorylated Src accumulates cortically in progenitors. Activated Src phosphorylates the immunoreceptor tyrosine-based activation motif (ITAM) in Draper, enabling Shark recruitment and activation. Draper-Src-Shark signalling in progenitors activates STAT both in progenitors and within the visceral muscle (VM) (**E’**). Mean (red line) and 95% confidence intervals (blue bars) are shown. Significance levels are indicated by *P ≤ 0.05, **P ≤ 0.01, ***P ≤ 0.001, ****P ≤ 0.0001, and ns, for not significant (*P* > 0.05). Images showing the absence of GFP^+^ corpses within progenitors after *P.e*. infection and STAT activity in progenitors expressing *Rac*^*DN*^ in *P.e.-*infected midguts are shown in Fig. EV5. Source data are available online for this figure.

**Figure EV5:**
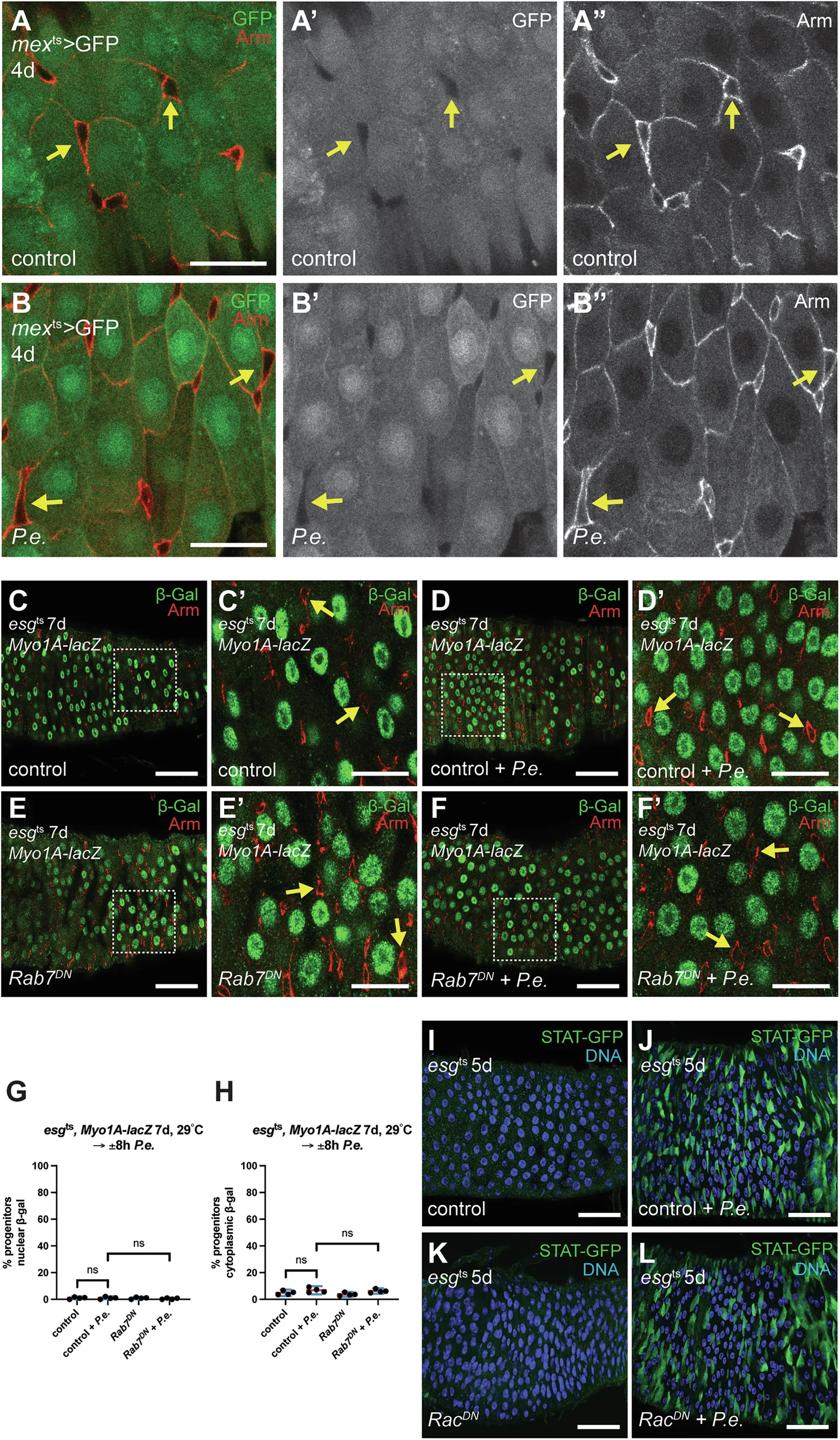
Midgut progenitors do not clear dying ECs; Rac activity does not promote STAT activity in progenitors after midgut damage. Dying enterocytes (ECs) are not cleared by progenitors after *P.e*. infection (**A**, **B**). GFP (green) expressed in ECs was not detected within Arm^high+^ (red, yellow arrows) cells in uninfected and *P.e*.-infected midguts (**A**–**B”**). EC corpses are not detected after blocking lysosomal degradation in progenitors after midgut damage (**C**–**H**). β-gal-positive (green) EC corpses were detected neither within progenitors (Arm^high^ (red); yellow arrows) of control uninfected and *P.e*.-infected midguts (**C**–**D’**) nor within *Rab7*^*DN*^-expressing progenitors of uninfected and infected midguts (**E**–**F’**). β-gal was detected within the nuclei of progenitors at the same frequency between uninfected (*n* = 4 midguts from one experiment) and infected control midguts (*n* = 4 midguts from one experiment) (Mann–Whitney; *P* > 0.9999) and between infected midguts with progenitors expressing *Rab7*^*DN*^ (*n* = 4 midguts from one experiment) and control infected midguts (Mann–Whitney; *P* = 0.3463) (**G**). β-gal was detected within the cytoplasm of progenitors at the same frequency between infected (*n* = 4 midguts from one experiment) and uninfected control midguts (*n* = 4 midguts from one experiment) (Mann–Whitney; *P* = 0.1522) and between infected midguts with progenitors expressing *Rab7*^*DN*^ (*n* = 4 midguts from one experiment) and control infected midguts (Mann–Whitney; *P* = 0.5942) (**H**). Rac signalling does not promote STAT activity in progenitors after *P.e*. infection (**I**–**L**). STAT activity (STAT-GFP (*10X STAT-dGFP*); green) increased in progenitors of *P.e*.-infected control midguts (**J**) compared to uninfected control midguts (**I**). STAT activity was similar between progenitors expressing *Rac*^*DN*^ in *P.e*.-infected midguts (**L**) and those in *P.e*.-infected control midguts (**J**). Mean (red line) and 95% confidence intervals (blue bars) are shown. Scale bars in (**A**, **B**, **C’**, **D’**, **E’**, **F’**) are 20 μm and in (**C, D, E, F**, **I**–**L**) are 50 μm. Single-slice images are presented in (**A–F'**). Significance levels are indicated by *P ≤ 0.05, **P ≤ 0.01, ***P ≤ 0.001, ****P ≤ 0.0001, and ns, for not significant (*P* > 0.05). Source data are available online for this figure.

Draper recognises apoptotic cells by detecting phosphatidylserine on the outer leaf of the plasma membrane (Ji et al, 2023; Melcarne et al, 2019; Tung et al, 2013; Williamson and Vale, 2018). Furthermore, Draper-Src-Shark signalling in glia can promote phagocytosis of axonal debris after adult ORN injury (MacDonald et al, 2006; Ziegenfuss et al, 2008). Thus, we tested whether progenitors with active Draper-Src-Shark signalling can clear apoptotic midgut ECs through phagocytosis. We examined whether cDcp-1^+^ apoptotic ECs can be engulfed by Arm^high+^ progenitors after EC ablation by expressing *reaper* with *mex*^ts^. Because apoptotic ECs would expose their phosphatidylserine, and progenitors have high Draper levels, we would expect the internalisation of apoptotic ECs by progenitors. Although we found 10.7% of apoptotic ECs (cDcp-1^+^ ECs, cDcp-1^+^ apoptotic bodies and pyknotic EC nuclei) on the basal side of the epithelium and near progenitors, we did not detect cDcp-1^+^ corpses (Fig. 5A) nor condensed nuclei within progenitors. These data indicate that midgut progenitors do not clear apoptotic ECs. Since we observed cDcp-1 in the midgut epithelium after *P.e*. infection, we tested whether progenitors cleared apoptotic ECs in infected midguts. To do this, we expressed GFP in ECs with *mex*^ts^ and infected these flies with *P.e*.. We then determined whether GFP from apoptotic ECs could be detected within Arm^high+^ progenitors. Similar to EC ablation, we did not detect GFP^+^ corpses within progenitors (Figs. 5B and EV5A–B”). These data suggest that Draper signalling in progenitors does not participate in phagocytosis and apoptotic cell clearance after midgut damage. This finding is consistent with our data indicating that apoptotic ECs are eliminated into the lumen (Fig. 1D).

Since we did not detect corpses within midgut progenitors, we tested whether EC corpses were rapidly degraded after engulfment by progenitors via lysosomal degradation. ECs were marked with nuclear ß-galactosidase (ß-gal) using *Myo1A-lacZ*. We infected flies carrying *Myo1A-lacZ* with *P.e*. and determined if ß-gal from dying ECs could be detected within midgut progenitors expressing a dominant-negative Rab7 (T22N), which would prevent the degradation of phagosome contents by blocking fusion of the late endosome with lysosomes (Han et al, 2014; Melcarne et al, 2019). We found stable nuclear ß-gal expression in ECs after *P.e*. infection (Fig. EV5C–D’). However, we did not find nuclear ß-gal in Arm^high+^ Pros^-^ progenitors, indicating that *Myo1A* expression is not induced in progenitors after midgut damage (Fig. EV5C–F’,G). We then determined whether ß-gal from neighbouring ECs can be detected within the cytoplasm of Arm^high+^ Pros^−^ progenitors in uninfected and *P.e*.-infected control midguts. We found that 6.9% (mean) of progenitors within infected midguts were positive for ß-gal in their cytoplasm (Fig. EV5D,D’,H). This was similar to progenitors within uninfected control midguts (4.9%, mean) (Fig. EV5C,C’,H), suggesting that dying ECs are not cleared by progenitors. In dominant-negative Rab7 expressing progenitors, we similarly found that 6.2% (mean) of progenitors within infected midguts were positive for ß-gal in their cytoplasm (Fig. EV5F,F’,H). This was similar to progenitors expressing dominant-negative Rab7 in uninfected midguts (3.8%, mean) (Fig. EV5E,E’,H) and to progenitors in control infected midguts.

Together, these data further support our conclusion that progenitors use Draper-Src-Shark signalling to recognise apoptotic damaged cells but not to clear them.

Draper-Src-Shark signalling was found to activate STAT activity via Rac1 within glia phagocytosing degenerating axonal debris of ORNs (Doherty et al, 2014). These data suggest that engulfment is possibly required for STAT activation in glia after ORN injury since Rac is important for phagocytic cup formation (Mao and Finnemann, 2015). To test whether Rac1 signalling is required to activate STAT in midgut progenitors, we blocked Rac1 signalling and thus also Rac-mediated engulfment in progenitors by expressing a dominant-negative Rac (N17)(Doherty et al, 2014; Etchegaray et al, 2012; Mishra et al, 2023; Torres et al, 2023) and examined STAT activity in these progenitors after *P.e*. infection. We found that progenitors expressing dominant-negative Rac induced STAT activity after *P.e*. infection similar to progenitors in control infected midguts (Figs. 5C and EV5I–L). These data suggest that STAT activation in progenitors after midgut damage does not require Rac signalling nor Rac-mediated engulfment.

In summary, we have here uncovered the mechanism by which *Drosophila* midgut progenitors recognise damage and translate this into increased STAT activity within progenitors and the visceral muscle to ensure regeneration (Fig. 5D–E’). Together, Draper-Src-Shark signalling in progenitors stimulates STAT activity within ISCs and EBs. Moreover, Draper-Src-Shark signalling in progenitors activates STAT in the visceral muscle, indicating that progenitors can shape the regenerative microenvironment after tissue damage. Collectively, the increase of STAT activity within ISCs and the visceral muscle boosts ISC proliferation to promote regeneration.

## Discussion

While it is well understood how signalling cues from the regenerative microenvironment stimulate ISC proliferation after damage to the adult *Drosophila* midgut, little is known about how ISCs sense damage and whether this modifies ISC behaviours such as proliferation.

Apoptosis is observed in the midgut epithelium after midgut damage due to pathogenic bacterial infection and detergent damage (Buchon et al, 2009b; Loudhaief et al, 2017; Petsakou et al, 2023). Furthermore, inhibiting apoptosis in ECs with p35 expression suppresses ISC proliferation after DNA damage (Amcheslavsky et al, 2009). We similarly found increased cleaved Dcp-1-positive apoptotic cells in the midgut epithelium after *P.e*. infection (Fig. 1E,F). Moreover, we found that inhibiting apoptosis in ECs by expressing DIAP1 or p35, inhibited ISC proliferation after *P.e*. infection (Figs. 1L and EV1I). These data suggest that EC apoptosis in damaged midguts is required for ISC proliferation.

The ablation of ECs by expressing the pro-apoptotic factor, Reaper, in the adult *Drosophila* midgut epithelium induces ISC proliferation to promote regeneration (Jiang et al, 2009). It is thought that ISC proliferation after EC apoptosis is largely due to increased cytokine (Upd1-3) expression in the midgut epithelium, which stimulates STAT activation in ISCs and thus, their proliferation (Jiang et al, 2009). Nevertheless, an early event of apoptosis is the relocalisation of phosphatidylserine to the extracellular side of the plasma membrane (Melcarne et al, 2019; Nagata et al, 2016). Phosphatidylserine is normally found on the intracellular side of the plasma membrane within living cells. Interestingly, we found that Draper, a *Drosophila* immunoreceptor that recognises phosphatidylserine (Ji et al, 2023; Melcarne et al, 2019; Tung et al, 2013), is highly expressed on adult midgut progenitors (Fig. 1G,G’). Thus, we hypothesised that ISCs may recognise phosphatidylserine as a damage signal on dying enterocytes, triggering their proliferation. Consistent with this, we found that masking phosphatidylserine exposed on apoptotic ECs or on ECs in *P.e.-*infected midguts inhibited ISC proliferation (Fig. 1M,N).

Src family kinases phosphorylate tyrosines within ITAM domains of immune receptors at the plasma membrane, enabling Syk kinases to bind these ITAMs through their SH2 domains (Mansueto et al, 2019; Mocsai et al, 2010). SYK binding to ITAMs allows for its full activation by further phosphorylation by Src family kinases (Mansueto et al, 2019; Mocsai et al, 2010). Indeed, we found activated Src42A to accumulate cortically in progenitors after *P.e*. infection (Fig. 1H–J), likely allowing Src42A to phosphorylate the ITAM-containing immunoreceptor, Draper, and the SYK-homologue, Shark. In support, we found that Draper, Src42A and Shark are required in ISCs to stimulate their proliferation upon midgut damage (Fig. 2E–G).

Phosphatidylserine-bound Draper forms microclusters in the plasma membrane, which exclude an inhibitory transmembrane phosphatase, thereby allowing it to remain phosphorylated and activated by Src42A (Williamson and Vale, 2018) (Fig. 1O). In midgut progenitors, the increase in activated Src42A levels by Wg and Ret signalling after damage likely leads to net Draper activation and phosphorylation (Cordero et al, 2012; Perea et al, 2017; Williamson and Vale, 2018) and sustains Draper signalling throughout regeneration. Phosphorylation of the Draper ITAM by Src42A enables Shark binding to Draper and its further activation by Src42A (Tung et al, 2013; Williamson and Vale, 2018; Ziegenfuss et al, 2008). We found that both Draper and Shark are partially required for constitutively active Src42A to promote ISC proliferation (Fig. 2I,J). Interestingly, the requirement for Draper for constitutively active Src42A signalling to promote ISC proliferation increased after *P.e*. infection (Fig. 2I), which may be largely through Draper-Shark signalling. This could be due to infection-induced EC apoptosis and Draper binding to exposed phosphatidylserine on ECs, thereby facilitating the phosphorylation of its ITAM by constitutively active Src42A. We also found that EC apoptosis can increase activated Src levels in progenitors (Figs. 1K and EV1F–H)). However, we found that whilst EC apoptosis strongly induces ISC proliferation through both Draper and Src42A (Fig. 2A,B), it did not strongly increase the levels of activated Src in progenitors (Figs. 1K and EV1F–H). This suggests that even mild increases in activated Src are sufficient to drive ISC proliferation after midgut damage. The increase in activated Src levels after midgut damage might ensure continued Draper signalling in progenitors and/or promote other pro-regenerative ISC behaviours.

We found that Src42A signalling is required in progenitors to respond to both pathogenic bacterial infection (*P.e*.) and chemical stress (SDS) (Figs. 2D and EV2D,E), however, it was not clear whether Src activity was consequential in ISCs or EBs to induce ISC proliferation after tissue damage. The progenitor population consists of three cell types, ISCs, EBs and EEps. We found that Src42A signalling is required substantially more in ISCs than in Su(H)^+^ EBs to promote ISC proliferation after midgut damage (Fig. 2F,H). Whilst Wg and Ret signalling have been shown to promote ISC proliferation via Src activation in progenitors (Cordero et al, 2014; Perea et al, 2017), our work pinpoints the importance of Src42A activity in ISCs for their proliferative response to midgut damage.

Draper is known to detect dying cells through its binding to phosphatidylserine (Ji et al, 2023; Melcarne et al, 2019; Tung et al, 2013). Draper signalling is utilised by non-professional phagocytes (glia, epithelial cells) to recognise and clear dying cells and debris (Etchegaray et al, 2012; Han et al, 2014; MacDonald et al, 2006; Melcarne et al, 2019). Interestingly, zebrafish epithelial stem cells and mouse hair follicle stem cells engulf apoptotic corpses (Brock et al, 2019; Stewart et al, 2024). Furthermore, the engulfment of dying basal stem cells in the zebrafish epidermis by neighbouring stem cells promotes their proliferation to maintain tissue homeostasis (Brock et al, 2019). We indeed found that EC apoptosis after *P.e*. infection is required for ISC proliferation (Figs. 1L and EV1I). Moreover, we found that masking externalised phosphatidylserine on ECs blocked ISC proliferation after EC ablation and *P.e*. infection (Fig. 1M,N). Additionally, we found that Draper is required in ISCs for their proliferation after EC ablation and *P.e.-*induced damage (Fig. 2A,E), suggesting that Draper on ISCs may recognise apoptotic midgut epithelial cells through phosphatidylserine recognition, clear them through phagocytosis and promote ISC proliferation. Nevertheless, we did not detect the clearance of apoptotic EC corpses by progenitors either following EC ablation or during *P.e*. infection (Figs. 5A,B and EV5A–B”). This could be due to the much larger size and number of apoptotic ECs compared to ISCs or because apoptotic cells in the midgut extrude apically into the lumen (Buchon et al, 2010; Kolahgar et al, 2015) (Fig. 1A,D). Engulfment would require progenitors to enlarge or stretch to internalise dying cells or debris, which we did not observe. Moreover, apoptotic cell clearance by progenitors would be less efficient than eliminating them directly into the lumen as there are far fewer ISCs (∼10%) than ECs (∼70%). Indeed, we found that after EC ablation, apoptotic ECs can be found in the midgut epithelium and within the lumen (Fig. 1D). Since we found the number of apoptotic ECs to increase progressively from the basal side of the epithelium towards the lumen, apoptotic cells are likely cleared into the lumen rather than by progenitors in the adult *Drosophila* midgut. In other tissues where Draper has been found to facilitate the clearance of dying cells, such as the developing embryo or larva, adult olfactory system, testis or ovary (Etchegaray et al, 2012; Han et al, 2014; Kurant et al, 2008; MacDonald et al, 2006; Zohar-Fux et al, 2022), there is no external space into which apoptotic cells can be easily eliminated. Thus, local non-professional phagocytes must clear apoptotic corpses in these tissues. While we did not detect midgut progenitors clearing apoptotic ECs even after blocking lysosomal degradation (Fig. EV5C–H), it is still possible that engulfed EC corpses were rapidly degraded. Furthermore, our data do not rule out that progenitors engulf fragments of dying neighbouring ECs.

We therefore concluded that Draper on ISCs senses apoptotic midgut epithelial cells but does not clear them from the epithelium. The Draper homologue in humans is also required in muscle satellite cells for skeletal muscle regeneration (Li et al, 2021); however, how MEGF10 promotes regeneration is not understood. It would be intriguing to examine whether MEGF10 acts as a sensor for apoptotic cells on various stem cells after tissue injury, suggesting a conserved mechanism.

Nonetheless, it was still unclear how Draper-Src-Shark signalling in ISCs activates ISC proliferation. Interestingly, STAT is activated by Draper-Src42A-Shark-Rac1 signalling in glial cells after injury to adult *Drosophila* ORN axons (Doherty et al, 2014). Furthermore, STAT is required for ISC proliferation after pathogenic bacterial infection (Buchon et al, 2009a; Jiang et al, 2009). Moreover, STAT is activated by cytokines Upd1-3 provided by the regenerative microenvironment after midgut damage (Herrera and Bach, 2019; Zhang and Edgar, 2022; Zhou and Boutros, 2023). Upon pathogenic bacterial infection, Upd1 is produced by progenitors and early ECs; Upd2, by EBs and ECs and Upd3, by ECs and Malpighian tubules (Jiang et al, 2011; Jiang et al, 2009; Liu et al, 2024; Osman et al, 2012; Zhou et al, 2013). The importance of these cytokines for ISC proliferation after midgut damage is demonstrated by the near-complete abolition of ISC proliferation in *upd2* and *upd3* double mutants after pathogenic bacterial infection (Ahmed et al, 2020; Osman et al, 2012). While these data suggest a strong activation of STAT by cytokines provided by the regenerative microenvironment, we found that Draper-Src42A-Shark signalling is required and sufficient for STAT activation in progenitors after *P.e*. infection (Figs. 3,  EV3, and EV4G–I). Whilst Rac is required for STAT activation in glial cells after injury to adult ORN axons (Doherty et al, 2014), STAT activation in midgut progenitors by Draper-Src-Shark signalling did not require Rac activity and thus, Rac-mediated engulfment (Fig. 5C). Furthermore, these data suggest a role for Draper-Src-Shark signalling to regulate STAT activity in EBs. Moreover, Draper-Src-Shark signalling is required and sufficient in progenitors to activate STAT in the VM (Figs. 4A–J and EV4A–F’,J,K). This suggests that ISCs, EBs or both ISCs and EBs can non-autonomously regulate the regenerative microenvironment after midgut damage, possibly as a fail-safe mechanism. Indeed, in the VM, elevated STAT activity following infection has been shown to induce EGF (Vein) production, thereby stimulating ISC proliferation (Jiang et al, 2011; Zhou et al, 2013).

Yet how Draper-Src-Shark signalling stimulates STAT activity in adult midgut progenitors and the VM remained an open question. Previously, it was reported that cytokine-Domeless signalling is not required for constitutively active Src42A to promote ISC proliferation (Cordero et al, 2014; Kohlmaier et al, 2015). However, we found that simultaneous depletion of *domeless* and the expression of constitutively active Src42A in progenitors inhibited ISC proliferation (Fig. 4K), suggesting Draper-Src-Shark signalling within progenitors requires cytokine-Domeless signalling for STAT activation in progenitors and in the VM. Because constitutively active Src42A signalling strongly requires Draper in progenitors to promote ISC proliferation after infection (Fig. 2I), it suggests that Domeless acts downstream of Draper-Src-Shark signalling. Additionally, we found that Draper was not required in progenitors for cytokine *upd1-3* expression in the midgut epithelium after *P.e*. infection (Fig. 4L), indicating that progenitors lacking Draper signalling fail to activate STAT despite robust *upd1-3* expression in the midgut epithelium. Not much is known about Upd1-3 regulation after their transcription in damaged midguts. Both Upd1 and Upd2 are expressed by progenitors after midgut damage (Jiang et al, 2009; Osman et al, 2012; Tian et al, 2015). Thus, one possibility is that Draper signalling in progenitors regulates Upd1 and Upd2 production or secretion, thus activating STAT in both progenitors and in the VM. Alternatively, Draper signalling could autonomously influence progenitors and non-autonomously the regenerative microenvironment by modulating cytokine (Upd1-3) activity, thereby regulating STAT in both progenitors and the VM. Draper signalling in progenitors could facilitate proper cytokine Upd1-3 production or secretion by midgut epithelial cells or regulate their stability or localisation. Since Upd1 may be the most potent of all three cytokines (Osman et al, 2012; Wright et al, 2011), this may allow local regulation of STAT activity by progenitors, both autonomously and non-autonomously in the VM. Future studies will be needed to determine how progenitors permit cytokine-Domeless signalling in progenitors and in the VM.

How STAT3 is activated in the mouse intestinal epithelium after damage is not fully understood. Similar to *Drosophila*, STAT3 is thought to be largely activated by cytokines—IL-6 and IL-10 are produced by macrophages, and IL-22 is produced by type-3 innate lymphoid (ILC3) cells (Hageman et al, 2020; Palikuqi et al, 2022). Nevertheless, STAT3 may also be partly regulated by ISC intrinsic factors that sense intestinal damage. This is supported by the fact that Src-STAT3 signalling in the crypt containing ISCs and transit amplifying progenitors is critical for mouse intestinal epithelial regeneration after DNA damage by γ-irradiation (Cordero et al, 2014). Furthermore, Src is required for STAT3 activation after γ-irradiation, indicating that epithelial Src activity is crucial for STAT3 activation after injury (Cordero et al, 2014). Nevertheless, it is not clear how Src regulates STAT3 after injury. Whether Src in intestinal progenitors promotes damage sensing and activates STAT3 through MEGF10 or SYK or by modulating cytokine signalling as in our model remains to be determined.

Src is also activated in non-invasive human adenomas (Cordero et al, 2014). Thus, it would be interesting to examine whether stem-like tumour cells utilise Src signalling to detect apoptotic cells within tumours or normal neighbouring tissue to fuel their growth. Alternatively, stem-like tumour cells could utilise Src signalling to detect apoptotic cells after chemotherapy or radiation treatment to promote tumour regrowth. In summary, our work increases our understanding of how stem cells recognise damage, couple it to their proliferation and influence their surrounding microenvironment to support tissue regeneration. We hope that this knowledge will aid in the development of treatments for epithelial regeneration after injury, pathogenic infection and damage caused by inflammatory disease and cancer therapies.

## Methods

**Table:** Reagent and tools table

Reagent/resource	Reference or source	Identifier or catalogue number
**Experimental models**		
*w* ^*1118*^	Jiang et al, 2009	
*draper*^*Δ5*^ (rec 9)	Marc Freeman (Oregon Health and Science University); MacDonald et al, 2006	
*yw; Src42A* ^*K10108*^ */CyO*	Bloomington *Drosophila* Stock Center; Cordero et al, 2014	10969
*Stat92E* ^*397*^	Jiang et al, 2009	
*Myo1A-lacZ*	Jiang et al, 2009	
*w*; *10X STAT-dGFP*	Bach et al, 2007; Jiang et al, 2009	
w; *esg-GAL4; tubGAL80*^ts^ *UAS-GFP* (*esg*^ts^*GFP*)	Jiang et al, 2009	
*w; esg-GAL4 UAS-mRFP; tubGAL80*^ts^ (*esg*^ts^*mRFP*)	Tamamouna et al, 2021	
*esg-GAL4 UAS-2X EYFP; Su(H)Gbe-GAL80, tubGAL80*^ts^ (*esg*^ts^ *Su(H)-GAL80*)	Wang et al, 2014	
*w; Su(H)Gbe-GAL4; tubGAL80*^ts^ *UAS-GFP* (*SuH*^ts^)	Zeng et al, 2010	
*mex-GAL4; tubGAL80*^ts^ (*mex*^ts^)	Irene Miguel-Aliaga (The Francis Crick Institute); Phillips and Thomas, 2006	
*UAS-DIAP*	Jiang et al, 2009	
*yv*; *UAS-GFP*	Bloomington *Drosophila* Stock Center	35786
UAS-milk fat globule EGF-8	Estee Kurant (University of Haifa) Tung et al, 2013	
*UAS-p35*	Jiang et al, 2009	
*yw; UASp-YFP-Rab7.T22N*	Bloomington *Drosophila* Stock Center	9778
*yw; UAS-Rac1.N17*	Bloomington *Drosophila* Stock Center; Doherty et al, 2014; Etchegaray et al, 2012; Mishra et al, 2023; Torres et al, 2023	6292
*w; UAS-reaper* (1)	Jiang et al, 2009	
*w, UAS-reaper* (2)	Bloomington *Drosophila* Stock Center	5823
*w; UAS-Src42A* ^*CA*^	Bloomington *Drosophila* Stock Center	6410
*w; UAS-domeless* ^*RNAi*^	Vienna *Drosophila* Resource Center	106071
*w; UAS-draper*^*RNAi*^ (1)	Vienna *Drosophila* Resource Center; Han et al, 2014	4833GD
*w; UAS-draper*^*RNAi*^ (2)	Vienna *Drosophila* Resource Center; Han et al, 2014	27086GD
*yv*; *UAS-mCherry*^*RNAi*^	Bloomington *Drosophila* Stock Center	35785
*yv; UAS-pretaporter* ^*RNAi*^	Bloomington *Drosophila* Stock Center	HMC04406
*yv; UAS-shark*^*RNAi*^ (1)	Bloomington *Drosophila* Stock Center; Torres et al, 2023	HMC04146
*w; UAS-shark*^*RNAi*^ (2)	Vienna *Drosophila* Resource Center; Doherty et al, 2014	105706KK
*yv; UAS-Src42A*^*RNAi*^ (1)	Bloomington *Drosophila* Stock Center	HMC04138
*UAS-Src42A*^*RNAi*^ (2)	Vienna *Drosophila* Resource Center; Doherty et al, 2014	26019GD
**Antibodies**		
Mouse anti-Armadillo (1:50)	Developmental Studies Hybridoma Bank (DHSB)	N2 7A1
Chicken anti-ß-galactosidase (1:1000)	Abcam	ab9361
Rabbit anti-cleaved-Dcp-1 (Asp215) (1:100)	Cell Signaling Technology	9578
Mouse anti-Draper (1:500)	Developmental Studies Hybridoma Bank (DHSB)	8A1
Chicken polyclonal anti-GFP (1:1000)	Life Technologies	A10262
Rabbit polyclonal anti-phospho-Histone H3 (Ser 10) (1:1000)	Merck	06-570
Mouse monoclonal anti-phospho-Histone H3 (Ser 10)(3H10) (1:1000)	Sigma-Aldrich	05-806
Rabbit polyclonal anti-phosphorylated human Src Y419 (1:100)	Thermo Fisher Scientific/Invitrogen; Cordero et al, 2014; Perea et al, 2017	44-660 G
Rabbit polyclonal anti-phosphorylated Src Y416 (1:10)	Cell Signaling Technology; Torres et al, 2023	2101S
Mouse anti-Prospero (1:50)	Developmental Studies Hybridoma Bank (DHSB)	MR1A
Rabbit polyclonal anti-RFP (1:1000)	Thermo Fisher Scientific	R10367
Alexa Fluor 488 anti-chicken (1:1000)	Thermo Fisher Scientific	A-11039
Alexa Fluor 568 anti-rabbit (1:1000)	Thermo Fisher Scientific	A-11036
Alexa Fluor 568 anti-mouse (1:1000)	Thermo Fisher Scientific	A-11031
Alexa Fluor 633 anti-rabbit (1:1000)	Thermo Fisher Scientific	A-21071
Alexa Fluor 633 anti-mouse (1:1000)	Thermo Fisher Scientific	A-21052
**Oligonucleotides and other sequence-based reagents**		
*upd* forward primer:	Thermo Fisher Scientific; Chakrabarti et al, 2016	AATCAGCTGAAGCGCCACG
*upd* reverse primer:	Thermo Fisher Scientific; Chakrabarti et al, 2016	GGAATTGGGCTTGAGCTTGG
*upd2* forward primer:	Thermo Fisher Scientific; Chakrabarti et al, 2016	GGCTCTTCTGCTGATCCTTG
*upd2* reverse primer:	Thermo Fisher Scientific; Chakrabarti et al, 2016	AAGACTTGGTACCGCCACAT
*upd3* forward primer:	Thermo Fisher Scientific; Chakrabarti et al, 2016	GCGGGGAGGATGTACC
*upd3* reverse primer:	Thermo Fisher Scientific; Chakrabarti et al, 2016	GTCTTCATGGAATGAGCC
*crq* forward primer:	Thermo Fisher Scientific; Patel et al, 2019	CAGAGCTCTCCTCCGAATTG
*crq* reverse primer:	Thermo Fisher Scientific; Patel et al, 2019	ATGCCGGTGATGAGAAAGAC
**Chemicals, enzymes and other reagents**		
Hoechst 33258	Thermo Fisher Scientific	H1399
Vectashield	Vector Laboratories	H-1000-20
PhosSTOP	Merck Life Science Limited	4906837001
Arcturus PicoPure RNA Isolation Kit	Thermo Fisher Scientific /Applied Biosystems	12204-01
DNAse	Qiagen	79254
SuperScript IV First-Strand Synthesis System	Thermo Fisher Scientific/Invitrogen	18091050
QuantiNova SYBR Green PCR Kit	Qiagen	208054
**Software**		
ImageJ	National Institutes of Health	
Prism 10	Graphpad	
Adobe Photoshop	Adobe	
Adobe Illustrator	Adobe	

### *Drosophila melanogaster* housing and husbandry

*Drosophila melanogaster* were housed at either 18 °C or 25 °C (65% humidity) under a light-dark cycle and fed a *Drosophila* medium containing water, agar, glucose, dry yeast, wheat flour, 10% Nipagin and propionic acid. The stocks used in this study can be found in the Reagents and Tools table.

### *Drosophila* genetics

Flies raised at 18 °C were shifted to 29 °C to induce UAS transgene expression. Experiments were performed using 5–10 day old, adult, virgin females except for experiments in Figs. 1H–J, 2F,  EV1A–E and  EV2B,E,H, which were performed using mated females. Typically, between 10 and 20 flies were used per experiment. Flies were selected by their genotype and then randomly chosen to be used in an experiment.

### *P.e.* culture and preparation for oral infection

*Pseudomonas entomophila* (*P.e.*) was grown by inoculating 3 ml of Luria Broth (LB) with 100 μg/ml rifampicin with a single colony of *P.e*. grown on an LB-rifampicin plate. The 3 ml culture was grown for 24 h at 30 °C at 130 RPM. In total, 500 μl of this culture was diluted into 50 ml of fresh LB with rifampicin, which was then grown for 18 h at 30 °C at 130 RPM. The bacteria were then centrifuged at 3000×*g* for 30 min. The bacterial pellet was then resuspended in 2 ml of 5% sucrose and diluted in 5% sucrose to CFU = 7.0 × 10^9^ bacteria/ml.

### Stress experiments

*P.e*. infection: flies were treated for 18 h with food laced with 500 μl of 5% sucrose or *P.e*. resuspended in 5% sucrose (CFU = 7.0 × 10^9^ bacteria/ml) from an 18 h culture grown at 30 °C, 130 RPM.

Detergent exposure: 500 μl of 0.2% sodium dodecyl sulfate (SDS) or H_2_O was mixed into the food.

### Histology

Following fixation in 8% formaldehyde for 30–60 min, midguts were washed in PBS, 0.1% Triton X-100, blocked in PBS, 0.1% Triton X-100, 1% bovine serum albumin (BSA), 2% normal goat serum (NGS), and stained in blocking solution with primary antibodies found in the Reagents and Tools table. Midguts were then washed in PBS 0.1% Triton X-100, stained in PBS, 0.3% Triton X-100, 0.1% BSA with Alexa Fluor-conjugated secondary antibodies and Hoechst 33258 and mounted in Vectashield. For anti-phosphorylated-Src, midguts were fixed in 8% formaldehyde containing PhosSTOP. Experiments in Figs. 1B–F, 5A,B and  EV5A–B” were initially fixed with 4% formaldehyde and then with ethanol/methanol as described below. For anti-cleaved-Dcp-1 staining, midguts were first fixed in 400 μL of 4% formaldehyde for 1 h, 200 μL 100% methanol was slowly added to the formaldehyde and left for 5 min. Midguts were then transferred into 400 μL of 100% ethanol for 10 min. 300 μL of PBS was added slowly to the ethanol before transferring the midguts into PBS for 15 min. Midguts were then washed twice in PBS, 0.1% Triton X-100 for 30 min prior to staining with anti-cleaved Dcp1.

### Microscopy

Samples were analysed using a Leica DMI6000 epifluorescence microscope and a Leica SP5-II confocal laser scanning microscope. Images were processed with ImageJ (NIH) and Adobe Photoshop. Confocal images are presented as maximum intensity projections or as single slices. A z-stack for each midgut within an experiment was acquired with the same laser intensity and gain. Samples were blinded before image acquisition and or quantitation of mitoses per midgut.

The number of mitoses per midgut was determined by counting the number of phospho-Ser 10 histone 3-positive cells from whole female midguts. The mean mitoses per midgut and 95% confidence interval are presented for each genotype and treatment.

### Image quantitation

*Phosphorylated-Src in progenitors:* average intensity projections of 12 slices from 0.25 μm z-stacks of the basal layer containing progenitors were generated for the *esg* > GFP^+^ and Armadillo (Arm) channels and merged using ImageJ. Similarly, average intensity projections of 12 slices were generated for the pSrc channel using ImageJ, and rolling ball (10 pixel radius) background subtraction was applied prior to quantitation. *esg* > GFP^+^ cells for each condition were randomly selected, and a line (thickness of 3) was drawn along the cortical Arm signal near the inner side of the plasma membrane using the ImageJ freehand selection tool. These selections were restored onto pSrc projections, and the raw integrated density was measured along the line. The raw integrated density was then multiplied by 12 and divided by the line length to obtain the normalised fluorescence intensity of pSrc at the membrane per cell. Single slices were used to quantify pSrc in Fig. 1K.

*Phosphorylated-Src in the midgut epithelium:* sum intensity projections of 6 slices from 1 μm z-stacks of the progenitor layer for the pSrc channel were generated using ImageJ. The freehand selection tool was used to trace around the epithelium to measure the raw integrated density and epithelial area. The raw integrated density of pSrc was then divided by the area of the selected epithelium to obtain the normalised fluorescence intensity of pSrc in the whole epithelium.

*STAT-GFP in progenitors:* sum projections of ten slices from 0.5 μm z-stacks of the basal layer containing progenitors (high Arm^+^) were generated for the Arm and *10X STAT-dGFP* channels using ImageJ. The Arm signal within high Arm^+^ cells was randomly selected and traced using the freehand selection tool, and the selection was restored onto *10X STAT-dGFP* projections to measure the raw integrated density within the cell. The raw integrated density for each progenitor was then divided by the area of each progenitor to determine the normalised fluorescence intensity of 10× STAT-dGFP per cell.

*STAT-GFP in visceral muscle:* sum projections were generated from 15 slices of 0.13 μm z-stacks for the DNA (Hoechst) and *10X STAT-dGFP* channels using ImageJ. The circular visceral muscle nuclei were randomly selected and traced using the freehand selection tool. These selections were then restored onto the *10X STAT-dGFP* channel to measure the raw integrated density for *10X STAT-dGFP* within nuclei. The raw integrated density for each nucleus was then divided by the area of each nucleus to determine the normalised fluorescence intensity of *10X STAT-dGFP* per nucleus.

*Distribution of apoptotic enterocytes:* 3 single slices were chosen from each midgut, one from the basal side of the epithelium, one from the apical side of the epithelium and one from the lumen. All apoptotic enterocytes (cDcp-1^+^ ECs, cDcp-1^+^ bodies and pyknotic nuclei) were then manually counted from each slice.

*Cleaved-Dcp-1 in progenitors:* The Arm and cleaved-Dcp-1 channels were merged in ImageJ. 30 single slices containing Arm^high+^ progenitors were individually examined from the progenitor layer of 0.5 μm z-stacks. All Arm^high+^ progenitors were scored every ten slices for the presence of cleaved-Dcp-1. Approximately 30–70 cells were examined per midgut.

*GFP*^*+*^ *corpses in progenitors*: The Arm and GFP channels were merged in ImageJ. 30 single slices containing Arm^high+^ progenitors were individually examined from the progenitor layer of 0.5 μm z-stacks. All Arm^high+^ progenitors were scored every ten slices for the presence of GFP. Approximately 60-100 cells were examined per midgut.

*Nuclear and cytoplasmic β-gal in progenitors:* The Arm and β-gal channels were merged in ImageJ. In all, 30 single slices containing Arm^high+^ progenitors were individually examined from the progenitor layer of 0.5 μm z-stacks. All Arm^high+^ progenitors were scored every 10 slices for nuclear β-gal expression or for the presence of cytoplasmic β-gal. Approximately 70–120 cells were examined per midgut.

*esg*^*+*^ *progenitor cell /total cell ratio:* maximal intensity projections of 10 slices of 1 μm z-stacks were generated for the esg>GFP and DNA (Hoechst) channels in ImageJ. The total number of *esg*^*+*^ cells with small nuclei and the total number of nuclei were then counted. The number of *esg*^+^ cells was then divided by the total number of nuclei to obtain the ratio *esg*^*+*^ cells/ total cells.

### Quantitative RT-PCR

RNA was isolated from 20 midguts using the PicoPure RNA extraction kit and DNase-treated during RNA isolation using the Qiagen RNAse-free DNase Set. The RNA concentration was determined using a NanoDrop ND-1000 spectrophotometer. 250 ng of RNA was used for cDNA synthesis using the Superscript IV First-Strand Synthesis System. qPCR was performed using QuantStudio 5 from Applied Biosystems. Forward and reverse primers used are noted in the Reagents and Tools Table. Crq was used as a reference, which did not change in expression with *P.e*. infection. A standard curve was performed to calculate primer efficiencies, which were between 90 and 110% for all primers. Each assay was performed in triplicate on 3 independent biological replicates. Fold changes were determined using the delta-delta C_t_ method.

### Statistical analysis

Statistical analyses were performed using Graphpad Prism 10. A two-sided unpaired *t* test or Mann–Whitney test (for non-Gaussian data) were applied to determine statistical significance.

## Supplementary information

Appendix

Peer Review File

Figure EV2 Source Data

Source data Fig. 1

Source data Fig. 2

Source data Fig. 3

Source data Fig. 4

Source data Fig. 5

Figure EV1 Source Data

Figure EV3 Source Data

Figure EV4 Source Data

Figure EV5 Source Data

Expanded View Figures

## Data Availability

This study includes no data deposited in external repositories. All data are provided within the Figures, Expanded View figures, the Appendix and the Source Data, which includes a file with numerical data. The source data of this paper are collected in the following database record: biostudies:S-SCDT-10_1038-S44318-026-00890-1.
