# Supplementary material for: Damage recognition by intestinal stem cells via Draper promotes adult Drosophila midgut regeneration
Source: EMBO J. 2026 Aug 5;45(18):6450–79. doi: 10.1038/s44318-026-00890-1 (PMC13578246; doi:10.1038/s44318-026-00890-1)
Supplement: Supplementary file 1 [file 44318_2026_890_MOESM13_ESM.pdf]

Expanded View Figures

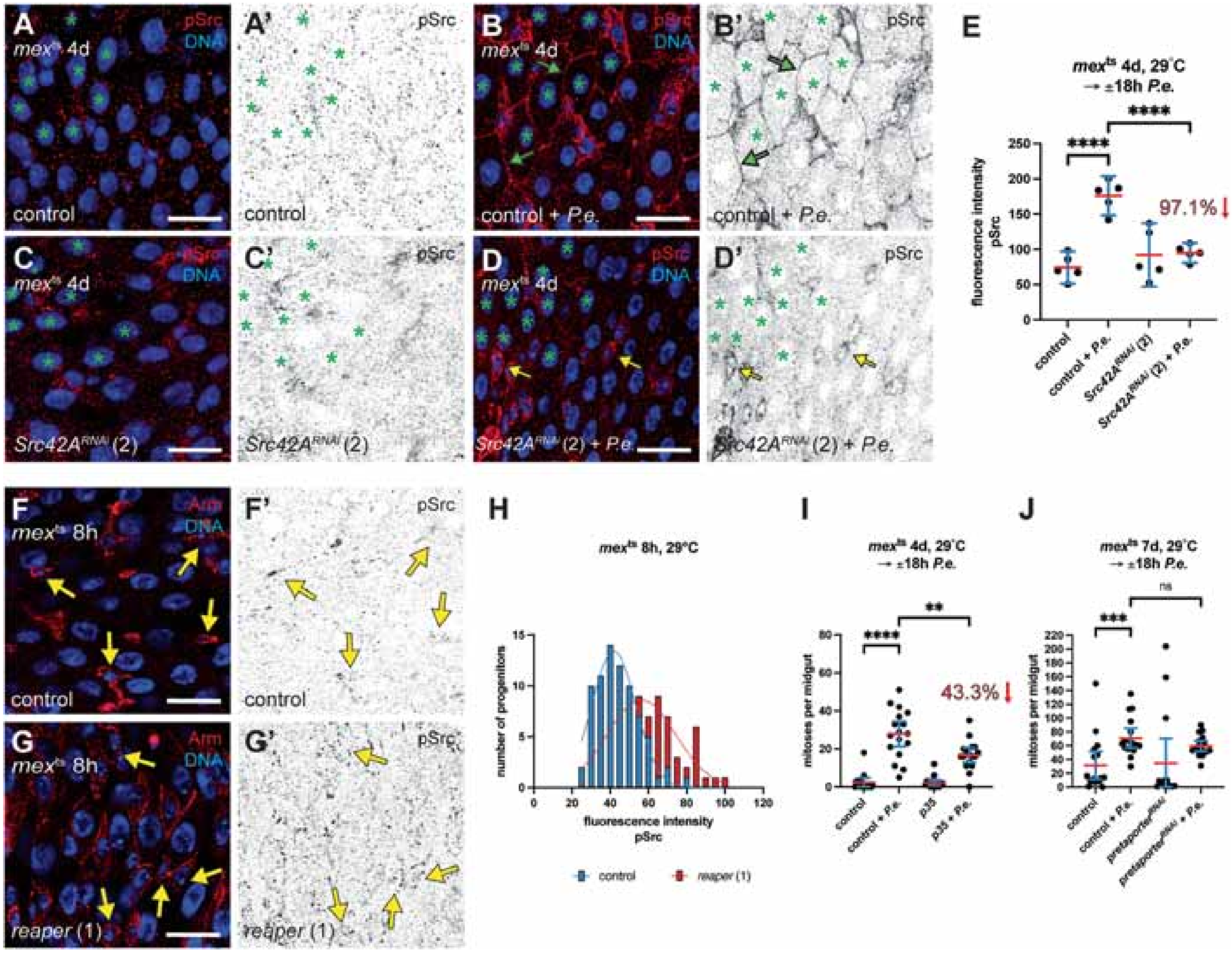

Figure EV1. Midgut damage and enterocyte apoptosis activate Src in progenitors.

Anti-phosphorylated human Src Y419 (pSrc) antibody detects Src42A activation (**A–E**). Levels of pSrc (**A**, **B**; red) increased on the lateral membranes (green arrows) of enterocytes (ECs, green asterisks) and cortically within progenitors after *P.e.* infection (**B–B′**) compared to uninfected midguts (**A–A′**). ECs expressing *Src42A*^{RNAi} had decreased pSrc in their lateral membranes after *P.e.* infection compared to control *P.e.*-infected midguts (**D–D′**, **B–B′**). Note in (**D–D′**), progenitors (yellow arrows) still cortically accumulated pSrc after *P.e.* infection (**D–D′**). Mean pSrc levels increased in the epithelium of *P.e.*-infected midguts ($n = 5$ midguts from one experiment) compared to uninfected control midguts ($n = 5$ midguts from one experiment) (unpaired *t* test; $P < 0.0001$). Mean cortical pSrc levels decreased in the epithelium of *P.e.*-infected midguts expressing *Src42A*^{RNAi} in ECs ($n = 5$ midguts from one experiment) compared to *P.e.*-infected control midguts (unpaired *t* test; $P < 0.0001$) (**E**). Apoptosis increases cortical pSrc accumulation in progenitors (**F–H**). Levels of pSrc (**F′**, **G′**) increased cortically within progenitors (yellow arrows) after *reaper* expression in ECs (**G′**) compared to control midguts (**F′**). Mean cortical pSrc levels increased in progenitors of midguts expressing *reaper* in ECs ($n = 75$ cells from five midguts from one of two experiments) (**H**, red) compared to control midguts ($n = 75$ cells from five midguts from one of two experiments) (**H**, blue). Blocking apoptosis in ECs inhibits ISC proliferation after *P.e.* infection (**I**). The mean mitoses per midgut increased in *P.e.*-infected control midguts ($n = 17$ midguts from one experiment) compared to uninfected control midguts ($n = 16$ midguts from one experiment) (Mann–Whitney; $P < 0.0001$). The mean mitoses per midgut decreased in *P.e.*-infected midguts expressing p35 in ECs ($n = 17$ midguts from one experiment) compared to *P.e.*-infected control midguts (Mann–Whitney; $P = 0.0052$). Pretaporter is not required in dying ECs for ISC proliferation after *P.e.* infection (**J**). The mean mitoses per midgut increased in *P.e.*-infected control midguts ($n = 17$ midguts from one experiment) compared to uninfected control midguts ($n = 17$ midguts from one experiment) (Mann–Whitney; $P = 0.0003$). The mean mitoses per midgut did not significantly decrease in *P.e.*-infected midguts expressing *pretaporter*^{RNAi} in ECs ($n = 17$ midguts from one experiment) compared to *P.e.*-infected control midguts (Mann–Whitney; $P = 0.1619$). Mean (red line) and 95% confidence intervals (blue bars) are shown. Scales bars in (**A**, **B**, **C**, **D**, **F**, **G**) are 20 µm. Significance levels are indicated by $^{*}P \leq 0.05$, $^{**}P \leq 0.01$, $^{***}P \leq 0.001$, $^{****}P \leq 0.0001$, and ns, for not significant ($P > 0.05$). Source data are available online for this figure.

© The Author(s)

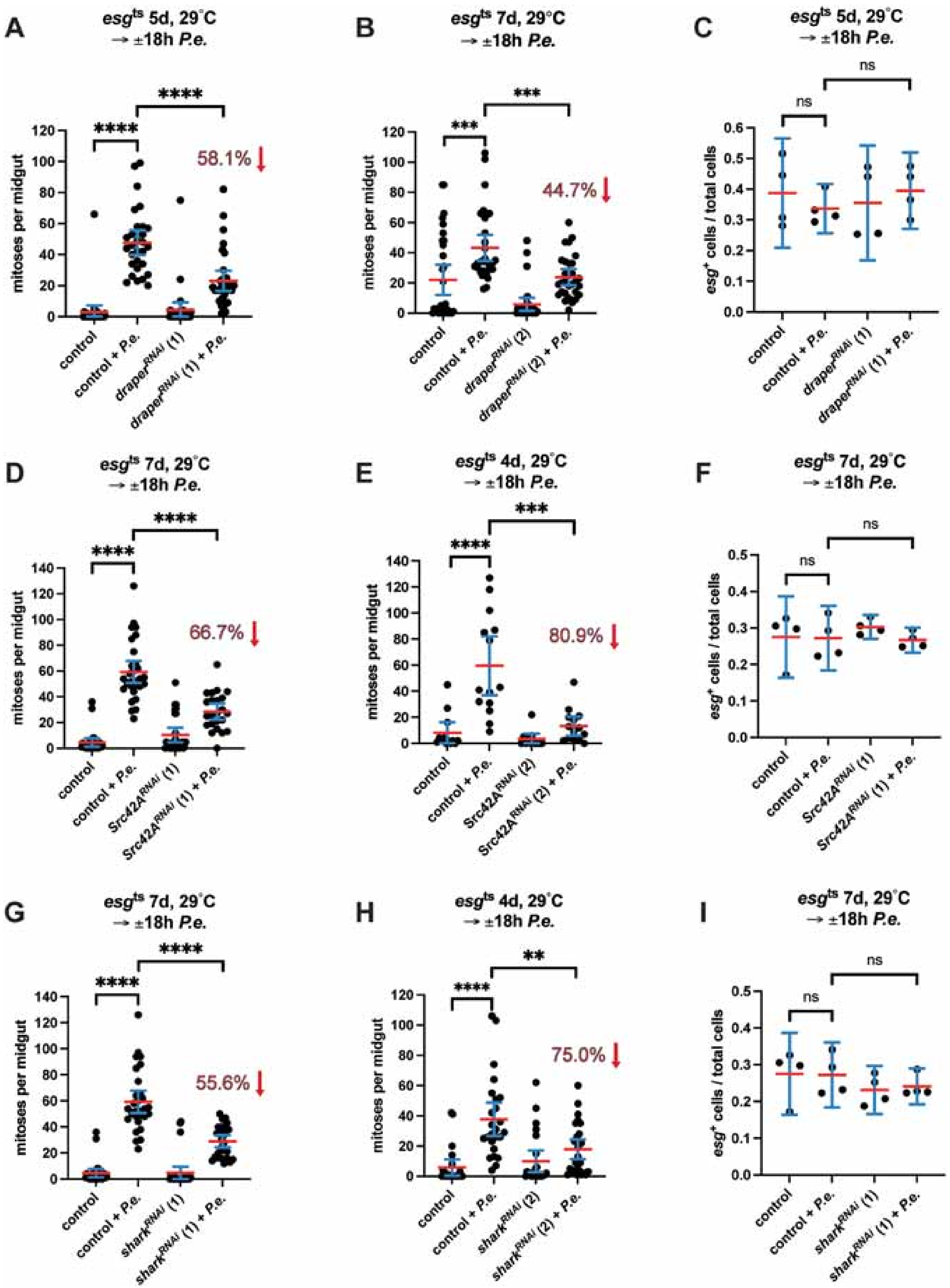
A
esgts 5d, 29°C
→ ±18h P.e.
mitoses per midgut
58.1%
control
control + P.e.
draperRNAi (1)
draperRNAi (1) + P.e.
B
esgts 7d, 29°C
→ ±18h P.e.
mitoses per midgut
44.7%
control
control + P.e.
draperRNAi (2)
draperRNAi (2) + P.e.
C
esgts 5d, 29°C
→ ±18h P.e.
esg+ cells / total cells
ns
control
control + P.e.
draperRNAi (1)
draperRNAi (1) + P.e.
D
esgts 7d, 29°C
→ ±18h P.e.
mitoses per midgut
66.7%
control
control + P.e.
Src42ARNAi (1)
Src42ARNAi (1) + P.e.
E
esgts 4d, 29°C
→ ±18h P.e.
mitoses per midgut
80.9%
control
control + P.e.
Src42ARNAi (2)
Src42ARNAi (2) + P.e.
F
esgts 7d, 29°C
→ ±18h P.e.
esg+ cells / total cells
ns
control
control + P.e.
Src42ARNAi (1)
Src42ARNAi (1) + P.e.
G
esgts 7d, 29°C
→ ±18h P.e.
mitoses per midgut
55.6%
control
control + P.e.
sharkRNAi (1)
sharkRNAi (1) + P.e.
H
esgts 4d, 29°C
→ ±18h P.e.
mitoses per midgut
75.0%
control
control + P.e.
sharkRNAi (2)
sharkRNAi (2) + P.e.
I
esgts 7d, 29°C
→ ±18h P.e.
esg+ cells / total cells
ns
control
control + P.e.
sharkRNAi (1)
sharkRNAi (1) + P.e.

© The Author(s)

Figure EV2. Draper-Src-Shark signalling in progenitors promotes ISC proliferation after midgut damage.

Draper is required in progenitors to promote ISC proliferation after *P.e.* infection (**A**, **B**). The mean mitoses per midgut increased in *P.e.*-infected control midguts ($n = 29$ midguts from two experiments (**A**) and $n = 31$ midguts from two experiments (**B**)) compared to uninfected control midguts ($n = 31$ midguts from two experiments (**A**) and $n = 32$ midguts from two experiments (**B**)) (Mann–Whitney; $P < 0.0001$ (**A**) and $P = 0.0009$ (**B**)). The mean mitoses per midgut decreased in *P.e.*-infected midguts expressing $draper^{RNAi}$ in progenitors ($n = 32$ midguts from two experiments (**A**) and $n = 30$ midguts from two experiments (**B**)) compared to *P.e.*-infected control midguts (Mann–Whitney; $P < 0.0001$ (**A**) and $P = 0.0001$ (**B**)). Depleting *draper* from progenitors does not affect their number after *P.e.*-infection (**C**). There was no significant difference between the mean ratio of esg^+ cells/total cells in *P.e.*-infected control midguts ($n = 4$ midguts from one of two experiments) compared to uninfected control midguts ($n = 4$ midguts from one of two experiments) (unpaired *t* test; $P = 0.4419$). The mean ratio of esg^+ cells/total cells was similar in *P.e.*-infected midguts expressing $draper^{RNAi}$ in progenitors ($n = 4$ midguts from one of two experiments) compared to *P.e.*-infected control midguts (unpaired *t* test; $P = 0.2557$). Src is required in progenitors to promote ISC proliferation after *P.e.* infection (**D**, **E**). The mean mitoses per midgut increased in *P.e.*-infected control midguts ($n = 29$ midguts from two experiments (**D**) and $n = 14$ midguts from one experiment (**E**)) compared to uninfected control midguts ($n = 27$ midguts from two experiments (**D**) and $n = 13$ midguts from one experiment (**E**)) (Mann–Whitney; $P < 0.0001$ (**D**) and $P < 0.0001$ (**E**)). The mean mitoses per midgut decreased in *P.e.*-infected midguts expressing $Src42A^{RNAi}$ in progenitors ($n = 22$ midguts from two experiments (**D**) and $n = 14$ midguts from one experiment (**E**)) compared to *P.e.*-infected control midguts (Mann–Whitney; $P < 0.0001$ (**D**) and $P = 0.0001$ (**E**)). Depleting *Src42A* in progenitors does not affect their number after *P.e.* infection (**F**). There was no significant difference between the mean ratio esg^+ cells/total cells in *P.e.*-infected control midguts ($n = 4$ midguts from one of two experiments) compared to uninfected control midguts ($n = 4$ midguts from one of two experiments) (unpaired *t* test; $P = 0.9484$). The mean ratio esg^+ cells/total cells was similar in *P.e.*-infected midguts expressing $Src42A^{RNAi}$ in progenitors ($n = 4$ midguts from one of two experiments) compared to *P.e.*-infected control midguts (unpaired *t* test; $P = 0.8602$). Shark is required in progenitors to promote ISC proliferation after *P.e.* infection (**G**, **H**). The mean mitoses per midgut increased in *P.e.*-infected control midguts ($n = 29$ midguts from two experiments (**G**) and $n = 25$ midguts from two experiments (**H**)) compared to uninfected control midguts ($n = 27$ midguts from two experiments (**G**) and $n = 23$ midguts from two experiments (**H**)) (Mann–Whitney; $P < 0.0001$ (**G**) and $P < 0.0001$ (**H**)). The mean mitoses per midgut decreased in *P.e.*-infected midguts expressing $shark^{RNAi}$ in progenitors ($n = 27$ midguts from two experiments (**G**) and $n = 27$ midguts from two experiments (**H**)) compared to *P.e.*-infected control midguts (Mann–Whitney; $P < 0.0001$ (**G**) and $P = 0.0013$ (**H**)). Depleting *shark* in progenitors does not affect their number after *P.e.*-infection (**I**). There was no significant difference between the mean ratio of esg^+ cells/total cells in *P.e.*-infected control midguts ($n = 4$ midguts from one of two experiments) compared to uninfected control midguts ($n = 4$ midguts from one of two experiments) (unpaired *t* test; $P = 0.9484$). The mean ratio esg^+ cells/total cells in *P.e.*-infected midguts expressing $shark^{RNAi}$ in progenitors ($n = 4$ midguts from one of two experiments) was similar to the ratio in *P.e.*-infected control midguts (unpaired *t* test; $P = 0.3649$). Mean (red line) and 95% confidence intervals (blue bars) are shown. Significance levels are indicated by $^*P \leq 0.05$, $^{**}P \leq 0.01$, $^{***}P \leq 0.001$, $^{****}P \leq 0.0001$, and ns, for not significant ($P > 0.05$). Source data are available online for this figure.

 © The Author(s)

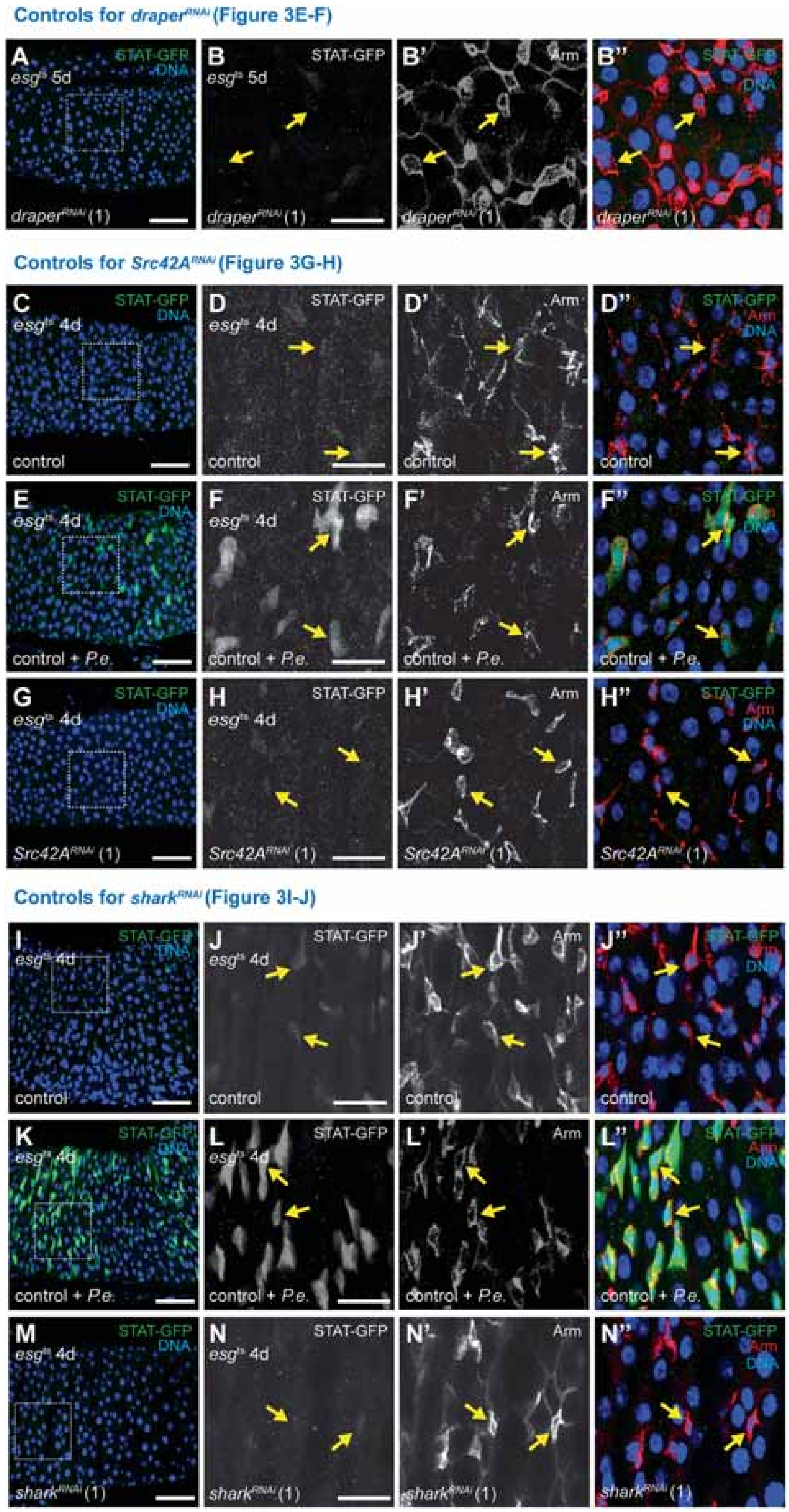
Controls for draper^RNAi (Figure 3E-F)
A STAT-GFP DNA esg^ts 5d draper^RNAi (1)
B STAT-GFP esg^ts 5d draper^RNAi (1)
B' Arm draper^RNAi (1)
B'' STAT-GFP DNA draper^RNAi (1)
Controls for Src42A^RNAi (Figure 3G-H)
C STAT-GFP DNA esg^ts 4d control
D STAT-GFP esg^ts 4d control
D' Arm control
D'' STAT-GFP Arm DNA control
E STAT-GFP DNA esg^ts 4d control + P.e.
F STAT-GFP esg^ts 4d control + P.e.
F' Arm control + P.e.
F'' STAT-GFP Arm DNA control + P.e.
G STAT-GFP DNA esg^ts 4d Src42A^RNAi (1)
H STAT-GFP esg^ts 4d Src42A^RNAi (1)
H' Arm Src42A^RNAi (1)
H'' STAT-GFP Arm DNA Src42A^RNAi (1)
Controls for shark^RNAi (Figure 3I-J)
I STAT-GFP DNA esg^ts 4d control
J STAT-GFP esg^ts 4d control
J' Arm control
J'' STAT-GFP Arm DNA control
K STAT-GFP DNA esg^ts 4d control + P.e.
L STAT-GFP esg^ts 4d control + P.e.
L' Arm control + P.e.
L'' STAT-GFP Arm DNA control + P.e.
M STAT-GFP DNA esg^ts 4d shark^RNAi (1)
N STAT-GFP esg^ts 4d shark^RNAi (1)
N' Arm shark^RNAi (1)
N'' STAT-GFP Arm DNA shark^RNAi (1)

Figure EV3. Draper-Src-Shark signalling promotes STAT activity in progenitors after midgut damage; inhibiting Draper-Src-Shark signalling does not affect basal STAT activity in progenitors.

Draper does not affect basal STAT activity in progenitors (**A**, **B**). STAT activity (STAT-GFP (*10X STAT-dGFP*); green) in progenitors (Arm^{high}, red; yellow arrows) expressing *draper^{RNAi}* in uninfected midguts (**A–B″**) was similar to control uninfected midguts. STAT is activated in progenitors after *P.e.* infection (**C–F**, **I–L**). STAT activity increased in progenitors of *P.e.*-infected control midguts (**E–F″**, **K–L″**) compared to uninfected control midguts (**C–D″**, **I–J″**). Depleting Src and Shark in progenitors does not affect their basal STAT activity (**G–H**, **M–N**). STAT activity in progenitors expressing *Src42A^{RNAi}* in uninfected midguts (**G–H″**) was similar to that in uninfected control midguts (**C–D″**). STAT activity in progenitors expressing *shark^{RNAi}* in uninfected midguts (**M–N″**) was similar to that in uninfected control midguts (**I–J″**). Scale bars in (**A**, **C**, **E**, **G**, **I**, **K**, **M**) are 50 µm and in (**B**, **D**, **F**, **H**, **J**, **L**, **N**) are 20 µm. Significance levels are indicated by $*P \le 0.05$, $**P \le 0.01$, $***P \le 0.001$, $****P \le 0.0001$, and ns, for not significant ($P > 0.05$). Source data are available online for this figure.

 © The Author(s)

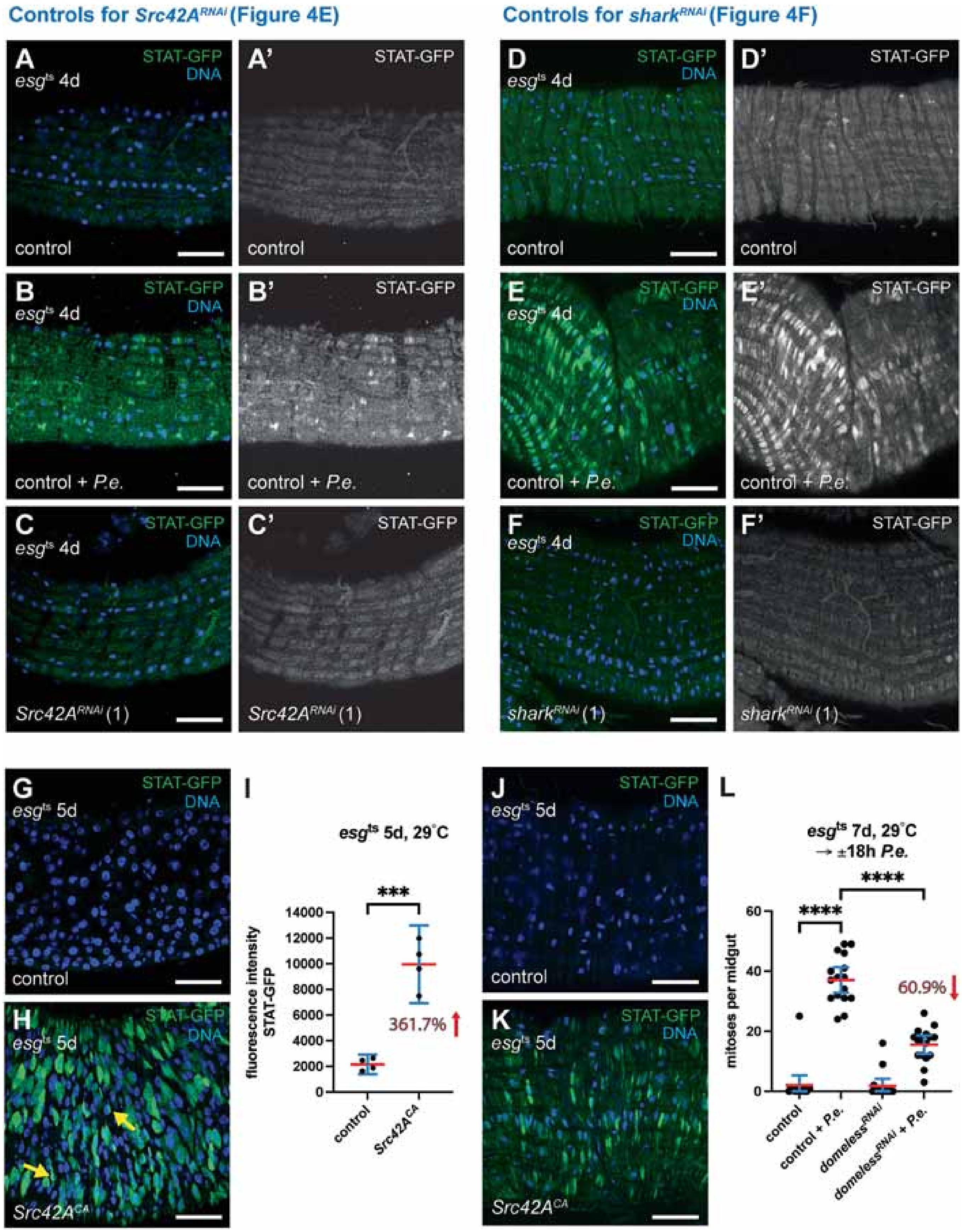
Controls for Src42ARNAi (Figure 4E)
Controls for sharkRNAi (Figure 4F)
A
esgts 4d
STAT-GFP
DNA
control
A'
STAT-GFP
control
B
esgts 4d
STAT-GFP
DNA
control + P.e.
B'
STAT-GFP
control + P.e.
C
esgts 4d
STAT-GFP
DNA
Src42ARNAi (1)
C'
STAT-GFP
Src42ARNAi (1)
D
esgts 4d
STAT-GFP
DNA
control
D'
STAT-GFP
control
E
esgts 4d
STAT-GFP
DNA
control + P.e.
E'
STAT-GFP
control + P.e.
F
esgts 4d
STAT-GFP
DNA
sharkRNAi (1)
F'
STAT-GFP
sharkRNAi (1)
G
esgts 5d
STAT-GFP
DNA
control
H
esgts 5d
STAT-GFP
DNA
Src42ACA
I
esgts 5d, 29°C
***
fluorescence intensity STAT-GFP
14000
12000
10000
8000
6000
4000
2000
0
361.7%
control
Src42ACA
J
esgts 5d
STAT-GFP
DNA
control
K
esgts 5d
STAT-GFP
DNA
Src42ACA
L
esgts 7d, 29°C
→ ±18h P.e.
****
****
mitoses per midgut
60
40
20
0
60.9%
control
control + P.e.
domelessRNAi
domelessRNAi + P.e.

◀ **Figure EV4. Draper-Src-Shark signalling is sufficient to increase STAT activity in progenitors and in the visceral muscle; inhibiting Src-Shark signalling in midgut progenitors does not affect basal STAT activity in the visceral muscle.**

STAT is activated in the visceral muscle (VM) after *P.e.* infection (**A**–**B**, **D**–**E**). STAT activity (STAT-GFP (*10X STAT-dGFP*); green) increased in the VM of *P.e.*-infected control midguts (**B**–**B′**, **E**–**E′**) compared to uninfected control midguts (**A**–**A′**, **D**–**D′**). Depleting *Src42A* and *shark* does not affect basal STAT activity in the VM (**C**, **F**). STAT activity in the VM of uninfected midguts expressing $Src42A^{RNAi}$ or $shark^{RNAi}$ in progenitors (**C**–**C′**, **F**–**F′**) was similar to the STAT activity in the VM of uninfected control midguts (**A**–**A′**, **D**–**D′**). Constitutively active Src42A signalling in progenitors induces STAT in both the progenitors and in the VM (**G**–**K**). STAT activation increased in progenitors (yellow arrows) expressing $Src42A^{CA}$ (**H**) compared to control midguts (**G**). Mean STAT levels increased in progenitors expressing $Src42A^{CA}$ ($n = 4$ midguts from one experiment) compared to those in control midguts ($n = 4$ midguts from one experiment) (unpaired *t* tests; $P = 0.0002$) (**I**). STAT activation increased in the VM of midguts expressing $Src42A^{CA}$ in progenitors (**K**) compared to control midguts (**J**). Domeless is required in progenitors to promote ISC proliferation after midgut damage (**L**). The mean mitoses per midgut increased in *P.e.*-infected control midguts ($n = 16$ midguts from one experiment) compared to uninfected control midguts ($n = 16$ midguts from one experiment) (Mann–Whitney; $P < 0.0001$). The mean mitoses per midgut decreased in *P.e.*-infected midguts expressing $dome^{RNAi}$ in progenitors ($n = 16$ midguts from one experiment) compared to *P.e.*-infected control midguts (Mann–Whitney; $P < 0.0001$). Mean (red line) and 95% confidence intervals (blue bars) are shown. Scale bars in (**A**, **B**, **C**, **D**, **E**, **F**, **G**, **H**, **J**, **K**) are 50 µm. Significance levels are indicated by *$P \le 0.05$, **$P \le 0.01$, ***$P \le 0.001$, ****$P \le 0.0001$, and ns, for not significant ($P > 0.05$). Source data are available online for this figure.

 © The Author(s)

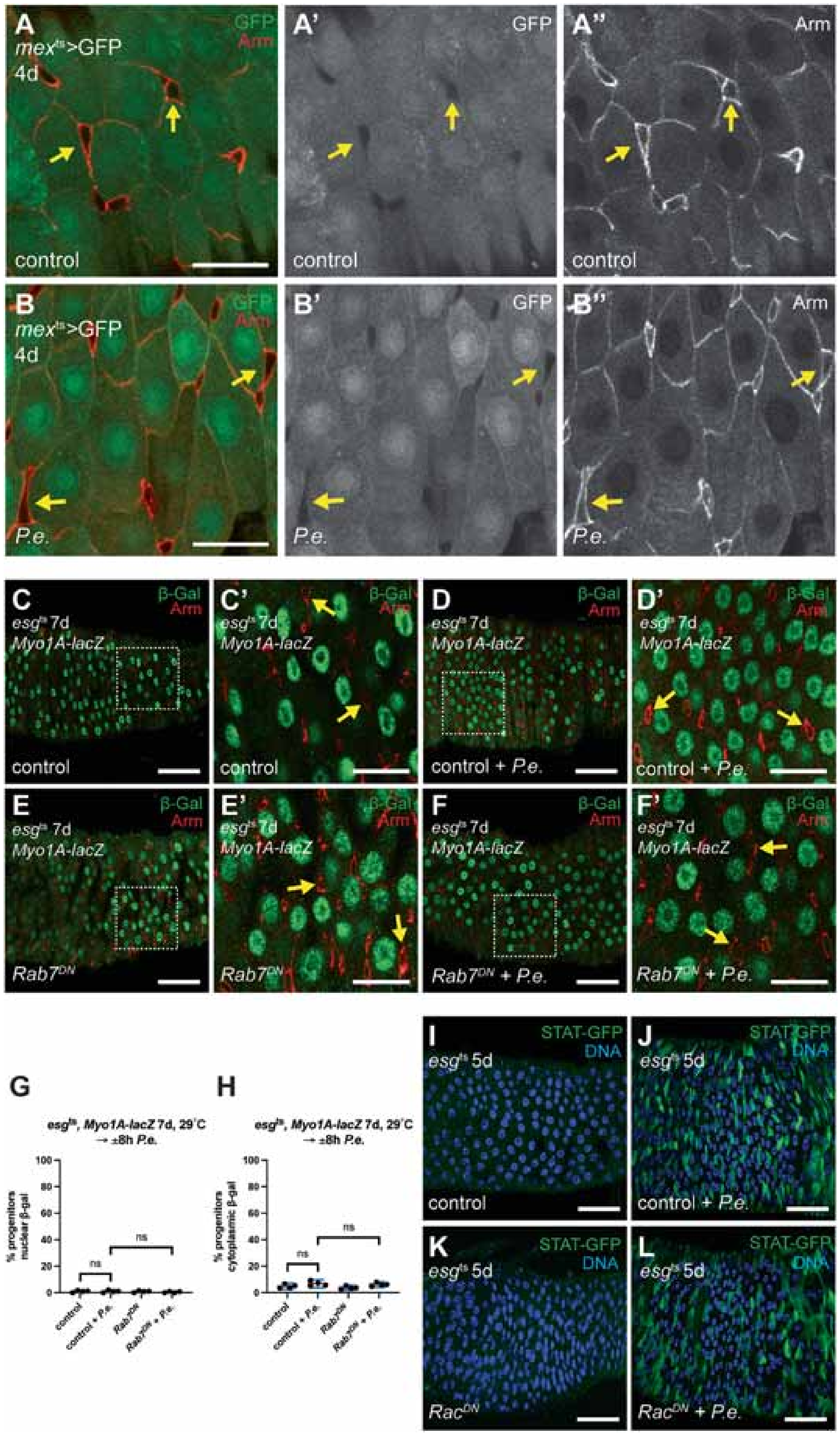
A
mex^ts>GFP
4d
GFP
Arm
control
A'
GFP
control
A''
Arm
control
B
mex^ts>GFP
4d
GFP
Arm
P.e.
B'
GFP
P.e.
B''
Arm
P.e.
C
esg^ts 7d
Myo1A-lacZ
β-Gal
Arm
control
C'
control
D
control + P.e.
D'
control + P.e.
E
Rab7^DN
E'
Rab7^DN
F
Rab7^DN + P.e.
F'
Rab7^DN + P.e.
G
esg^ts, Myo1A-lacZ 7d, 29°C
→ ±8h P.e.
% progenitors nuclear β-gal
ns
control
control + P.e.
Rab7^DN
Rab7^DN + P.e.
H
esg^ts, Myo1A-lacZ 7d, 29°C
→ ±8h P.e.
% progenitors cytoplasmic β-gal
ns
I
esg^ts 5d
STAT-GFP
DNA
control
J
control + P.e.
K
Rac^DN
L
Rac^DN + P.e.

Figure EV5. Midgut progenitors do not clear dying ECs; Rac activity does not promote STAT activity in progenitors after midgut damage.

Dying enterocytes (ECs) are not cleared by progenitors after *P.e.* infection (**A**, **B**). GFP (green) expressed in ECs was not detected within Arm^{high+} (red, yellow arrows) cells in uninfected and *P.e.*-infected midguts (**A–B″**). EC corpses are not detected after blocking lysosomal degradation in progenitors after midgut damage (**C–H**). β-gal-positive (green) EC corpses were detected neither within progenitors (Arm^{high} (red); yellow arrows) of control uninfected and *P.e.*-infected midguts (**C–D′**) nor within *Rab7^{DN}*-expressing progenitors of uninfected and infected midguts (**E–F′**). β-gal was detected within the nuclei of progenitors at the same frequency between uninfected ($n = 4$ midguts from one experiment) and infected control midguts ($n = 4$ midguts from one experiment) (Mann–Whitney; $P > 0.9999$) and between infected midguts with progenitors expressing *Rab7^{DN}* ($n = 4$ midguts from one experiment) and control infected midguts (Mann–Whitney; $P = 0.3463$) (**G**). β-gal was detected within the cytoplasm of progenitors at the same frequency between infected ($n = 4$ midguts from one experiment) and uninfected control midguts ($n = 4$ midguts from one experiment) (Mann–Whitney; $P = 0.1522$) and between infected midguts with progenitors expressing *Rab7^{DN}* ($n = 4$ midguts from one experiment) and control infected midguts (Mann–Whitney; $P = 0.5942$) (**H**). Rac signalling does not promote STAT activity in progenitors after *P.e.* infection (**I–L**). STAT activity (STAT-GFP (*10X STAT-dGFP*); green) increased in progenitors of *P.e.*-infected control midguts (**J**) compared to uninfected control midguts (**I**). STAT activity was similar between progenitors expressing *Rac^{DN}* in *P.e.*-infected midguts (**L**) and those in *P.e.*-infected control midguts (**J**). Mean (red line) and 95% confidence intervals (blue bars) are shown. Scale bars in (**A**, **B**, **C′**, **D′**, **E′**, **F′**) are 20 μm and in (**C, D, E, F**, **I–L**) are 50 μm. Single-slice images are presented in (**A–F′**). Significance levels are indicated by *$P \leq 0.05$, **$P \leq 0.01$, ***$P \leq 0.001$, ****$P \leq 0.0001$, and ns, for not significant ($P > 0.05$). Source data are available online for this figure.

 © The Author(s)
