# Supplementary material for: Damage recognition by intestinal stem cells via Draper promotes adult Drosophila midgut regeneration
Source: EMBO J. 2026 Aug 5;45(18):6450–79. doi: 10.1038/s44318-026-00890-1 (PMC13578246; doi:10.1038/s44318-026-00890-1)
Supplement: Supplementary file 2 [file 44318_2026_890_MOESM1_ESM.pdf]

Appendix for "Damage recognition by intestinal stem cells via Draper promotes adult *Drosophila* midgut regeneration"

Table of Contents

Appendix Figure S1. Draper-Src-Shark signalling promotes STAT activity in progenitors after midgut damage. Pages 2-3.

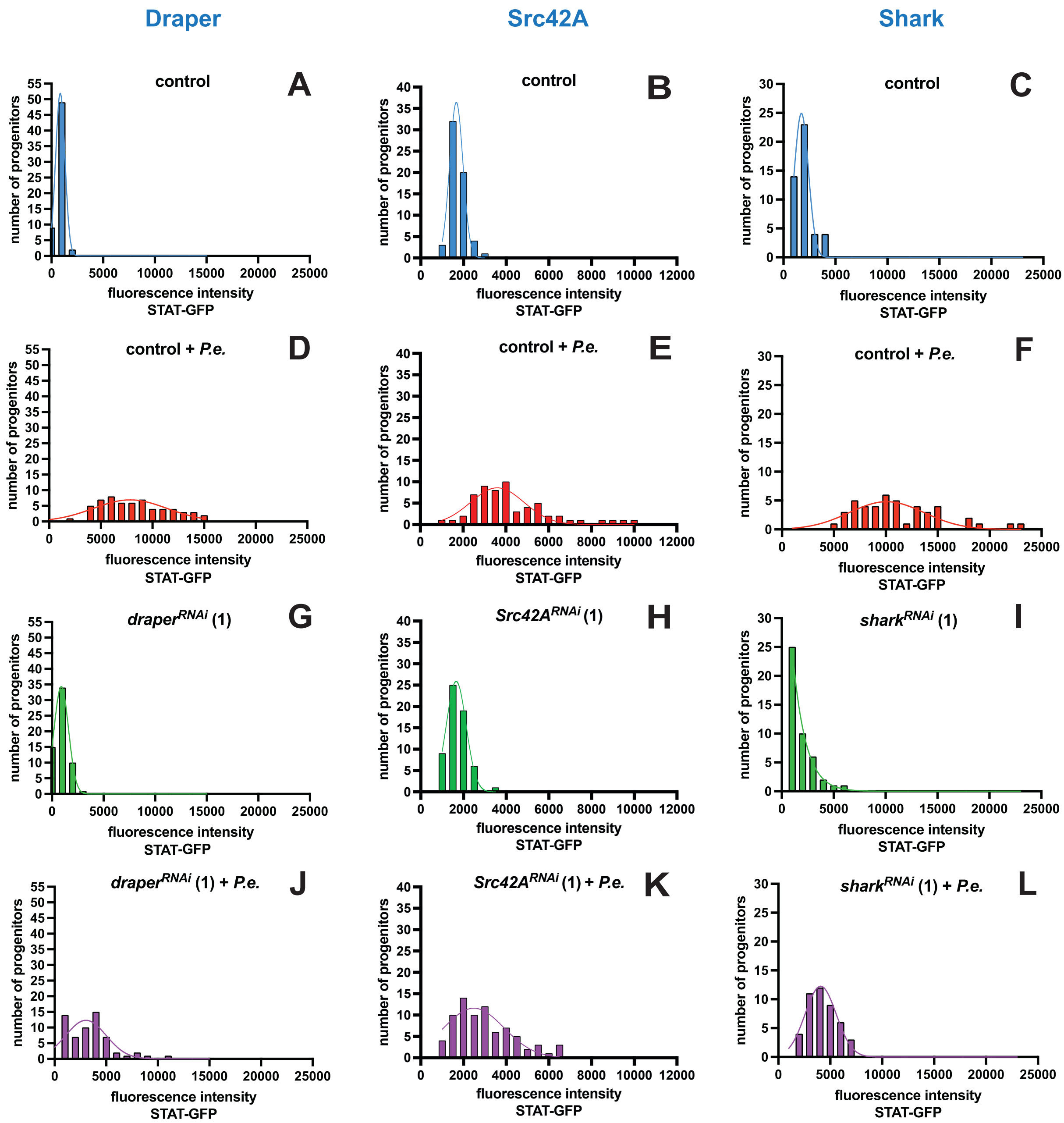

Appendix Figure S1. Draper-Src-Shark signalling promotes STAT activity in progenitors after midgut damage. STAT activity increases in progenitors after *P.e.* infection **(A-F)**. STAT activity (10X STAT-GFP fluorescence intensity) increased in progenitors of *P.e.*-infected control midguts (n=60 cells from four midguts from one of two experiments (**D**), n=60 cells from four midguts from one of two experiments (**E**) and n=45 cells from three midguts from one of three experiments) (**F**) compared to progenitors in uninfected control midguts (n=60 cells from four midguts from one of two experiments (**A**), n=60 cells from four midguts from one of two experiments (**B**) and n=45 cells from three midguts from one of three experiments (**C**)). Draper promotes STAT activity in progenitors after *P.e.* infection **(A, D, G, J)**. STAT activity decreased in progenitors expressing *draper*^{RNAi} in *P.e.*-infected midguts (n=60 cells from four midguts from one of two experiments) (**J**) compared to infected control midguts (**D**). Basal STAT activity in progenitors expressing *draper*^{RNAi} in uninfected midguts (n=60 cells from four

midguts from one of two experiments) (**G**) was similar to progenitors in uninfected control midguts (**A**). Src42A promotes STAT activity in progenitors after *P.e.* infection **(B, E, H, K)**. Stat activity decreased in progenitors expressing *Src42A*^{RNAi} in *P.e.*-infected midguts (n=60 cells from four midguts from one of two experiments) (**K**) compared to infected control midguts (**E**). Basal STAT activity in progenitors expressing *Src42A*^{RNAi} in uninfected midguts (n=60 cells from four midguts from one of two experiments) (**H**) was similar to progenitors in uninfected control midguts (**B**). Shark promotes STAT activity in progenitors after *P.e.* infection **(C, F, I, L)**. Stat activity decreased in progenitors expressing *shark*^{RNAi} in *P.e.*-infected midguts (n=45 cells from three midguts from one of three experiments) (**L**) compared to infected control midguts (**F**). Basal STAT activity in progenitors expressing *shark*^{RNAi} in uninfected midguts (n=45 cells from three midguts from one of three experiments) (**I**) was similar to progenitors in uninfected control midguts (**C**).
