# Supplementary material for: Damage recognition by intestinal stem cells via Draper promotes adult Drosophila midgut regeneration
Source: EMBO J. 2026 Aug 5;45(18):6450–79. doi: 10.1038/s44318-026-00890-1 (PMC13578246; doi:10.1038/s44318-026-00890-1)
Supplement: Supplementary file 3 [file 44318_2026_890_MOESM2_ESM.pdf]

THE EMBO JOURNAL

Damage recognition by intestinal stem cells via Draper promotes adult Drosophila midgut regeneration

Martina Legido, Marie Srotyr, Rebecca Wafer, and Parthive H. Patel

Corresponding authors: Parthive H. Patel (p.patel@bristol.ac.uk)

Review Timeline:

Transferred from Review Commons:	25th Jun 25
Editorial Decision:	9th Jul 25
Appeal:	16th Jul 25
Editorial Decision:	31st Jul 25
Revision Received:	22nd Mar 26
Editorial Decision:	26th Jun 26
Editor's Correspondence:	29th Jun 26
Revision Received:	8th Jul 26
Accepted:	22nd Jul 26

Editor: Daniel Klimmeck

Transaction Report:

This manuscript was transferred to The EMBO Journal following peer review at Review Commons.

(Note: With the exception of the correction of typographical or spelling errors that could be a source of ambiguity, letters and reports are not edited. Depending on transfer agreements, referee reports obtained elsewhere may or may not be included in this compilation. Referee reports are anonymous unless the Referee chooses to sign their reports.)

Review #1

1. Evidence, reproducibility and clarity:

Evidence, reproducibility and clarity (Required)

The manuscript by Legido et al. reports the discovery of a previously unappreciated damage-sensing mechanism in Drosophila intestinal stem cells (ISCs). The authors identify a Draper-Src42A-Shark signaling axis that enables ISCs to recognize damage-induced apoptosis in neighboring epithelial cells and promotes regeneration by activating STAT signaling. The work demonstrates that this pathway functions cell-autonomously within ISCs to promote proliferation and also non-cell-autonomously to activate STAT in the visceral muscle (VM), thereby influencing the regenerative niche. The results presented here are intriguing; however there is considerable room for improvement in the presentation of the experimental system, the results, and the interpretations of the data. Additional controls are required.

Major issues are listed below.

1. The manuscript is poorly organized. The text jumps from Figure 1 to Figure 3 before discussing Figure 2, making the paper difficult to read.

- A schematic diagram of the tissue and cell types should be presented as Figure 1A to orient readers who might not work on this particular tissue.
Please show or schematize and provide references for the Gal4 expression patterns.
2. The effect of reducing Drpr, Src, or Shark on the STAT transcriptional reporter damage response seems to be about two-fold. Is that sufficient to explain the effects on ISC proliferation? Do STAT heterozygotes have an effect? Does hyperactivation of STAT have an effect?
3. The authors assume that Draper's function is recognizing PS on apoptotic cells and activating Src as it does in other contexts, but they should test this by addressing the following questions.

a. Does the p35 expression in EC also block pSrc in the progenitors? This would help connect apoptosis to Drpr-Src-Shark signaling.

b. How does Drpr RNAi affect pSrc in progenitors?

c. Is PS recognition required or the domain that recognizes it on Drpr required? Is the ITAM domain of Drpr required?
4. The claim that Draper serves a function other than stimulating engulfment requires stronger experimental support. Currently this interpretation rests solely on a negative result (absence of beta-gal-expressing nuclei within ISCs). It is possible that the apoptotic cells are extruded and are therefore in another focal plane. They may be engulfed and degraded rapidly and therefore not accumulating. The progenitor cells could be nibbling but not engulfing entire cells, so the authors should test that by labeling membranes of the target cells. The authors should demonstrate that they actually can detect engulfment by including a positive control. Live imaging also would be helpful to see where the apoptotic cells are going if they are not engulfed.

5. No data are provided to support the claim that Draper-Src-Shark-STAT signaling operates independently of the cytokines (Upd1-3) and their receptor Domeless. It would be relatively straightforward to look at Upd expression or test the effects of expressing dominant-negative Domeless in specific cell subsets (ISCs and VMs) using available Gal4 drivers.
6. No data are provided to support the claim that phosphatidylserine on the membrane of apoptotic enterocytes activates Draper/Src/Shark signaling.
7. No mechanism is tested or proposed for how Src activates STAT.
8. The non-autonomous effect is surprising but unexplored. A potential mechanism that would explain both how Draper-Src-Shark activates STAT in the ISC and in the muscle would be if Upds are secreted, which is a well-established effect of injury.

Minor issues

1. ISC is not defined when first mentioned
2. Figure 1D-G, show representative images along with quantifications.
Why are the scales different on the different STAT graphs? makes it hard to compare between the figures.
Why are axes different numbers in 1F ?
3. Please move Supplemental figures 1C, 2C 3C into the main figures

2. Significance:

Significance (Required)

This work in principle provides a conceptual advance in stem cell biology and regeneration by proposing a new function for the molecular machinery best known for innate immune responses: specifically that the Draper - Src - Shark pathway that is best characterized in phagocytosis plays a role in stimulating STAT upon tissue damage. This work will be of interest to basic scientists studying stem cell responses to tissue injury. It has relevance for tissue repair, regeneration, and aging. The limitations of the study are described above and include the confusing presentation as well as missing controls and insufficient data to support some of the conclusions.

3. How much time do you estimate the authors will need to complete the suggested revisions:

Estimated time to Complete Revisions (Required)

(Decision Recommendation)

More than 6 months

4. *Review Commons* values the work of reviewers and encourages them to get credit for their work. Select 'Yes'

below to register your reviewing activity at [Web of Science Reviewer Recognition Service]() (formerly Publons); note that the content of your review will not be visible on Web of Science.

Web of Science Reviewer Recognition

Yes

Review #2

1. Evidence, reproducibility and clarity:

Evidence, reproducibility and clarity (Required)

Damage recognition by intestinal stem cells via Draper-Src-Shark-STAT signalling promotes adult Drosophila midgut regeneration.

In this manuscript, Legido et al present evidence that the Src42A kinase in Drosophila is essential for epithelial regeneration in damaged fly midguts. The work is performed to a very high standard; images are excellent throughout; statistics are appropriate, and sample sizes are high; the authors clarify size effects throughout their work; all data are easily followed and appropriately interpreted.

As a result, I am highly enthusiastic about the value and quality of this study and commend the authors for their work. Most of my concerns are relatively minor and are detailed below.

Major issues

1. Src as a bacteria sensor. I struggle with the rationale behind Figure 1E. The authors claim these data exclude possible roles for Src as a molecule that "detects pathogenic bacteria". I may be incorrect, but it appears the experiment is intended to test if Src is a classical pattern recognition receptor (PRR) that senses microbial patterns. It's unclear what would lead to this hypothesis, as Src does not have the molecular structure or prior literature we would expect to see in PRRs. Nonetheless, I do not agree that the results exclude roles for Src in sensing bacterial patterns (regardless of how unlikely the possibility is). The experiment in Figure 1E was performed with conventional flies with a conventional microbiome, so we cannot formally exclude the possibility that Src "senses" dysbiotic commensal bacterial patterns in an SDS-treated gut. To exclude this possibility, it would be important to repeat Figure 1E in axenic flies. For me, the data in Figure E are valuable, but primarily because they demonstrate that Src is also capable of initiating repair in response to chemical stress, suggesting that the phenotype is not exclusive to a unique feature of Pe infection. If the authors wish to exclude roles for Src as a

PRR, they should be encouraged to stress axenic guts. As it is highly unlikely that Src is a PRR, I feel the authors would be best advised to consider reorganizing the text to clarify that their work extends prior observations by showing that Src also promotes renewal in response to a chemical stress.
2. Possible involvement of the UPD/Domeless pathway. At times, it appears that the authors argue their data exclude roles for UPD signaling in STAT activation by Src (e.g. penultimate sentence of Introduction, relevant text in Results section, paragraph of Discussion). The authors are correct that prior studies argue against Domeless involvement, but none of the experiments in this manuscript test that hypothesis further. As a result, I encourage the authors to be more precise in their discussion of Upd signaling.

Minor Issues

1. Regenerative Niche: I struggle with the concept of a regenerative niche in the fly gut. As the authors point out essentially all intestinal cells (enteroblasts, enterocytes, enteroendocrine, muscle, trachea) influence stem cell behaviour in the fly gut. As a result, it's difficult to agree that there is a subset of specialist "niche" cells that specifically regulate stem cell proliferation in the fly midgut. I find the term "microenvironment" (used in the Introduction) more appropriate for flies.
2. This sentence from the Introduction requires modification: "Mechanistically, Draper-Src-Shark signalling stimulates ISC proliferation by increasing STAT activity in ISCs, which has previously been thought to be exclusively activated by cytokine (Upd1-3)/JAK signalling." As the authors point out at an earlier stage, prior work had already established that Upd1-3 are not needed for Src-dependent STAT activation: "Domeless and its ligands, Upd1-3, are not required for Src42A to stimulate ISC proliferation through STAT activation".
3. Methods: More detail is needed for the infection protocol. The authors should detail their handling of Pe (growth medium; all steps taken to centrifuge, resuspend, etc. Pe prior to infection; definition of 20X Pe ideally as CFU/ml, or OD). Otherwise, it will be very difficult for others to follow up on this study. Also, please clarify if females used in these experiments were mated or virgin.
4. Quantifications: When introducing data in Figure 3, please consider including one or two sentences explaining what we are looking at in panels L-O. For many within the field, it is likely intuitive, but I wonder if those less familiar with Drosophila will follow along.
5. Organization of data. For now, the arrangement of the figures is slightly confusing, and seldom aligns with the text. I find this diminishes the quality of the manuscript. I recommend the authors consider rearranging all figures to directly match the text. For example, STAT:GFP images and quantifications are discussed in the section "Draper-Src-Shark signalling promotes ISC proliferation through STAT activation". It would make most sense for these data to all exist in a single matching figure. These issues apply throughout (Figure 3 and 4 are discussed prior to Figure 2, text for Figure 5 follows text for figure 6, etc.). This does not require any additional experiments but would make the text much more accessible to readers.
6. Figure 7: I appreciate the inclusion of a speculative model in Figure 7. However, I feel the accompanying text is more appropriate for the discussion than the result, and there are cases where the wording could be moderate (e.g. "This allows cortically-localised activated Src to phosphorylate the ITAMs on Draper" is not supported by data demonstrating phosphorylation of ITAMs on Draper by Src).

7. Returning to the theme of Src as a potential PRR, the following sentence from the discussion confuses me: "This suggests that Src42A in ISCs senses tissue damage rather than pathogenic bacteria themselves, possibly through the recognition of pathogen-associated molecular patterns (PAMPs)." Here, the authors appear to suggest that Src detects PAMPs. Is that correct? The hypothesis is not supported by the accompanying data. Presumably they are arguing that Src does not detect PAMP. If so, the text could be clarified. Additionally, I would encourage the authors to consider microbe-associated molecular pattern (MAMP) over PAMP.

2. Significance:

Significance (Required)

The data are consistent with earlier reports by Cordero et al (EMBO 2014), and Kohlmaier et al. (Oncogene 2015). However, this study goes further by presenting two important discoveries. First the authors present convincing genetic evidence that Src-dependent regeneration involves participation of Draper and Shark. Secondly, the authors provide data that indicate Draper initiates Src signaling in a phagocytosis-independent manner. As a result, the work constitutes an important extension of previous work and adds to our understanding of epithelial regeneration in fly guts. The exact mechanism by which dying enterocytes activate Draper in ISCs requires clarification, and possible involvement of visceral muscle hinted at in figure six also requires clarification. However, the current study will doubtlessly be of interest to researchers using flies to characterize epithelial cell dynamics during periods of stress and should be of appeal to a larger community interested in epithelial regeneration.

3. How much time do you estimate the authors will need to complete the suggested revisions:

Estimated time to Complete Revisions (Required)

(Decision Recommendation)

Between 1 and 3 months

4. *Review Commons* values the work of reviewers and encourages them to get credit for their work. Select 'Yes' below to register your reviewing activity at Web of Science Reviewer Recognition Service (formerly Publons); note that the content of your review will not be visible on Web of Science.

Web of Science Reviewer Recognition

Yes

Review #3

1. Evidence, reproducibility and clarity:

Evidence, reproducibility and clarity (Required)

In this manuscript, Legido et al. investigate the role of Draper-Src-Shark-STAT signaling in regulating intestinal stem cell (ISC) proliferation upon bacterial infection using the Drosophila intestine model. They demonstrate that Draper-Src-Shark signaling acts in ISCs to recognize midgut damage and is required for ISC proliferation following bacterial infection. In addition, they show that Draper-Src-Shark signaling regulates STAT activation both in progenitor cells and the visceral muscle.

Major concerns:

1. While the authors use Mex-Gal4 to drive gene expression in ECs and examine pSrc, it would be better to co-express UAS-GFP to clearly mark ECs.
2. It is difficult to see the upregulation of pSrc in ECs (Supplementary Fig. 1D-G'). To confirm the specificity of the pSrc antibody, the authors should knock down Src42 using esg-Gal4 during P.e. infection and examine the results.
3. Although the authors show that Shark is required for Src42A-mediated ISC proliferation, they should also test whether Src42A is required for Draper-induced ISC proliferation to complete the signaling pathway analysis.
4. The authors conclude that "these data indicate that Draper-Src-Shark signaling promotes ISC proliferation through STAT activation." However, the current data only show that STAT activation upon P.e. infection depends on Draper-Src-Shark signaling. To strengthen this conclusion, the authors should activate Draper-Src-Shark signaling and determine whether STAT knockdown can rescue the ISC proliferation phenotype.
5. The authors state that "p35 expression in ECs partially blocked ISC proliferation by 40.1% in P.e.-infected midguts compared to controls (Fig. 5A), suggesting that some infected ECs undergo apoptosis." However, they should directly examine apoptosis markers in response to P.e. infection to support this claim.
6. To further test the relationship between apoptosis and Draper-Src-Shark signaling, the authors should induce apoptosis in ECs while blocking Draper-Src-Shark signaling and assess whether ISC proliferation is rescued.
7. The authors demonstrate that Draper-Src-Shark signaling in progenitor cells regulates STAT activity non-cell autonomously in the visceral muscle. To strengthen this conclusion, they should activate Draper-Src-Shark signaling specifically in progenitor cells and examine STAT-GFP levels in muscle cells.

Minor concerns:

- The authors claim that "Draper-Src-Shark signaling acts in ISCs to recognize midgut damage and is required for full STAT activation in ISCs to promote their proliferation." However, Draper-Src-Shark signaling does not seem to completely block STAT activation in response to P.e. infection. The authors should review the manuscript carefully and revise claims that are not fully supported by the experimental data.

2. Significance:

Significance (Required)

Although the role of Src42A in ISC proliferation upon bacterial infection has been previously reported, the mechanism elucidated in this study provides new insights into intestinal stem cell regulation and could contribute meaningfully to the stem cell field.

3. How much time do you estimate the authors will need to complete the suggested revisions:

Estimated time to Complete Revisions (Required)

(Decision Recommendation)

Between 3 and 6 months

4. *Review Commons* values the work of reviewers and encourages them to get credit for their work. Select 'Yes' below to register your reviewing activity at Web of Science Reviewer Recognition Service (formerly Publons); note that the content of your review will not be visible on Web of Science.

Web of Science Reviewer Recognition

Yes

Revision Plan

Manuscript number: RC-2025-02969
Corresponding author(s): Parthive H. Patel

[The "revision plan" should delineate the revisions that authors intend to carry out in response to the points raised by the referees. It also provides the authors with the opportunity to explain their view of the paper and of the referee reports.

The document is important for the editors of affiliate journals when they make a first decision on the transferred manuscript. It will also be useful to readers of the reprint and help them to obtain a balanced view of the paper.

If you wish to submit a full revision, please use our "Full Revision" template. ***It is important to use the appropriate template to clearly inform the editors of your intentions.****]*

1. General Statements [optional]

This section is optional. Insert here any general statements you wish to make about the goal of the study or about the reviews.

We thank the reviewers for their careful assessment of our work, which will help us further improve our manuscript. Below we have outlined our plans for our revision.

Our work shows that Draper-Src-Shark signalling acts in ISCs to recognise midgut damage and is required for Stat activation in ISCs to promote their proliferation. To our knowledge, this is the first description of a signalling pathway (from receptor to transcriptional activity) describing how ISCs can recognise midgut damage and translate this to their proliferation. This damage recognition pathway works in parallel with other niche signals such as cytokines (Upd1-3) to promote ISC proliferation after midgut damage. Currently, our data indicate that Draper signalling in ISCs does not promote engulfment of dying midgut epithelial cells but is required for ISC proliferation in the presence of dying enterocytes. Moreover, we show that Draper-Src-Shark signalling in progenitors regulates Stat activity non-cell autonomously in the visceral muscle- a component of the regenerative niche.

This importance of Draper-Src-Shark signalling in ISCs for midgut regeneration would still stand even if ISCs were found to engulf apoptotic cells or if ISCs detected damage rather than apoptosis. Further, our data currently suggests that Draper-Src-Shark signalling promotes Stat activation and ISC proliferation independently of cytokines. However, if Draper-Src-Shark signaling is found to promote cytokine production in progenitors, this would be the first mechanism described to induce cytokine production in progenitors after midgut damage.

2. Description of the planned revisions

Insert here a point-by-point reply that explains what revisions, additional experimentations and analyses are planned to address the points raised by the referees.

Reviewer 1

Reviewer #1 (Evidence, reproducibility and clarity (Required)):

The manuscript by Legido et al. reports the discovery of a previously unappreciated damage-sensing mechanism in Drosophila intestinal stem cells (ISCs). The authors identify a Draper-Src42A-Shark signaling axis that enables ISCs to recognize damage-induced apoptosis in neighboring epithelial cells and promotes regeneration by activating STAT signaling. The work demonstrates that this pathway functions cell-autonomously within ISCs to promote proliferation and also non-cell-autonomously to activate STAT in the visceral muscle (VM), thereby influencing the regenerative niche. The results presented here are intriguing; however there is considerable room for improvement in the presentation of the experimental system, the results, and the interpretations of the data. Additional controls are required.

We are delighted that the reviewer found our findings intriguing and thank them for their suggestions to improve the manuscript.

Major issues are listed below.
1. The manuscript is poorly organized. The text jumps from Figure 1 to Figure 3 before discussing Figure 2, making the paper difficult to read.

We thank the reviewer for highlighting this issue. This issue was also noted by Reviewer 2. We agree with both reviewers and have re-organised the manuscript so that the data is presented in the order it is described in the text.

- A schematic diagram of the tissue and cell types should be presented as Figure 1A to orient readers who might not work on this particular tissue.

Please show or schematize and provide references for the Gal4 expression patterns.

We thank the reviewer for this helpful recommendation. We have included a Figure panel 1A diagraming the midgut epithelium, its cell types, and cell type-specific GAL4 expression patterns. We have also included references for each GAL4 expression pattern/system at first mention in the results section.

2. The effect of reducing Drpr, Src, or Shark on the STAT transcriptional reporter damage response seems to be about two-fold. Is that sufficient to explain the effects on ISC proliferation? Do STAT heterozygotes have an effect? Does hyperactivation of STAT have an

effect?

We thank the reviewer for these questions. We realised that we had unfortunately made an error in calculating the % decreases throughout the manuscript. We apologise for this oversight, and these have now been corrected. We observe a greater than 50% decrease in Stat activity after *P.e.* infection with the loss of Draper (66.6%), Src42A (68.7%) and Shark (74.9%) in progenitors. Further, we find that ISC proliferation is also decreased by greater than 50% after *P.e.* infection with the loss of Draper (58.1%), Src42A (66.7%) and Shark (55.6%) in progenitors. Thus, we do believe that such a strong reduction in Stat activity could explain the effects we observe on ISC proliferation. This is consistent with previous work showing that depleting Stat in progenitors with RNAi blocks ISC proliferation after *P.e.* infection (Jiang et al., 2009, Cell; Buchon et al., 2009, Genes and Dev). In contrast, the hyperactivation of Stat by expressing hop or activated Jak Kinase (Hop^{TUM}) with esg^{ts} has been shown to induce ISC proliferation (Beebe et al., 2010; Buchon et al., 2009, Genes and Dev; Jiang et al., 2009, Cell). Since Stat activity would not likely reduce more than 50% in a Stat heterozygotes, we no longer see a benefit to performing this experiment.

3. The authors assume that Draper's function is recognizing PS on apoptotic cells and activating Src as it does in other contexts, but they should test this by addressing the following questions.

Phosphatidylserine (PS) is one of the most well-characterised ligands for Draper (Tung et al., 2013; Melcarne et al., 2019). Based on this, we indeed proposed that Draper is recognising PS on apoptotic midgut epithelial cells, leading to phosphorylation of its ITAM by Src and activation of Draper. We apologise for any confusion.

We were indeed curious to know whether PS is the ligand on apoptotic ECs for Draper found on progenitors after *P.e.* infection. To test whether PS plays a role in midgut regeneration, we examined ISC proliferation after expressing milk fat globule EGF-8 in progenitors with esg^{ts}. Milk fat globule EGF-8 is known to bind to PS (Tung et al, 2013; Hakim-Mishnaevski et al., 2019, Cell Reports), and if PS is involved, we expect to see reduced ISC proliferation. We found that expressing milk fat globule EGF-8 in progenitors blocked ISC proliferation in infected midguts by 53.7%. This suggests that the recognition of externalised phosphatidylserine on damaged enterocytes by ISCs is required for their proliferation (pooled data from 2 experiments shown below).

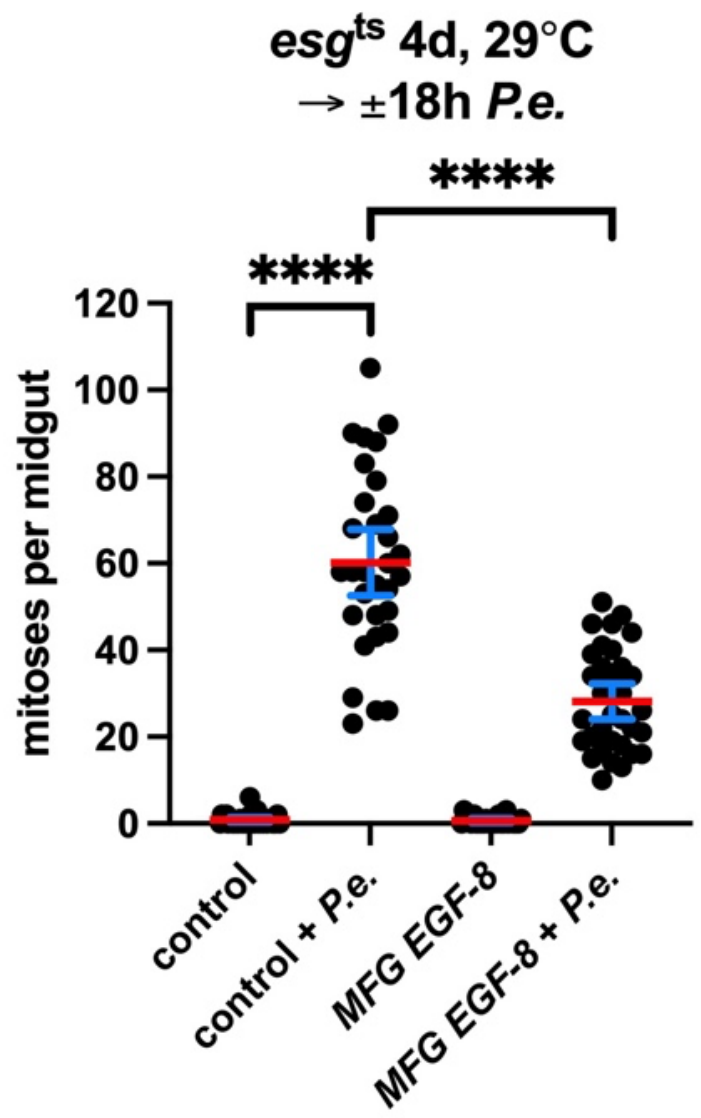

Moreover, we have tested whether another Draper ligand, Pretaporter (Kuraishi et al., 2009, EMBO Journal), is required in ECs to promote ISC proliferation after midgut damage. We found that depletion of *pretaporter* in ECs did not block ISC proliferation after *P.e.* infection, suggesting it is not the ligand for Draper in this context (1 of 2 experiments shown below).

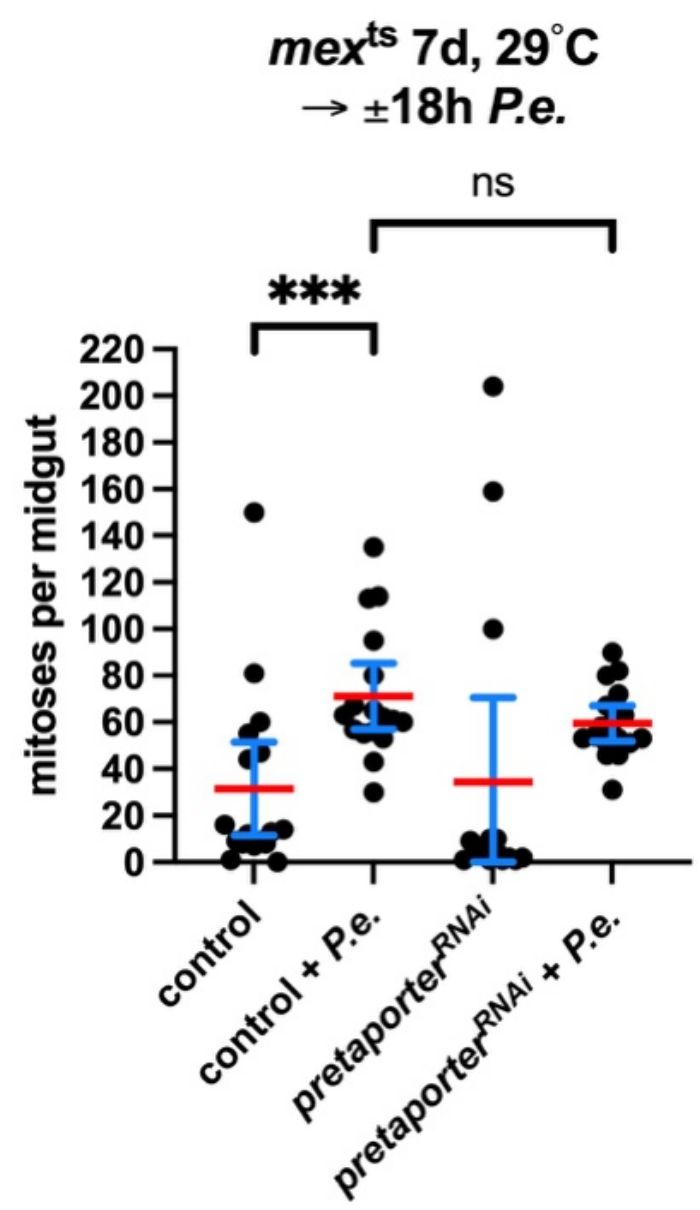

Further, we believe there may be a misunderstanding. As described above, we don't assume that Draper is activating Src42A at the molecular level, if this is what the reviewer suggests, and as far as we know Draper is not thought to activate Src42A in other contexts. We do, however, propose that Src42A is regulating Draper by phosphorylation of its ITAM, as has been shown in other contexts (Ziegenfuss et al., 2008, Nature; Williamson and Vale, 2018, J. of Cell Biology). We elaborate further on this below (see point b). Moreover, we will be testing whether Src42A is

required for ISC proliferation after EC ablation, which will activate Draper on progenitors (see Reviewer 3, point 3).

a. Does the p35 expression in EC also block pSrc in the progenitors? This would help connect apoptosis to Drpr-Src-Shark signaling.

We thank the reviewer for this interesting question. We have included the pSrc stainings in Fig. 1A-C mainly to localise Src activity within progenitors after *P.e.* infection. We show that pSrc is localised cortically in progenitors after *P.e.* infection, where the transmembrane receptor, Draper is usually found. This supports our model that pSrc activates Draper upon midgut damage. Based on previous work (Cordero et al., 2012, EMBO Journal; Perea et al., 2017, EMBO Journal), Src is activated by Wg and Ret signalling in progenitors after midgut damage. Whilst a general increase in epithelial pSrc has been reported after pathogenic bacterial infection (Cordero et al., 2014, EMBO Journal; Houtz et al., 2017), it is not clear whether EC apoptosis causes an increase in pSrc levels in midgut progenitors, and we agree that this would be an interesting observation that would strengthen our model.

Unfortunately, however, we are currently unable to perform further pSrc experiments, as the pSrc antibody we used does not work for us anymore. We have heard from colleagues that this happens commonly with pSrc antibodies. Also, please note that detecting pSrc in the midgut by using this antibody for human pSrc may vary (compare our staining with Cordero et al., 2014, EMBO Journal; Houtz et al., 2017; Perea et al., 2017). When it did work, we found a highly reproducible increase (n=3 experiments) in cortical pSrc in progenitors after infection. We also found it to be specific for phosphorylated Src42A (see Supplementary Fig. 1A-E). We have identified and ordered another mammalian pSrc antibody that has been reported to work on *Drosophila* tissue, which we will use to attempt to answer this question.

b. How does Drpr RNAi affect pSrc in progenitors?

We thank the reviewer for this suggestion. Based on previous work (Cordero et al., 2012, EMBO Journal; Perea et al., 2017, EMBO Journal), Src is activated in progenitors by Wg and Ret signalling after midgut damage. Src family kinases are known to phosphorylate ITAMs on immunoreceptors such as Draper and activate Syk (Shark) signalling (Ziegenfuss et al., 2008, Nature; Williamson and Vale, 2018, J. of Cell Biology; Mocsai et al., 2010). Williamson and Vale showed that ITAM phosphorylation occurs by exclusion of a transmembrane phosphatase that allows Src42A to phosphorylate the Draper ITAM. According to this model, Draper does not need to affect Src42A activity to become activated. To make this clearer, we will include a discussion about Draper activation in the Discussion.

c. Is PS recognition required or the domain that recognizes it on Drpr required? Is the ITAM domain of Drpr required?

We thank the reviewer for this suggestion, and we have performed experiments to test if PS recognition is required for ISC proliferation. As discussed above, we have tested this by expressing milk fat globule-EGF-8-GFP in progenitors with esg^{ts} in infected midguts and found that this blocks ISC proliferation.

It is well established that the ITAM domain is required for Draper signalling to recruit Shark (Ziegenfuss et al. 2008, Nature; Williamson and Vale, 2018, J. of Cell Biology), thus we don't feel that this needs to be further re-tested in midgut progenitors.

4. The claim that Draper serves a function other than stimulating engulfment requires stronger experimental support. Currently this interpretation rests solely on a negative result (absence of beta-gal-expressing nuclei within ISCs). It is possible that the apoptotic cells are extruded and are therefore in another focal plane. They may be engulfed and degraded rapidly and therefore not accumulating. The progenitor cells could be nibbling but not engulfing entire cells, so the authors should test that by labeling membranes of the target cells. The authors should demonstrate that they actually can detect engulfment by including a positive control. Live imaging also would be helpful to see where the apoptotic cells are going if they are not engulfed.

We thank the reviewer for these suggestions.

We would like to note that we have recently expressed a dominant-negative Rac (Rac^{N17}) in progenitors of infected midguts, which blocks their engulfment. This is due to Rac being required for actin polymerization for engulfment. In *Drosophila* egg chambers, Draper is required for Rac^{V12}-driven engulfment of nurse cells by border cells (Mishra et al., 2023, PNAS). Further, Rac^{N17} expression blocked Stat activation in glia after axonal injury of olfactory receptor neurons (Doherty et al., 2014, PLOS Biology). We found that Rac^{N17} expression in progenitors of infected midguts—blocking their engulfment—did not block Stat activity. These data suggest that engulfment is not required in progenitors for Stat activation after midgut damage, whereas Draper is, further supporting our model. We will include these data in our revised manuscript (1 of 2 experiments shown below).

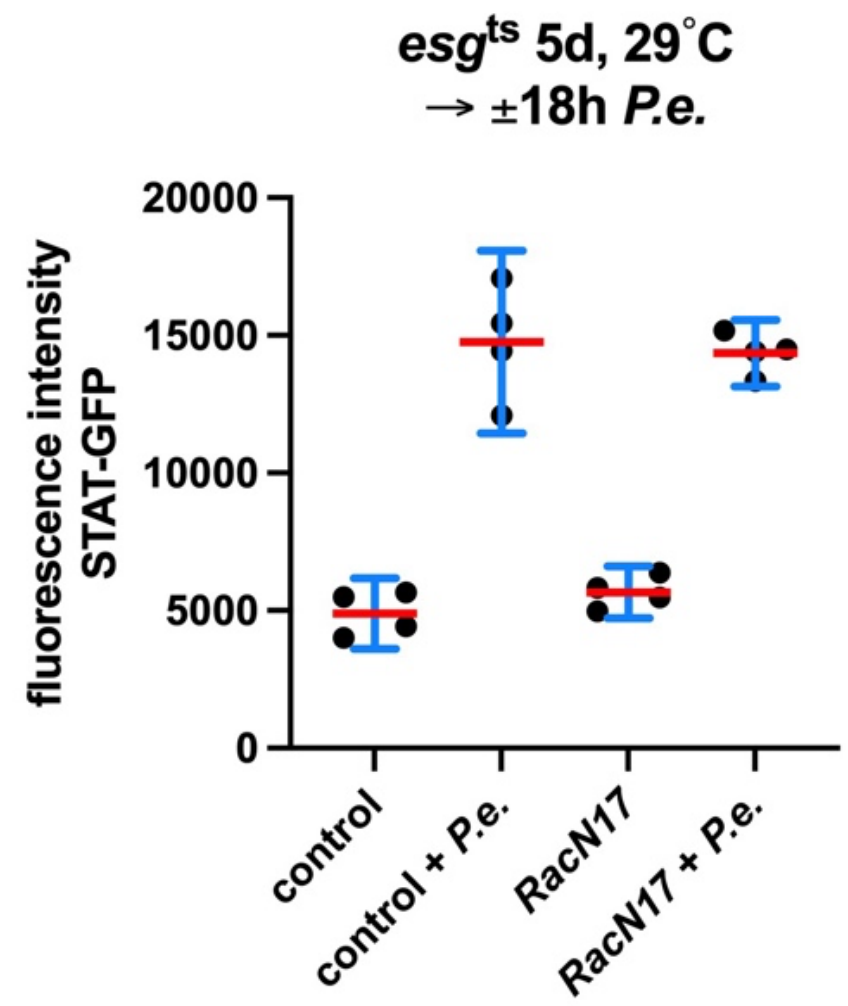

As mentioned in the Discussion, we agree that it is possible that ECs may be extruded from the epithelium and not engulfed. We will examine this by looking for fluorescent-labeled ECs in the lumen after infection. Unfortunately, we do not have live imaging set up in our lab. Whilst some studies suggest that ECs undergo apoptosis after being shed into the lumen after infection (Buchon et al., 2010, BMC Biol; Zhai et al., 2018, Immunity), others have shown dying or apoptotic ECs within the epithelium after infection (Buchon et al., 2009, Genes Dev; Loudhaief et al., 2017, Development). Please note that detecting dying (or apoptotic) cells within the midgut epithelium, particularly after midgut damage, is challenging (Nishida et al., 2024, Biol Open). Only a few papers have reported detecting apoptosis in the midgut epithelium after damage.

We agree that it is possible that ECs may be engulfed and degraded rapidly, and thus our assay may not be able to detect engulfed and degraded ECs. To test this, we will block the fusion of maturing phagosomes with the lysosome by blocking Rab7 function in progenitors of infected midguts and examine internalisation of ECs marked with ß-gal (Myo1A-lacZ).

Further, we agree that it is possible the progenitors may be nibbling on dying ECs (trogocytosis). To test this, we will express a membrane-localised fluorescent protein in ECs and determine if it is engulfed within progenitors.

If we are unable to detect engulfment by progenitors after *P.e.* infection, we will attempt to provide a positive control that demonstrates our ability to detect engulfment. For example, we could express constitutively active Rac in border cells in *Drosophila* egg chambers and look for polar cell engulfment as described in Misra et al., 2023, PNAS. Please note that this is a tissue we are not familiar with, and polar cell engulfment may be a rare event. As such, we are uncertain about the likelihood of success and would appreciate any suggestions you may have on how to establish a suitable positive control.

5. No data are provided to support the claim that Draper-Src-Shark-STAT signaling operates independently of the cytokines (Upd1-3) and their receptor Domeless. It would be relatively straightforward to look at Upd expression or test the effects of expressing dominant-negative Domeless in specific cell subsets (ISCs and VMs) using available Gal4 drivers.

We thank the reviewer for this suggestion. We agree that it is possible that Stat activity could increase in progenitors and VM due to the upregulation of Upds1-3. Even if this is the case, this would be the first mechanism (Draper-Src42A-Shark) described in progenitors that stimulates cytokine production after midgut damage (see Introduction, Zhang and Edgar, 2022; Zhou and Boutros, 2023).

To show that Draper-Src-Shark signalling regulates Stat independently of cytokines (Upd1-3), we will perform qPCR for Upd1-3 expression in infected midguts with reduced Draper-Src-Shark signalling in progenitors. If we observe similar levels of Upd1-3 induction in these midguts as in control, this will demonstrate that Stat is regulated by Draper-Src-Shark signalling despite cytokine induction in the regenerative niche. As blocking Draper-Src-Shark signalling only partially affects Stat activity, we believe that Upd/Jak signalling may also co-regulate Stat activity in progenitors after *P.e.* infection.

We found that expression of constitutively active Src42A in progenitors stimulates ISC proliferation, partially through Shark. If Draper-Src-Shark signalling induces cytokine production, then constitutively active Src42A expression in progenitors should induce cytokine production. However, the co-expression of dominant negative Domeless or *domeless*^{RNAi} did not block the effects of constitutively active Src42A expression on ISC proliferation (Cordero et al., 2014, EMBO Journal; Kohlmaier et al., 2015, Oncogene). These data indicate that Draper-Shark-Src signalling promotes ISC proliferation independent of cytokines.

Unfortunately, currently it is technically not possible for us to overexpress constitutively active Src42A in progenitors and simultaneously deplete *domeless* in the VM. JAK-Stat activity in VM stimulates EGF (Vein) production, which stimulates ISC proliferation (Jiang et al., 2011, Cell Stem Cell; Zhou et al., 2013, Dev Biol). It has been shown that depleting *domeless* in the VM reduces ISC proliferation (Zhou et al., 2013) after infection. We will include this in the Discussion.

6. No data are provided to support the claim that phosphatidylserine on the membrane of apoptotic enterocytes activates Draper/Src/Shark signaling.

As mentioned above, we only propose that phosphatidylserine (PS) activates Draper-Src-Shark signalling. However, as described above, we have now tested this by expressing milk fat globule-EGF-8-GFP in progenitors with *esg*^{ts} in infected midguts and found that this blocks ISC proliferation.

7. No mechanism is tested or proposed for how Src activates STAT.

We have proposed that Src can activate Stat via Draper and Shark based on their similar effects on Stat activity in progenitors and the VM after *P.e.* infection. Several studies have shown that immunoreceptors such as Draper and the tyrosine kinase Syk (or Shark) are activated by Src family kinases (Ziegenfuss et al 2008, Nature; Williamson and Vale, 2018, J. Cell Biology; Mocsai et al., 2010), and that this signalling module can activate Stat (Doherty et al., 2014, PLOS Biology). Moreover, Stat activity promotes ISC proliferation after midgut damage (Jiang et al., 2009, Cell; Buchon et al., 2009, Genes Dev), and we have also shown that Shark is required for ISC proliferation due to constitutively active Src42A expression in progenitors. Furthermore, Syk has been shown to directly phosphorylate and activate Stat independent of the cytokines or JAK signalling (Liu et al., 2021, Cell Reports; Uckun et al., 2010, PNAS). Thus, Shark may be directly regulating Stat in progenitors after midgut damage. We are proposing this possible mechanism in the revised discussion.

8. The non-autonomous effect is surprising but unexplored. A potential mechanism that would explain both how Draper-Src-Shark activates STAT in the ISC and in the muscle would be if Upds are secreted, which is a well-established effect of injury.

We thank the reviewer for this suggestion. We have included Stat-GFP effects on VM because it is intriguing and for completeness, as Stat-GFP is also induced in the VM after *P.e.* infection. We feel that determining how progenitors could regulate the VM is out of scope for this study; however, we will determine if Upd expression could explain these effects. As described above, we will test if Upd1-3 induction after *P.e.* infection is affected in midguts when Draper-Src-Shark signalling is blocked within progenitors.

Minor issues

1. ISC is not defined when first mentioned

We apologise for this oversight, and we have added this to our revised summary.

2. Figure 1D-G, show representative images along with quantifications.
Why are the scales different on the different STAT graphs? makes it hard to compare between the figures.
Why are axes different numbers in 1F ?

The height of the y-axis in 1F is different to improve the visibility of the data. For the STAT graphs: 1) the scales on the y-axis are every 5 progenitors for all STAT graphs, but the scales vary for the x-axis in Figure 2 (every 2000 in Fig. 2 vs 5000 in Figs. 3 and 4) because the Stat activity was induced less in this experiment. These data can now be found in Supplemental Fig. 4.

3. Please move Supplemental figures 1C, 2C 3C into the main figures

We have moved Supplemental Figs. 1C, 2C and 3C to the main figures (Fig. 3) in our revised manuscript.

Reviewer #1 (Significance (Required)):

This work in principle provides a conceptual advance in stem cell biology and regeneration by proposing a new function for the molecular machinery best known for innate immune responses: specifically that the Draper - Src - Shark pathway that is best characterized in phagocytosis plays a role in stimulating STAT upon tissue damage. This work will be of interest to basic scientists studying stem cell responses to tissue injury. It has relevance for tissue repair, regeneration, and aging. The limitations of the study are described above and include the confusing presentation as well as missing controls and insufficient data to support some of the conclusions.

Reviewer 2

Reviewer #2 (Evidence, reproducibility and clarity (Required)):

Damage recognition by intestinal stem cells via Draper-Src-Shark-STAT signalling promotes adult Drosophila midgut regeneration.

In this manuscript, Legido et al present evidence that the Src42A kinase in Drosophila is essential for epithelial regeneration in damaged fly midguts. The work is performed to a very high standard; images are excellent throughout; statistics are appropriate, and sample sizes are high; the authors clarify size effects throughout their work; all data are easily followed and appropriately interpreted.

As a result, I am highly enthusiastic about the value and quality of this study and commend the authors for their work. Most of my concerns are relatively minor and are detailed below.

We thank the reviewer for this positive evaluation of our work. We are delighted to hear that the reviewer is highly enthusiastic about the value and quality of our study.

MAJOR ISSUES

1. Src as a bacteria sensor. I struggle with the rationale behind Figure 1E. The authors claim these data exclude possible roles for Src as a molecule that "detects pathogenic bacteria". I may be incorrect, but it appears the experiment is intended to test if Src is a classical pattern recognition receptor (PRR) that senses microbial patterns. It's unclear what would lead to this hypothesis, as Src does not have the molecular structure or prior literature we would expect to see in PRRs. Nonetheless, I do not agree that the results exclude roles for Src in sensing

bacterial patterns (regardless of how unlikely the possibility is). The experiment in Figure 1E was performed with conventional flies with a conventional microbiome, so we cannot formally exclude the possibility that Src "senses" dysbiotic commensal bacterial patterns in an SDS-treated gut. To exclude this possibility, it would be important to repeat Figure 1E in axenic flies. For me, the data in Figure E are valuable, but primarily because they demonstrate that Src is also capable of initiating repair in response to chemical stress, suggesting that the phenotype is not exclusive to a unique feature of Pe infection. If the authors wish to exclude roles for Src as a PRR, they should be encouraged to stress axenic guts. As it is highly unlikely that Src is a PRR, I feel the authors would be best advised to consider reorganizing the text to clarify that their work extends prior observations by showing that Src also promotes renewal in response to a chemical stress.

We thank the reviewer for highlighting this issue. It was not our aim to determine whether Src acts as a pattern recognition receptor (PRR). We agree with the reviewer that this would require exposing axenic flies to detergent (SDS) stress to rule out detection of dysbiotic commensal bacteria. We will edit the text to point out that Src is required for ISC-mediated regeneration in response to damage caused by both pathogenic bacteria and chemicals (detergents).

2. Possible involvement of the UPD/Domeless pathway. At times, it appears that the authors argue their data exclude roles for UPD signaling in STAT activation by Src (e.g. penultimate sentence of Introduction, relevant text in Results section, paragraph of Discussion). The authors are correct that prior studies argue against Domeless involvement, but none of the experiments in this manuscript test that hypothesis further. As a result, I encourage the authors to be more precise in their discussion of Upd signaling.

We thank the reviewer for pointing this out, and we agree completely. Since blocking Draper-Src-Shark signalling only partially affects Stat activity, we believe that Upd/JAK signalling may also co-regulate Stat activity in progenitors after midgut damage. We will edit the text and Figure 7 to reflect this possibility. Further, to test if STAT is activated by Draper through Upd production, we will perform qPCR for Upd1-3 induction in infected midguts with progenitors depleted for Draper-Src-Shark signalling.

MINOR ISSUES

1. Regenerative Niche: I struggle with the concept of a regenerative niche in the fly gut. As the authors point out essentially all intestinal cells (enteroblasts, enterocytes, enteroendocrine, muscle, trachea) influence stem cell behaviour in the fly gut. As a result, it's difficult to agree that there is a subset of specialist "niche" cells that specifically regulate stem cell proliferation in the fly midgut. I find the term "microenvironment" (used in the Introduction) more appropriate for flies.

We thank the reviewer for their suggestion to use “microenvironment” over “niche”. We agree that there is not a subset of specialist “niche” cells that specifically regulate ISC proliferation during midgut regeneration. We use the term “niche” more broadly here and synonymous with

“microenvironment”. Further, we qualify this niche as a “regenerative” niche as opposed to a “stem cell” niche. For example, this review on mammalian intestinal regeneration (Palikuqi et al., 2022) uses both terms interchangeably. We will define upfront that the regenerative niche refers to a regenerative microenvironment.

2. This sentence from the Introduction requires modification: "Mechanistically, Draper-Src-Shark signalling stimulates ISC proliferation by increasing STAT activity in ISCs, which has previously been thought to be exclusively activated by cytokine (Upd1-3)/JAK signalling." As the authors point out at an earlier stage, prior work had already established that Upd1-3 are not needed for Src-dependent STAT activation: "Domeless and its ligands, Upd1-3, are not required for Src42A to stimulate ISC proliferation through STAT activation".

We thank the reviewer for pointing this out. Previous work showed that Stat is required for constitutively active Src42A to promote ISC proliferation in homeostatic midguts (Cordero et al., 2014, EMBO Journal; Kohlmaier et al., 2015, Oncogene). Homeostatic midguts have low levels of Upd1-3, but these are upregulated upon infection (Jiang et al., 2009, Cell; Buchon et al., 2009, Genes Dev). Further, these studies did not directly investigate whether Src42A regulates Stat activity but only its requirement after midgut damage (Cordero et al., 2014, EMBO Journal; Kohlmaier et al., 2015, Oncogene). Thus, until now, Stat activation after midgut damage has been thought to be exclusively activated by cytokine/JAK signalling (See the following reviews: Herrera and Bach, 2019; Zhang and Edgar, 2022; Zhou and Boutros, 2023). We will modify the text to clarify this.

3. Methods: More detail is needed for the infection protocol. The authors should detail their handling of Pe (growth medium; all steps taken to centrifuge, resuspend, etc. Pe prior to infection; definition of 20X Pe ideally as CFU/ml, or OD). Otherwise, it will be very difficult for others to follow up on this study. Also, please clarify if females used in these experiments were mated or virgin.

We thank the reviewer for helpful recommendation. We have included more detail about our infection protocol to improve the reproducibility of our experiments. In this study, we used mostly virgin females, but we have performed a few experiments with mated females. We have indicated which experiments used mated females in our methods section. We find that the mean number of mitoses (proliferating ISCs) per midgut is similar between mated and virgin females.

4. Quantifications: When introducing data in Figure 3, please consider including one or two sentences explaining what we are looking at in panels L-O. For many within the field, it is likely intuitive, but I wonder if those less familiar with Drosophila will follow along.

We thank the reviewer for highlighting this issue. We apologise for not making this clear in our manuscript. In Figure 3, Panels L-O are histograms of Stat-GFP levels in progenitor cells of uninfected control midguts (L), *P.e.*-infected control midguts (M), control midguts expressing

shark^{RNAi} in progenitors (N) and *P.e.*-infected midguts expressing *shark^{RNAi}* in progenitors (O). We will describe better what these data are in the Results section.

5. Organization of data. For now, the arrangement of the figures is slightly confusing, and seldom aligns with the text. I find this diminishes the quality of the manuscript. I recommend the authors consider rearranging all figures to directly match the text. For example, STAT:GFP images and quantifications are discussed in the section "Draper-Src-Shark signalling promotes ISC proliferation through STAT activation". It would make most sense for these data to all exist in a single matching figure. These issues apply throughout (Figure 3 and 4 are discussed prior to Figure 2, text for Figure 5 follows text for figure 6, etc.). This does not require any additional experiments but would make the text much more accessible to readers.

We thank the reviewer for this helpful suggestion. We completely agree, and we will improve organisation of the data by rearranging the figures, so they align with the text.

6. Figure 7: I appreciate the inclusion of a speculative model in Figure 7. However, I feel the accompanying text is more appropriate for the discussion than the result, and there are cases where the wording could be moderate (e.g. "This allows cortically-localised activated Src to phosphorylate the ITAMs on Draper" is not supported by data demonstrating phosphorylation of ITAMs on Draper by Src).

We thank the reviewer for pointing this out. We agree that this text describes our speculation of how Draper-Src-Shark signalling functions in progenitors after midgut damage and belongs in the Discussion. Our speculation is based on a number of studies describing immunoreceptor-Src family kinase-Syk/Shark signalling in both *Drosophila* and humans (Ziegenfuss et al., 2008, Nature; Williamson and Vale, 2018, J. of Cell Biology; Mocsai et al., 2010).

7. Returning to the theme of Src as a potential PRR, the following sentence from the discussion confuses me: "This suggests that Src42A in ISCs senses tissue damage rather than pathogenic bacteria themselves, possibly through the recognition of pathogen-associated molecular patterns (PAMPs)." Here, the authors appear to suggest that Src detects PAMPs. Is that correct? The hypothesis is not supported by the accompanying data. Presumably they are arguing that Src does not detect PAMP. If so, the text could be clarified. Additionally, I would encourage the authors to consider microbe-associated molecular pattern (MAMP) over PAMP.

We thank the reviewer for these suggestions. We apologise for the confusion. Our aim was to highlight that Src senses tissue damage. Our data shows that Src42A is required in progenitors for the ISC response to tissue damage caused by both pathogenic bacteria and detergents. We will modify the text to reflect this better.

Reviewer #2 (Significance (Required)):

The data are consistent with earlier reports by Cordero et al (EMBO 2014), and Kohlmaier et al.

(Oncogene 2015). However, this study goes further by presenting two important discoveries. First the authors present convincing genetic evidence that Src-dependent regeneration involves participation of Draper and Shark. Secondly, the authors provide data that indicate Draper initiates Src signaling in a phagocytosis-independent manner. As a result, the work constitutes an important extension of previous work and adds to our understanding of epithelial regeneration in fly guts. The exact mechanism by which dying enterocytes activate Draper in ISCs requires clarification, and possible involvement of visceral muscle hinted at in figure six also requires clarification. However, the current study will doubtlessly be of interest to researchers using flies to characterize epithelial cell dynamics during periods of stress and should be of appeal to a larger community interested in epithelial regeneration. I

We thank the reviewer for this positive evaluation and for highlighting our two important discoveries: 1) Src-dependent regeneration requires Draper and Shark, and 2) Draper-Src-Shark signalling can promote regeneration in a phagocytosis-independent manner.

As discussed above, we have clarified how dying enterocytes activate Draper in ISCs by examining one of the ligands that activate Draper, phosphatidylserine (PS). We found that masking externalised phosphatidylserine on dying ECs by expressing milk fat globulin EGF-8 reduced ISC proliferation in infected midguts.

As for the possible involvement of the visceral muscle, we currently believe that increased Stat activity in the VM will stimulate EGF production (Vein) that will further boost ISC proliferation as proposed by Jiang et al., 2011, Cell Stem Cell; Zhou et al., 2013, Dev Biol. We will include some text in the Discussion describing this work and placing it into the context of our work.

Reviewer 3

Reviewer #3 (Evidence, reproducibility and clarity (Required)):

In this manuscript, Legido et al. investigate the role of Draper-Src-Shark-STAT signaling in regulating intestinal stem cell (ISC) proliferation upon bacterial infection using the Drosophila intestine model. They demonstrate that Draper-Src-Shark signaling acts in ISCs to recognize midgut damage and is required for ISC proliferation following bacterial infection. In addition, they show that Draper-Src-Shark signaling regulates STAT activation both in progenitor cells and the visceral muscle.

Major concerns:

1. While the authors use Mex-Gal4 to drive gene expression in Ecs and examine pSrc, it would be better to co-express UAS-GFP to clearly mark Ecs.

We thank the reviewer for this suggestion. We agree that this would be ideal. The differentiated cells in the epithelium include ECs and EEs. The ECs are much larger than EEs and can be further identified by their large polyploid nucleus. We have identified ECs here using these two

criteria. Unfortunately, we currently cannot repeat this experiment with marked ECs because the pSrc antibody we used does not work for us anymore, which appears to be a common problem with pSrc antibodies. As mentioned above, we are currently testing another mammalian pSrc antibody that has been reported to work on *Drosophila* tissues. The antibody we used previously was also used in Cordero et al., 2014 (EMBO Journal) and Perea et al., 2017 (EMBO Journal). As Draper is a transmembrane immunoreceptor located at the plasma membrane, we wanted to show where activated Src (pSrc) is localised after *P.e.* infection. We found that pSrc is localised cortically in progenitors after *P.e.* infection, where it could phosphorylate the ITAM within Draper.

2. It is difficult to see the upregulation of pSrc in ECs (Supplementary Fig. 1D-G'). To confirm the specificity of the pSrc antibody, the authors should knock down Src42 using esg-Gal4 during P.e. infection and examine the results.

We thank the reviewer for this suggestion. Unfortunately, the pSrc antibody we were using is not working for us anymore, thus we currently cannot repeat this experiment. We have included arrows where there are changes in pSrc after infection in ECs, and its down regulation after depletion of Src42A in ECs. We have now included a quantitation of pSrc levels from the whole epithelium, which is about 90% ECs, showing that pSrc levels increase with infection but decrease in midguts with ECs depleted for Src42A. While these measurements are not EC-specific, these data suggest that Src42A increases in the midgut epithelium after infection and that the antibody is specific for Src42A phosphorylation. Please note that several studies have used this pSrc antibody in the midgut but never tested its specificity (Cordero et al., 2014, EMBO Journal; Perea et al., 2017, EMBO Journal; Houtz et al., 2017, PLOS Genetics).

3. Although the authors show that Shark is required for Src42A-mediated ISC proliferation, they should also test whether Src42A is required for Draper-induced ISC proliferation to complete the signaling pathway analysis.

We thank the reviewer for this suggestion. Based on the literature, we expect Src family kinases to phosphorylate the ITAM within immunoreceptors such as Draper to allow Shark/Syk signalling (Ziegenfuss et al., 2008; Williamson and Vale, 2018, J. of Cell Biology; Mocsai et al., 2010). Thus, it is highly likely that Src42A is required for Draper signalling.

Based on our study and prior work, we can only activate Draper by expressing constitutively active Src42A, by inducing apoptosis in ECs or by *P.e.* infection. We have already shown that, like Draper, Src42A is required in progenitors for ISC proliferation and Stat activation after *P.e.* infection. Thus, we plan to examine ISC proliferation in Src42A heterozygous midguts expressing *reaper* in ECs, inducing EC apoptosis and thus activating Draper. However, it is currently difficult for us to genetically manipulate two cell types simultaneously in the midgut. We plan to try using a combined GAL4 driver *esg*^{ts} *Myo1A*^{ts} to express *reaper* and *Src42A*^{RNAi} in both progenitors and ECs as it was previously reported that *reaper* expression is unable to kill progenitors (Jin et al., 2017, Stem Cell Reports). Thus, we should be able to express *Src42A*^{RNAi}

within progenitors for long enough to examine an effect on ISC proliferation due to EC apoptosis.

4. The authors conclude that "these data indicate that Draper-Src-Shark signaling promotes ISC proliferation through STAT activation." However, the current data only show that STAT activation upon P.e. infection depends on Draper-Src-Shark signaling. To strengthen this conclusion, the authors should activate Draper-Src-Shark signaling and determine whether STAT knockdown can rescue the ISC proliferation phenotype.

We thank the reviewer for this suggestion. Stat has been shown to be essential in progenitors for ISC proliferation after midgut damage (Jiang et al., 2009, Cell; Buchon et al., 2009, Genes Dev). Furthermore, both Cordero et al., 2014 and Kohlmaier et al., 2015 showed that Stat is required for ISC proliferation caused by expression of constitutively active Src42A in progenitors. As mentioned above, we can only activate Draper-Src-Shark signalling by expressing constitutively active Src42A, *P.e.* infection and by expressing pro-apoptotic factors in ECs. Thus, we will examine ISC proliferation in Stat heterozygous midguts expressing *reaper* in ECs.

5. The authors state that "p35 expression in ECs partially blocked ISC proliferation by 40.1% in P.e.-infected midguts compared to controls (Fig. 5A), suggesting that some infected ECs undergo apoptosis." However, they should directly examine apoptosis markers in response to P.e. infection to support this claim.

We thank the reviewer for this suggestion. Examining apoptosis in the midgut after damage, especially after infection, has been notoriously challenging (Nishida et al., 2024). This is despite that p35 can block ISC proliferation after *P.e.* infection (this study and Phillips et al. DOI: https://doi.org/10.1101/2025.04.16.649133) and DNA-damage (bleomycin) (Amcheslavsky et al., 2009). We have tried to directly examine apoptosis in the midgut epithelium after *P.e.* infection (cleaved-PARP) but were unsuccessful. Nevertheless, we will try other methods to detect apoptosis and necrosis using anti-dcp-1 and Sytox (Kolahgar et al., 2015) for the revision. As a positive control for apoptosis, we will induce apoptosis in ECs by expressing *reaper*.

In support of our data suggesting that EC apoptosis is required for ISC proliferation, we recently found that overexpression of the anti-apoptotic factor, DIAP1, in ECs blocked ISC proliferation in *P.e.*-infected midguts by 57.8% (pooled data from 2 experiments shown below). Taken together with our data suggesting a role for externalised phosphatidylserine on ECs for ISC proliferation after infection, these data strongly suggest a role for apoptosis recognition in ISC proliferation after midgut damage.

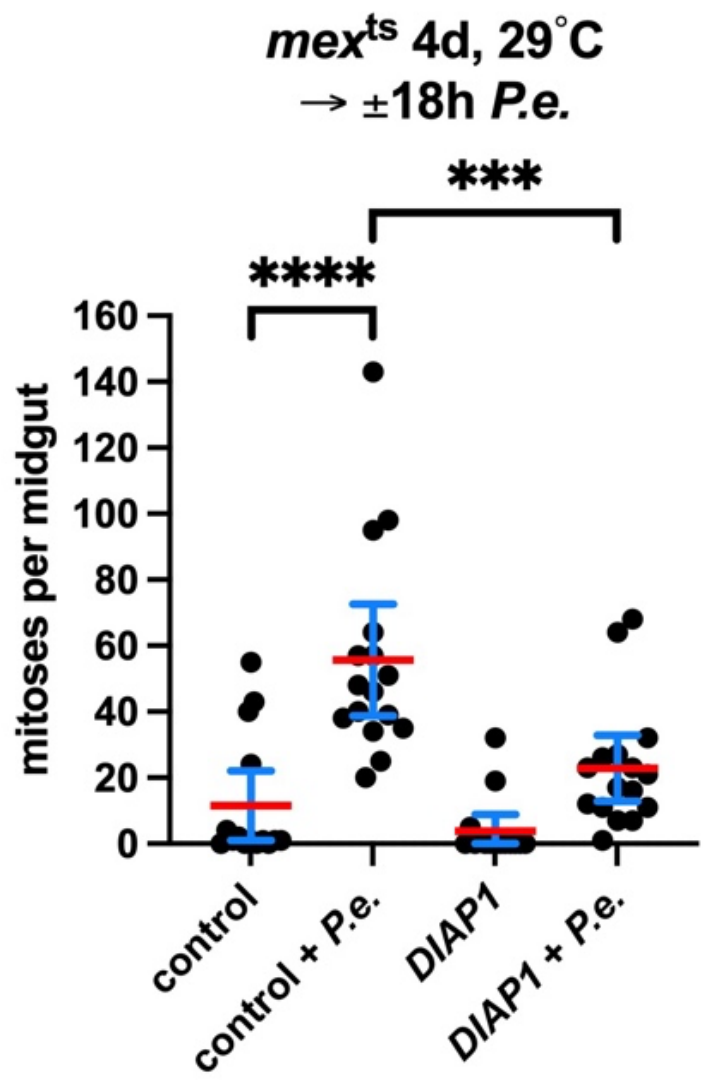

Phosphatidylserine is also exposed during necrosis. Thus, we also examined necrosis after *P.e.* infection using the in vivo necrosis-sensing tool, Necrosensor (Nishida et al., 2024). The Necrosensor was shown to detect necrosis in midguts exposed to bleomycin (Nishida et al., 2024). Unfortunately, we did not observe activation of the Necrosensor in the midgut epithelium after *P.e.* infection. This suggests that midgut epithelial cells are not undergoing necrosis after *P.e.* infection.

6. To further test the relationship between apoptosis and Draper-Src-Shark signaling, the authors should induce apoptosis in ECs while blocking Draper-Src-Shark signaling and assess whether ISC proliferation is rescued.

We thank the reviewer for this suggestion. As mentioned above, it is difficult for us to simultaneously manipulate both the ECs and progenitors. Thus, we will examine ISC proliferation in *draper* heterozygous midguts expressing *reaper* in ECs. We will also try to use the combined GAL4 driver *esg^{ts} Myo1A^{ts}* to express *reaper* and *shark^{RNAi}* or *draper^{RNAi}* in both progenitors and ECs.

7. The authors demonstrate that Draper-Src-Shark signaling in progenitor cells regulates STAT activity non-cell autonomously in the visceral muscle. To strengthen this conclusion, they should activate Draper-Src-Shark signaling specifically in progenitor cells and examine STAT-GFP levels in muscle cells.

We thank the reviewer for this suggestion. We will overexpress constitutively active Src42A in progenitors and examine its effects on Stat-GFP in the visceral muscle to test if Src42A activity in progenitors is sufficient to activate Stat in the VM.

Minor concerns:

• The authors claim that "Draper-Src-Shark signaling acts in ISCs to recognize midgut damage and is required for full STAT activation in ISCs to promote their proliferation." However, Draper-Src-Shark signaling does not seem to completely block STAT activation in response to P.e. infection. The authors should review the manuscript carefully and revise claims that are not fully supported by the experimental data.

We thank the reviewer for pointing this out. We completely agree and did not mean to imply that all Stat activation after *P.e.* infection is due to Draper-Src-Shark signalling. As STAT activation is only partially decreased by depleting Draper-Src-Shark signalling, we currently believe that Upd-Jak signalling partly regulates Stat activation after *P.e.* infection. We will modify the text and Figure 7 model to make this clearer.

Reviewer #3 (Significance (Required)):

Although the role of Src42A in ISC proliferation upon bacterial infection has been previously reported, the mechanism elucidated in this study provides new insights into intestinal stem cell regulation and could contribute meaningfully to the stem cell field.

We thank the reviewer for the positive evaluation and for pointing out that our "study provides new insights into intestinal stem cell regulation and could contribute meaningfully to the stem cell field".

3. Description of the revisions that have already been incorporated in the transferred manuscript

Please insert a point-by-point reply describing the revisions that were already carried out and included in the transferred manuscript. If no revisions have been carried out yet, please leave this section empty.

1. We have re-organised the manuscript so that the data is presented in the order it is described in the text. (Reviewers 1 and 2)
2. We have included Figure panel 1A diagraming the midgut epithelium, its cell types, and cell type-specific GAL4 expression patterns. (Reviewer 1)
3. We have included references for each GAL4 expression pattern/system at first mention in the Results section. (Reviewer 1)
4. We re-calculated our % decreases and have included the corrected values throughout our manuscript.
5. We have defined ISC at first mention in the summary (Reviewer 1)
6. We have moved Supplemental Figs. 1C, 2C and 3C to new main Fig. 3. (Reviewer 1)
7. We have edited the Results to point out that Src is required for ISC-mediated regeneration in response to damage caused by both pathogenic bacteria and chemicals (detergents). (Reviewer 2)

8. We have edited Fig. 7 panel B and B'. We have now included "niche signals" to panel 7B and "Upds" to panel 7B' to highlight that cytokines (Upds) may co-regulate Stat activity in progenitors after midgut damage. (Reviewers 2 and 3)
9. We have defined at first mention that the regenerative niche refers to a microenvironment. (Reviewer 2)
10. We have now included more detail about our infection protocol to improve reproducibility of our experiments. (Reviewer 2)
11. We have indicated in the methods sections that we have used virgin females in most of our experiments but have indicated experiments where mated females were used. (Reviewer 2)
12. We have removed from the Results section our speculation of how Draper-Src-Shark signalling functions in progenitors after midgut damage and have moved this to the Discussion. (Reviewer 2)
13. We have now included in Supplementary Fig. 1E quantitation of epithelial pSrc in 1) control midguts, 2) *P.e.* infected midguts, 3) midguts expressing *Src42A^{RNAi}* in ECs with *mex^{ts}* and 4) *P.e.*-infected midguts expressing *Src42A^{RNAi}* in ECs with *mex^{ts}*. This data shows that pSrc increases in the midgut epithelium after infection and that the antibody is specific for Src42A phosphorylation. We have also added new arrows (green) indicating pSrc on EC membranes (Supplementary Fig. 1B') and green asterisks * to indicate polyploid EC nuclei in Supplementary Fig. 1A-D. (Reviewer 3)
14. We have expanded our discussion of how phosphotidylserine-bound Draper forms microclusters in the plasma membrane, thus excluding a transmembrane phosphatase and allowing its activation by Src42A. As Wg and Ret signalling promotes Src42A activation after midgut damage, this would drive Draper towards a net activated state within microclusters. See Discussion. (Reviewer 1)
15. We have now discussed how STAT activation in the VM by Draper-Src-Shark signalling in progenitors could support midgut regeneration. We discuss that STAT activation in the visceral muscle stimulates EGF (Vein) production, thereby stimulating ISC proliferation. See Discussion. (Reviewer 1)
16. We have now proposed how Draper-Src-Shark signalling could activate Stat. We have included a discussion about how Shark activity in progenitors after infection could directly activate STAT, based on work in mammalian cells. See Discussion. (Reviewer 1)

4. Description of analyses that authors prefer not to carry out

Please include a point-by-point response explaining why some of the requested data or additional analyses <u>might not be necessary or cannot be provided within the scope of a revision</u>. This can be due to time or resource limitations or in case of disagreement about the necessity of such additional data given the scope of the study. Please leave empty if not applicable.

Analyses that we prefer not to carry out include:

1. Reviewer #1, point 2: Examining ISC proliferation in Stat heterozygotes after *P.e.* infection and examining the effects of Stat hyperactivation on ISC proliferation. The effects of the loss

of Draper-Src-Shark signalling on Stat activity is greater than 50%. It is unlikely that Stat activity decreases greater than 50% in Stat heterozygotes. Thus, we find that this experiment will not be very informative. Further, it has been already shown that depleting Stat in progenitors blocks ISC proliferation after infection (Jiang et al., 2009; Buchon et al., 2009). In addition, hyperactivation of Stat by expressing Jak (hop) or activated Jak (Hop^{TUM}) in progenitors has been already shown to induce ISC proliferation (Beebe et al., 2010; Buchon et al., 2009; Jiang et al., 2009).

2. Reviewer #1, point 3b: Determining whether *drpr* depletion in progenitors would affect pSrc. pSrc has been previously shown to be activated by Wg and Ret signalling after midgut damage (Cordero et al., 2014; Perea et al., 2017). Activation of Src by these pathways should be enough to activate Draper. Based on work by Williamson and Vale, 2018, Draper would not need to activate Src42A to become fully activated. Drpr microclustering after apoptosis and its exclusion of a transmembrane phosphatase should allow its net activation by Src42A.
3. Reviewer #1, point 3c: We will not examine whether the ITAM domain of Draper is required for ISC proliferation after damage. It is well-established that the ITAM domain is required for Draper signalling (Ziegenfuss et al., 2008; Williamson and Vale, 2018).
4. Reviewer #1, point 4: We will be unable to use live imaging to assess whether infected or dying ECs enter another plane in the epithelium or are shed into the lumen, since we do not have live imaging set up in our lab. However, we are planning to perform other experiments to address this point (see response to the reviewer).
5. Reviewer #3, point 2: We will not examine pSrc in GFP-marked ECs after infection nor will we examine specificity of the pSrc antibody by depleting *Src42A* from progenitors during *P.e.* infection. We have now included a quantitation of pSrc levels from the whole epithelium, which is about 90% ECs, showing that pSrc increases with infection but decreases in midguts with ECs depleted for *Src42A*. Moreover, this antibody has been used by two other studies, Cordero et al., 2014 (EMBO Journal) and Perea et al., 2017 (EMBO Journal).
6. Reviewer #3, points 3, 4, and 6 and Reviewer 2, points 4 and 5: We are currently unable to simultaneously manipulate genetically more than one midgut cell type. Thus, it will be difficult to activate Draper-Src-Shark signalling (by expressing constitutively active Src42A in progenitors or pro-apoptotic factors in ECs) and simultaneously block factors in progenitors (Src42A, Draper, Stat) or in the visceral muscle (Domeless). Establishing these expression systems to perform these experiments would take a considerable amount of time. We are however planning to take an alternative approach to address this point (see response to the reviewer). Similarly, it will be difficult for us to block lysosomal degradation in progenitors by inhibiting Rab7 and mark EC membranes to examine whether trogocytosed membranes are degraded.

1st Editorial Decision 9th Jul 2025

Dear Dr Patel,

Thank you for the submission of your amended manuscript (EMBOJ-2025-111711; RC-2025-02969) to The EMBO Journal together with the point-by-point response to the concerns raised during peer-review at Review Commons. We have carefully assessed your amended work and related revision plan in response to the experts. I am afraid in light of all information at hand decided that we cannot offer publication in The EMBO Journal.

We appreciate your results on a signaling axis involving Draper-Src-Shark in driving STAT-mediated intestinal stem cell proliferation upon various tissue damaging cues, as well as paracrine non-cell autonomous activation of visceral muscle. We note that these results will as such be of interest to the field However, we also noted that Src-STAT signaling has as such been linked to regulation of ISC proliferation in the fly. Also, Draper-Shark function in sensing and integrating tissue apoptosis has been reported. We see from your rebuttal response that you addressed a number of issues, including exclusion of cell engulfment as a Draper-dependent requirement in this context. Overall, however, I regret to say we concluded that the manuscript remains still doesn't provide the level of conceptual advance we need to request for the EMBO Journal.

I am sorry to disappoint you on this occasion and wish you swift success with the publication of this work. I again apologise for the protraction.

Best regards,

Daniel Klimmeck

Daniel Klimmeck, PhD
Senior Editor
The EMBO Journal

****
Rev_Com_number: RC-2025-02969
New_manu_number: EMBOJ-2025-121711-T
Corr_author: Patel
Title: Damage recognition by intestinal stem cells promotes adult Drosophila midgut regeneration.

Dear Daniel,

Thank you for considering our manuscript for publication in *The EMBO Journal*. We also appreciate the relatively prompt decision and the detailed explanation of the editorial outcome.

The editorial decision for our manuscript is disappointing, particularly because we were encouraged to submit based on fit and our reviews. Moreover, we disagree with the reasons provided that underpin the editorial decision.

One of the reasons provided was that "Src-STAT signaling has as such been linked to regulation of ISC proliferation in the fly". It is true that both Src42A and STAT are known to regulate ISC proliferation after midgut damage. However, it was not established that Src42A regulates Stat activity as we have shown in our study. Based on the experiments in Kohlmaier et al, 2015 (Oncogene) and Cordero et al, 2014 (EMBO Journal), STAT could simply be required for Src42A to promote ISC proliferation. Furthermore, it was already known that STAT and EGFR/ERK are required for ISC proliferation. These studies only discovered Src as a new factor that promotes ISC proliferation after midgut damage, possibly through STAT.

https://www.embopress.org/doi/full/10.1002/embj.201387454

https://www.nature.com/articles/onc2014163

Perea et al., 2017 (EMBO Journal) suggests that Ret signalling promotes ISC proliferation through Wingless (Wg) and Src after midgut damage. Similarly, Src and Wg (Cordero et al, 2012, EMBO Journal) were known to be required for ISC proliferation after midgut damage. This study only reported Ret as a new factor that promotes ISC proliferation after damage. Moreover, a function for the Ret receptor in signalling was already known.

https://www.embopress.org/doi/full/10.15252/embj.201696247

In our study, we have demonstrated a function for Draper and Shark (and now we have new data suggesting a role for phosphatidylserine) as regulators of ISC proliferation after midgut damage. Furthermore, our work ties together how Src-and STAT work together to promote ISC proliferation after midgut damage. We believe that our manuscript presents a more complete understanding of how Src signalling promotes ISC proliferation after midgut damage.

Another reason provided is that "Draper-Shark function in sensing and integrating tissue apoptosis has been reported". However, all of the factors discussed in Cordero et al., 2014 and Perea et al., 2017 have known signalling functions.

Moreover, other recent work also describes the role of factors with a known function in a different context. For example, a study from my department describes the function of TFEB-mediated lysosomal biogenesis in senescence (Curnock et al., 2023, EMBO Journal). A role for TFEB in lysosomal biogenesis was known but here it has been studied in the context of senescence. Furthermore, it's well-established that TFEB is hypo-phosphorylated during stress conditions and regulated by mTORC1 and CDK4, which can be inhibited by p16Ink4a.

https://www.embopress.org/doi/full/10.15252/embj.2022111241

Another example from our field is Zhang et al, 2023, Nature Communications). Here Zhang et al show that Eiger (TNFalpha) is produced by midgut progenitors after damage and stimulates Grindelwald-JNK signalling and EGF production in ECs. Further, they shown that N-glycosylation may influence this signalling. In this example, Eiger is a known ligand for Grindelwald and can activate JNK signalling. It has been shown previously that N-glycosylation can regulate Grindelwald (de Vreede et al, 2018, Dev Cell). It was also known that JNK signalling can stimulate EGF production by ECs that stimulates ISC proliferation (Jiang et al., 2011, Cell Stem Cell).

https://www.nature.com/articles/s41467-024-49786-w

In our study, we also make some other key points:

1. until now Stat was thought to be exclusively regulated by cytokine/JAK signalling. Our work is paradigm shifting for our field. Due to the importance of Src-Stat signalling in mammalian intestinal regeneration, it is likely that our work extends to mammalian intestinal regeneration. Our work could extend to other types of stem cells as well.
2. we show that Draper mediates ISC proliferation without engulfment. This is the first description of Draper function that does not include engulfment. We have provided strong evidence for this.
3. This is the first mechanism described in ISCs to directly sense damage, stimulate ISC proliferation and induce changes in the niche. We now present a damage-sensing mechanism in ISCs that links ligand–receptor interactions to intracellular signaling, ultimately influencing transcription and ISC activity.

As Reviewer 2 said, "I am highly enthusiastic about the value and quality of this study and commend the authors for their work".

It would be great if you could consider these points and advise us whether we should submit an appeal.

Best wishes,

Parthive

Parthive H. Patel, PhD

Group Leader, Wellcome Trust Royal Society Sir Henry Dale Fellow

School of Cellular and Molecular Medicine

Faculty of Health and Life Sciences

University of Bristol

Biomedical Sciences Building, Room G57

University Walk

Bristol, BS8 1TD UK

(44) 117 455 4700

www.gutstresslab.org

X: @gutstresslab

Dear Parthive,

Thank you for your message and following up on our recent decision on your manuscript EMBOJ-2025-121711, reiterating the value of your results for the field and in addition providing a detailed point-by-point response to the critique raised by the referees.

I have now assessed your response in detail and discussed it in the editorial team.

Given your careful discussion of the comments and additional analyses presented on the involvement of phosphatidylserine in ISC damage sensing, and consideration of an alternative Draper ligand, we have concluded that we can invite you to share a manuscript version complemented along the lines sketched in the rebuttal response.

I would then be able to assess such amended manuscript version in detail and share it with the referees for their reassessment. Please note however that given the referees' hesitation regarding the level of clarity and insights on important claims the outcome of such revaluation is entirely open at this time.

Please feel free to contact me if you have any questions or need further input on the referee comments.

We generally allow three months as standard revision time. As a matter of policy, competing manuscripts published during this period will not negatively impact on our assessment of the conceptual advance presented by your study. However, we request that you contact the editor as soon as possible upon publication of any related work, to discuss how to proceed. Should you foresee a problem in meeting this three-month deadline, please let us know in advance and we may be able to grant an extension.

When submitting your revised manuscript, please carefully review the instructions below.

Please feel free to approach me any time should you have additional questions related to this.

Thank you for the opportunity to consider your work for publication.

I look forward to your revision.

Best regards,

Daniel

Daniel Klimmeck, PhD
Senior Editor
The EMBO Journal

Instruction for the preparation of your revised manuscript:

1) a .docx formatted version of the manuscript text (including legends for main figures, EV figures and tables). Please make sure that the changes are highlighted to be clearly visible.

2) individual production quality figure files as .eps, .tif, .jpg (one file per figure).

3) a .docx formatted letter INCLUDING the reviewers' reports and your detailed point-by-point response to their comments. As part of the EMBO Press transparent editorial process, the point-by-point response is part of the Review Process File (RPF), which will be published alongside your paper.

4) a complete author checklist, which you can download from our author guidelines (https://wol-prod-cdn.literatumonline.com/pb-assets/embo-site/Author Checklist%20-%20EMBO%20J-1561436015657.xlsx). Please insert information in the checklist that is also reflected in the manuscript. The completed author checklist will also be part of the RPF.

5) Please note that all corresponding authors are required to supply an ORCID ID for their name upon submission of a revised manuscript.

6) It is mandatory to include a 'Data Availability' section after the Materials and Methods. Before submitting your revision, primary datasets produced in this study need to be deposited in an appropriate public database, and the accession numbers and database listed under 'Data Availability'. Please remember to provide a reviewer password if the datasets are not yet public (see https://www.embopress.org/page/journal/14602075/authorguide#datadeposition).
In case you have no data that requires deposition in a public database, please state so in this section. Note that the Data Availability Section is restricted to new primary data that are part of this study.
*** Note - All links should resolve to a page where the data can be accessed. ***

7) Our journal encourages inclusion of *data citations in the reference list* to directly cite datasets that were re-used and obtained from public databases. Data citations in the article text are distinct from normal bibliographical citations and should directly link to the database records from which the data can be accessed. In the main text, data citations are formatted as follows: "Data ref: Smith et al, 2001" or "Data ref: NCBI Sequence Read Archive PRJNA342805, 2017". In the Reference list, data citations must be labeled with "[DATASET]". A data reference must provide the database name, accession number/identifiers and a resolvable link to the landing page from which the data can be accessed at the end of the reference. Further instructions are available at .

8) At EMBO Press we ask authors to provide source data for the main and EV figures. Our source data coordinator will contact you to discuss which figure panels we would need source data for and will also provide you with helpful tips on how to upload and organize the files.

Numerical data can be provided as individual .xls or .csv files (including a tab describing the data). For 'blots' or microscopy, uncropped images should be submitted (using a zip archive or a single pdf per main figure if multiple images need to be supplied for one panel). Additional information on source data and instruction on how to label the files are available at .

9) We replaced Supplementary Information with Expanded View (EV) Figures and Tables that are collapsible/expandable online (see examples in https://www.embopress.org/doi/10.15252/embj.201695874). A maximum of 5 EV Figures can be typeset. EV Figures should be cited as 'Figure EV1, Figure EV2" etc. in the text and their respective legends should be included in the main text after the legends of regular figures.

- For the figures that you do NOT wish to display as Expanded View figures, they should be bundled together with their legends in a single PDF file called *Appendix*, which should start with a short Table of Content. Appendix figures should be referred to in the main text as: "Appendix Figure S1, Appendix Figure S2" etc. See detailed instructions regarding expanded view here: .

- Additional Tables/Datasets should be labelled and referred to as Table EV1, Dataset EV1, etc. Legends have to be provided in a separate tab in case of .xls files. Alternatively, the legend can be supplied as a separate text file (README) and zipped together with the Table/Dataset file.

10) When assembling figures, please refer to our figure preparation guideline in order to ensure proper formatting and readability in print as well as on screen:
http://bit.ly/EMBOPressFigurePreparationGuideline

Please remember: Digital image enhancement is acceptable practice, as long as it accurately represents the original data and conforms to community standards. If a figure has been subjected to significant electronic manipulation, this must be noted in the figure legend or in the 'Materials and Methods' section. The editors reserve the right to request original versions of figures and the original images that were used to assemble the figure.

11) For data quantification: please specify the name of the statistical test used to generate error bars and P values, the number (n) of independent experiments (specify technical or biological replicates) underlying each data point and the test used to calculate p-values in each figure legend. The figure legends should contain a basic description of n, P and the test applied. Graphs must include a description of the bars and the error bars (s.d., s.e.m.).

Please remember: Digital image enhancement is acceptable practice, as long as it accurately represents the original data and conforms to community standards. If a figure has been subjected to significant electronic manipulation, this must be noted in the figure legend or in the 'Materials and Methods' section. The editors reserve the right to request original versions of figures and the original images that were used to assemble the figure.

Further information is available in our Guide For Authors: https://www.embopress.org/page/journal/14602075/authorguide

We realize that it is difficult to revise to a specific deadline. In the interest of protecting the conceptual advance provided by the work, we recommend a revision within 3 months (29th Oct 2025). Please discuss the revision progress ahead of this time with

the editor if you require more time to complete the revisions.

We would like to thank the reviewers for their helpful suggestions, which have clarified and strengthened our manuscript. We have carefully addressed each comment with clarifications and, where appropriate, additional experiments. Overall, our model and conclusions remain the same after the additional tests outlined below.

We have also added the following new elements to our model:
1. We have identified phosphatidylserine on apoptotic enterocytes as the ligand that Draper recognises after midgut damage.
2. Further, we have determined that Draper-Src-Shark signalling within progenitors requires cytokine-Domeless signalling to promote ISC proliferation and does not regulate cytokine *upd1-3* expression levels in the midgut epithelium after damage. Thus, we propose that Draper-Src-Shark signalling influences cytokine-JAK-STAT signalling in both progenitors and VM by either a) modulating progenitors or b) modulating both progenitors and the regenerative microenvironment. We have elaborated about this in the Discussion. We would thus like to thank the reviewers for encouraging us to re-examine the role for cytokine-Domeless signalling in Src42A signalling within progenitors.
3. Lastly, in addition to midgut damage, we find that enterocyte apoptosis itself can promote cortical accumulation of activated Src in progenitors.

Reviewer 1

Reviewer #1 (Evidence, reproducibility and clarity (Required)):

The manuscript by Legido et al. reports the discovery of a previously unappreciated damage-sensing mechanism in Drosophila intestinal stem cells (ISCs). The authors identify a Draper-Src42A-Shark signaling axis that enables ISCs to recognize damage-induced apoptosis in neighboring epithelial cells and promotes regeneration by activating STAT signaling. The work demonstrates that this pathway functions cell-autonomously within ISCs to promote proliferation and also non-cell-autonomously to activate STAT in the visceral muscle (VM), thereby influencing the regenerative niche. The results presented here are intriguing; however, there is considerable room for improvement in the presentation of the experimental system, the results, and the interpretations of the data. Additional controls are required.

We are delighted that the reviewer found our findings intriguing and thank them for their suggestions to improve the manuscript.

Major issues are listed below.
1. The manuscript is poorly organized. The text jumps from Figure 1 to Figure 3 before discussing Figure 2, making the paper difficult to read.

We thank the reviewer for highlighting this issue. This issue was also noted by Reviewer 2. We agree with both reviewers and have reorganised the manuscript so that the data is presented in the order it is described in the text.

- A schematic diagram of the tissue and cell types should be presented as Figure 1A to orient readers who might not work on this particular tissue.

Please show or schematize and provide references for the Gal4 expression patterns.

We thank the reviewer for this helpful recommendation. We have included a Figure panel 1A diagramming the midgut epithelium, its cell types, and cell type-specific GAL4 expression patterns. We have also included references for each GAL4 expression pattern/system at first mention in the results section.

2. The effect of reducing Drpr, Src, or Shark on the STAT transcriptional reporter damage response seems to be about two-fold. Is that sufficient to explain the effects on ISC proliferation? Do STAT heterozygotes have an effect? Does hyperactivation of STAT have an effect?

We thank the reviewer for these questions. We have updated the % decreases throughout the manuscript by normalising the data to their respective controls. We observe a greater than 50% decrease in STAT activity after *P.e.* infection in progenitors depleted for *draper* (66.6%), *Src42A* (68.7%) and *shark* (74.9%) (Fig. 3K-M). Further, we find that ISC proliferation is also decreased by greater than 50% after *P.e.* infection in midguts with progenitors depleted for *draper* (58.1%), *Src42A* (66.7%) and *shark* (55.6%) (Fig. EV2A, D and G). Thus, we do believe that such a strong reduction in STAT activity could explain the effects we observe on ISC proliferation. This is consistent with previous work showing that depleting STAT in progenitors with *RNAi* blocks ISC proliferation after *P.e.* infection (Jiang *et al*., 2009, *Cell*; Buchon *et al*., 2009, *Genes and Dev*). In contrast, the hyperactivation of STAT by expressing hop or activated JAK Kinase (Hop^{TUM}) with esg^{ts} has been shown to induce ISC proliferation (Buchon *et al*., 2009, *Genes and Dev*; Jiang *et al*., 2009, *Cell*). Moreover, we recently found a proportional decrease in ISC proliferation in midguts of STAT heterozygotes after EC ablation by expressing the pro-apoptotic factor, Reaper, with mex^{ts}. Whilst EC ablation increased ISC proliferation as previously shown in Jiang *et al*., 2009, we found that removing one copy of *Stat92E* in midguts with apoptotic ECs reduced ISC proliferation by 53.5% (Fig 2C). These data suggest that 2-fold reduction in STAT activity reduces ISC proliferation to a similar extent.

3. The authors assume that Draper's function is recognizing PS on apoptotic cells and activating Src as it does in other contexts, but they should test this by addressing the following questions.

Phosphatidylserine (PS) is one of the best-characterised ligands for Draper (Tung *et al*., 2013; Williamson and Vale, 2018, *J. Cell Biology*; Melcarne *et al*., 2019; Hakim-Mishnaevski *et al*., 2019, *Cell Reports*; Ji *et al*., 2023, *PNAS*). Based on this, we indeed proposed that Draper is recognising PS on apoptotic midgut epithelial cells, leading to phosphorylation of its ITAM by Src and activation of Draper.

We were indeed curious to know whether PS is the ligand on apoptotic ECs for Draper found on progenitors. We have now found Draper to be highly expressed on the plasma membrane of midgut progenitors (Fig. 1G-G'). To then test whether PS plays a role in midgut regeneration after *P.e.* infection, we examined ISC proliferation after expressing milk fat globule EGF-8 in ECs with *mex*^{ts}. Milk fat globule EGF-8 is known to bind and mask PS (Tung *et al*, 2013; Hakim-Mishnaevski *et al*., 2019, *Cell Reports*), and if PS was involved, we expected to see a reduction in ISC proliferation. We found that expressing milk fat globule EGF-8 in ECs blocked ISC proliferation in *P.e.*-infected midguts by 54.6% (Fig. 1N). We also tested a role for PS in midgut regeneration after EC ablation as a positive control. To do this, we co-expressed milk-fat globule EGF-8 and the pro-apoptotic factor, Reaper, in ECs with *mex*^{ts}. While expressing Reaper in ECs increased ISC proliferation, we found that expressing milk fat globule EGF-8 in apoptotic ECs blocked ISC proliferation by 55.5% (Fig. 1M). This suggests that the recognition of externalised PS on damaged enterocytes by Draper on ISCs is required for their proliferation.

Moreover, we have tested whether another Draper ligand, Pretaporter (Kuraishi *et al*., 2009, *The EMBO Journal*), is required in ECs to promote ISC proliferation after midgut damage. We found that depletion of *pretaporter* in ECs did not block ISC proliferation after *P.e.* infection, suggesting it is not the ligand for Draper in this context (see Fig. EV1J).

Further, we believe there may be a misunderstanding. As described above, we don't assume that Draper is activating Src42A at the molecular level, if this is what the reviewer suggests, and as far as we know Draper is not thought to molecularly activate Src42A in other contexts. In damaged adult midguts, Src42A is activated by other signals such as Ret and Wg. Upon ligand (phosphatidylserine) binding, Draper microclusters on the plasma membrane. These microclusters exclude bulky transmembrane phosphatases, enabling the ITAM on Draper to remain phosphorylated by Src42A (Ziegenfuss *et al*., 2008, *Nature*; Williamson and Vale, 2018, *J. of Cell Biology*)(Fig. 1O). Phosphorylation of Draper ITAMs by Src42A creates docking sites for Shark, allowing Shark activation by Src42A and driving downstream responses (Williamson and Vale, 2018, *J. of Cell Biology*). We elaborate further on this below (see point b). Our recent data supports this model further. Expression of constitutively active Src42A in progenitors promotes ISC proliferation (Cordero *et al*., 2014, *The EMBO Journal*; Kohlmaier *et al*, 2015, *Oncogene*) and Fig. 2I-J. We found that similar to Shark, Draper is partially required for constitutively active Src42A to promote ISC proliferation (Fig. 2I). Interestingly, we also found that constitutively active Src42A signalling through Draper increased further after infection-induced damage to promote ISC proliferation (Fig. 2I). These data indicate that Src42A can promote ISC proliferation through Draper signalling.

a. Does the p35 expression in EC also block pSrc in the progenitors? This would help connect apoptosis to Drpr-Src-Shark signaling.

We thank the reviewer for this interesting question. We included the pSrc staining in Fig. 1H-I' mainly to localise Src activity within progenitors after *P.e.*-induced midgut

damage. We show that pSrc is localised cortically in progenitors after *P.e.* infection, where the transmembrane receptor, Draper is found (Fig 1G-I'). This supports our model that Src42A activates Draper to promote ISC proliferation after midgut damage (Fig. 2I). Based on previous work (Cordero *et al*., 2014, *The EMBO Journal*; Perea *et al*., 2017, *The EMBO Journal*), Src is activated by Wg and Ret signalling in progenitors after midgut damage. Whilst a mild, general increase in epithelial pSrc has been reported after pathogenic bacterial infection (Cordero *et al*., 2014, *The EMBO Journal*; Houtz *et al.*, 2017), it is not clear whether EC apoptosis causes an increase in pSrc levels in midgut progenitors, and we agree that this would be an interesting observation that would strengthen our model.

Interestingly, EC ablation by expressing *reaper* with *mex*^{ts} did increase pSrc levels within progenitors (Fig. 1K; Fig. EV1F-H), although milder than with *P.e.* infection. We observed an increase of 33.5% in pSrc within progenitors after EC ablation. This indicates that EC apoptosis can increase pSrc levels in midgut progenitors. Since ISCs robustly proliferate after EC ablation (Jiang *et al*., 2009, *Cell;* Fig. 1M), this suggests that even small increases in activated Src may be sufficient to drive ISC proliferation. Furthermore, our data show that EC apoptosis can induce increases in active Src levels to support Draper signalling.

Whilst we have shown that phosphatidylserine exposure by apoptotic ECs is required for ISC proliferation (Fig. 1M-N), this mild increase in pSrc in progenitors after EC ablation further connects apoptosis to Draper-Src-Shark signalling. Moreover, we have shown that both Draper and Src42A are required for ISC proliferation after EC ablation (Fig. 2A-B).

b. How does Drpr RNAi affect pSrc in progenitors?

We thank the reviewer for this suggestion. Based on previous work (Cordero *et al*., 2012, *The EMBO Journal*; Perea *et al*., 2017, *The EMBO Journal*), Src is activated in progenitors by Wg and Ret signalling after midgut damage. Src family kinases are known to phosphorylate ITAMs on immunoreceptors such as Draper and activate Syk (Shark) signalling (Ziegenfuss *et al*., 2008, *Nature*; Williamson and Vale, 2018, *J. of Cell Biology*; Mocsai *et al*., 2010)(Fig. 1O). Williamson and Vale, 2018 showed that ITAM phosphorylation is facilitated by exclusion of a transmembrane phosphatase that allows Src42A to phosphorylate the Draper ITAM. According to this model, Draper does not need to affect Src42A activity to become activated. To make this clearer, we will include a discussion about Draper activation in the Discussion.

c. Is PS recognition required or the domain that recognizes it on Drpr required? Is the ITAM domain of Drpr required?

We thank the reviewer for this suggestion, and we have performed experiments to test if PS recognition is required for ISC proliferation. As discussed above, we have tested this by expressing milk fat globule EGF-8 in ECs with *mex*^{ts} in infected midguts and found that this blocks ISC proliferation (Fig. 1N). Moreover, we have co-expressed milk fat globule EGF-8 and Reaper in ECs and found that masking PS on apoptotic ECs blocks

ISC proliferation (Fig. 1M). Together, these data suggest that PS recognition is required for ISC proliferation.

It is well established that the ITAM domain is required for Draper signalling to recruit Shark (Ziegenfuss *et al*. 2008, *Nature*; Williamson and Vale, 2018, *J. of Cell Biology*), thus we do not feel that this needs to be further retested in midgut progenitors. As Draper and Shark are both required for STAT activation and ISC proliferation after *P.e.*-infection, it is highly likely that both proteins work together like in other contexts.

4. The claim that Draper serves a function other than stimulating engulfment requires stronger experimental support. Currently this interpretation rests solely on a negative result (absence of beta-gal-expressing nuclei within ISCs). It is possible that the apoptotic cells are extruded and are therefore in another focal plane. They may be engulfed and degraded rapidly and therefore not accumulating. The progenitor cells could be nibbling but not engulfing entire cells, so the authors should test that by labeling membranes of the target cells. The authors should demonstrate that they actually can detect engulfment by including a positive control. Live imaging also would be helpful to see where the apoptotic cells are going if they are not engulfed.

We thank the reviewer for these suggestions.

As mentioned in the Discussion, we agree that it is possible that ECs may be extruded from the epithelium and not engulfed. Unfortunately, we currently do not have live imaging set up in our lab to visualise if ECs are extruded into the lumen after apoptosis. Instead, we have now examined where cleaved Dcp-1$^+$ (cDcp1$^+$) apoptotic cells are found after EC ablation by *reaper* expression. We found cDcp-1$^+$ ECs, cDcp-1$^+$ apoptotic bodies and pyknotic nuclei in the midgut epithelium after EC ablation. We scored whether we could observe cDcp-1$^+$ ECs, cDcp-1$^+$ apoptotic bodies and pyknotic nuclei on the basal side of the midgut epithelium (near progenitors) or on the apical side (closer to the lumen) or in the lumen. Upon inducing EC apoptosis, we found cDcp-1$^+$ ECs, cDcp-1$^+$ apoptotic bodies and pyknotic nuclei in all three locations. However, the number of cDcp-1$^+$ ECs, cDcp-1$^+$ apoptotic bodies and pyknotic nuclei all increased progressively from the basal side of the epithelium towards the lumen (Fig. 1D). These data suggest that apoptotic ECs generally are eliminated into the lumen. Nevertheless, a small proportion (10.7%) of apoptotic ECs can be found near progenitors since we consistently found cDcp-1$^+$ ECs, cDcp-1$^+$ apoptotic bodies and pyknotic nuclei basally in the epithelium. Despite this, we were unable to detect cDcp-1 within progenitors (208 in total) after EC ablation (Fig. 5A), indicating that progenitors do not clear apoptotic ECs, at least not at high levels, despite their high Draper expression and activated Src42A. Despite progenitors having high levels of Draper, the mechanism for apoptotic cell clearance may be inactivated for several reasons. This might be due to the large size of cDcp-1$^+$ ECs, cDcp-1$^+$ apoptotic bodies and pyknotic nuclei compared to progenitors. Engulfment would require progenitors to enlarge or stretch to engulf, which we did not observe. Moreover, apoptotic cell clearance by progenitors would be less efficient than eliminating them into the lumen as there are far fewer ISCs (10%) than ECs (70%).

Whilst some studies suggest that ECs undergo apoptosis after being shed into the lumen after infection (Buchon *et al*., 2010, *BMC Biol*; Zhai *et al*., 2018, *Immunity*), others have shown apoptotic ECs (cleaved caspase-3$^+$) within the epithelium after infection (Buchon *et al*., 2009, *Cell Host and Microbe*; Loudhaief *et al*., 2017, *Development*). We found that ECs are positive for cDcp-1 after *P.e.* infection (Fig. 1E-F), suggesting that infection increases the number of apoptotic cells in the epithelium as observed previously, leading to PS externalisation and Draper activation on progenitors. To determine if progenitors engulf dying EC corpses after infection, we expressed GFP in ECs with *mex^{ts}* and ascertained if GFP$^+$ EC corpses can be found within progenitors. Indeed, we did not detect GFP$^+$ EC corpses within progenitors after infection (Fig. 5B). This is consistent with our data suggesting that apoptotic ECs are shed into the lumen after EC ablation and *P.e.* infection (Fig. 1D).

We agree that it is possible that EC corpses may be engulfed and degraded rapidly, and thus our assay may not be able to detect engulfed and degraded ECs. Nevertheless, if progenitors are phagocytic, we should expect to observe some corpse engulfment since this is a dynamic process with cells dying at varying times after both EC ablation and infection. For example, apoptotic cells can be found within the phagosomes of embryonic glia and plasmatocytes near the developing CNS of *Drosophila* embryos (Kurant *et al*., 2008, *Cell*, see Fig. 4). Nevertheless, we blocked lysosomal degradation of phagosome cargo by inhibiting the fusion of mature phagosomes with the lysosome by expressing dominant negative Rab7 (T22N) in progenitors of infected midguts (Melcarne *et al*., 2019, Han *et al*., 2014, *Neuron*). We then examined whether engulfed EC corpses (ECs marked with ß-gal) could be found within these progenitors. However, we did not detect ß-gal$^+$ EC corpses after infection in progenitors expressing dominant negative Rab7 (T22N) (Fig. EV5C-H), suggesting that internalised apoptotic EC corpses were unlikely to rapidly undergo lysosomal degradation if engulfed.

Further, we agree that it is possible the progenitors may be nibbling on dying ECs. To test this, we tried to express several membrane-localised fluorescent proteins (myr-RFP (see images below, A-B), and myr-Tomato (C-D), CD8-GFP (E-F) and PLCδ-PH-GFP (G-H)) in ECs with *mex^{ts}*, but found that all of these membrane-targeted fluorescent proteins inserted into lateral (more apical) membranes of ECs and not into the lateral (more basal) EC membrane (basal junction) near the progenitors. Since E-Cadherin is found in the lateral (more basal) membrane (within adherens junctions) of ECs, we also expressed a fluorescently tagged E-Cadherin (E-Cad-EosFP) (I-J) within ECs with *mex^{ts}*. Unfortunately, E-Cad-EosFP did not incorporate into the lateral (more basal) membrane of ECs. We asked Dr Jia Chen and Prof. Daniel St Johnston (University of Cambridge)- experts on EC polarity and the basal junction- for advice on how to label the EC lateral (more basal) membrane, but they could only suggest E-Cadherin to us. We also expressed LifeAct-GFP (K-L) in ECs to label the actin cortex underlying the plasma membrane; however, we found LifeAct-GFP mostly on the apical side of ECs.

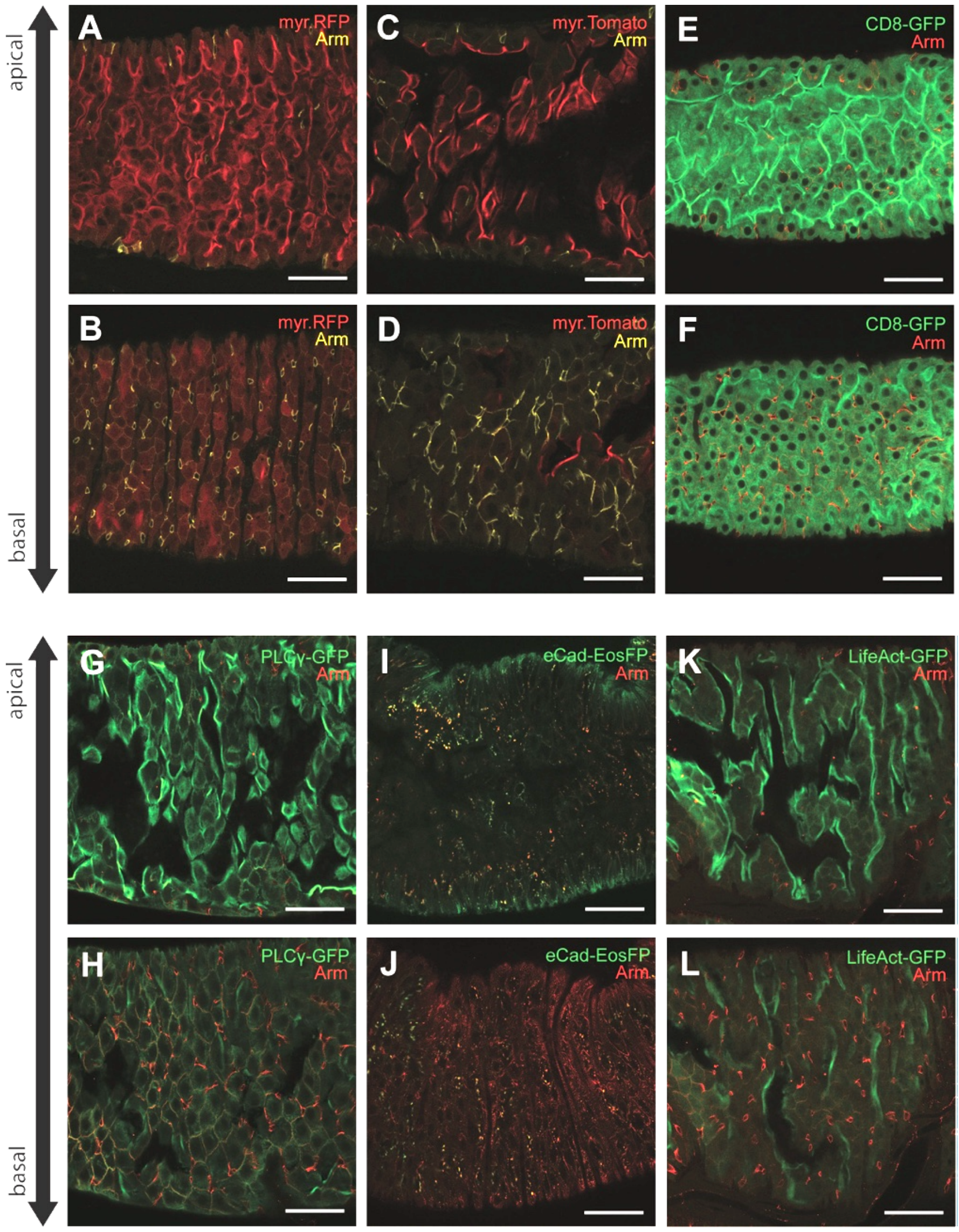

We have also recently expressed a dominant-negative Rac (Rac^{N17}) in progenitors of infected midguts, which blocks engulfment. This is due to Rac being required for actin polymerisation for most types of engulfment (Mao and Finnemann, 2015). We confirmed this through discussions with Prof. Anne Ridley (University of Bristol)–an expert on Rho small GTPases and actin polymerisation–and with Prof. Estee Kurant (University of Haifa)–an expert on engulfment and Draper signalling. In *Drosophila* egg chambers, Draper is required for Rac^{V12}-driven engulfment of nurse cells by border cells (Mishra *et al*., 2023, *PNAS*). Further, Rac^{N17} expression blocked STAT activation in glia after axonal injury of olfactory receptor neurons (Doherty *et al*., 2014, *PLOS Biology*). We found that Rac^{N17} expression in progenitors of infected midguts—blocking Rac signalling, and thus, Rac-mediated engulfment—did not block STAT activity (Fig. 5C). These data suggest that neither Rac signalling nor Rac-mediated engulfment are required in progenitors for STAT activation after midgut damage, whereas Draper is, adding further our model.

Although we were unable to detect apoptotic EC corpses in progenitors, even after blocking lysosomal degradation, and we could not examine progenitors nibbling on dying ECs, we will state these possibilities in the Discussion. Even if progenitors did engulf EC corpses or nibble on apoptotic ECs, Rac-mediated engulfment does not seem to be required for STAT activation, and thus, ISC proliferation (Fig. 5C).

A role for Draper in phagocytosis, which includes engulfment and degradation, has been studied in several contexts, including embryonic central nervous system (Kurant *et al*., 2008), larval and pupal peripheral nervous system (Han *et al*., 2014), the adult olfactory system (MacDonald *et al*., 2006), the testis (Zohar-Fux *et al*., 2022) and the ovary (Etchegaray *et al*., 2012). All these structures do not have the ability to easily remove cell debris into the external environment like the adult midgut. This might be the reason why clearance through phagocytosis has been reported by plasmatocytes and glia in the embryo (Kurant *et al*., 2008), larval and pupal epidermal cells (Han *et al*., 2014), glia in the adult olfactory system (MacDonald *et al*., 2006), cyst cells in the testis (Zohar-Fux *et al*., 2022) and follicle cells in the ovary (Etchegaray *et al*., 2012). Our data indicate that apoptotic ECs in the midgut are pushed out into the lumen and likely excreted by adult flies. This would not require apoptotic cell clearance by midgut progenitors and may explain why we do not detect cDcp-1^{+} ECs or pyknotic nuclei within progenitors. However, progenitors with their high Draper levels should be able to sense apoptotic ECs after phosphatidylserine exposure. Moreover, while Draper has been reported to be involved in clearance in the contexts above, it is not always required for engulfment. For example, Draper is not required in embryonic glia or plasmatocytes for their engulfment of apoptotic cells in the developing CNS (Kurant *et al*., 2008, *Cell*, see Fig. 4). Instead, Draper is required for the degradation of engulfed apoptotic corpses.

Regarding our ability to detect engulfment, key considerations include fixation, the z-step taken during confocal microscopy and the frequency of the observed event. We were not able to obtain this information from other studies, so it was difficult to reproduce published engulfment experiments. We have reported this information in our manuscript to ensure reproducibility. We have used two fixation conditions to look for engulfed corpses (8% formaldehyde and 8% formaldehyde, followed by ethanol/methanol fixation, see Methods). With the latter fixation method, we have been able to preserve delicate cDcp1^{+} apoptotic cells, bodies and debris. Thus, we believe that we should have been able to preserve engulfed corpses. We have acquired confocal stacks with a z-step of 0.5μm. This is similar to confocal z-steps (6 sections, 3μm) used in Kurant *et al*., 2008, *Cell*, which showed apoptotic cells within glia and plasmatocytes in the developing *Drosophila* embryo. Similarly to other studies that reported corpse engulfment, we have labelled dying cells with apoptotic markers (activated caspase) (Kurant *et al*., 2008, *Cell*) or fluorescent proteins (Etchegaray *et al*., 2012, *Development*). We had discussed both of these engulfment assays with Prof. Estee Kurant (University of Haifa)- an expert on phagocytosis in *Drosophila*. Nevertheless, we did not detect engulfed corpses within progenitors using three different assays. Draper is not required for engulfment in all contexts (Kurant *et al*., 2008, *Cell*), suggesting that Draper could be used differently in the adult midgut. Moreover, our data suggest that STAT activation by Draper-Src-Shark signalling in

progenitors after midgut damage does not require Rac signalling nor Rac-mediated engulfment.

5. No data are provided to support the claim that Draper-Src-Shark-STAT signaling operates independently of the cytokines (Upd1-3) and their receptor Domeless. It would be relatively straightforward to look at Upd expression or test the effects of expressing dominant-negative Domeless in specific cell subsets (ISCs and VMs) using available Gal4 drivers.

We thank the reviewer for this suggestion. We agree that it is possible that STAT activity could increase in progenitors and VM due to the upregulation of Upds1-3 after midgut damage.

Previously, it was reported that cytokine-Domeless signalling is not required for ISC proliferation induced by constitutively active Src42A in progenitors (Cordero *et al*., 2014, *The EMBO Journal*; Kohlmaier *et al*., 2015). To determine whether Draper-Src-Shark signalling regulates STAT activity independently of cytokines, we performed two experiments. We re-tested whether constitutively active Src42A requires the cytokine receptor, Domeless, to promote ISC proliferation, and we determined whether cytokine *upd1-3* expression was regulated by Draper signalling in progenitors after *P.e.* infection.

We found that expression of constitutively active Src42A in progenitors stimulates ISC proliferation, partially through Draper and Shark (Fig. 2I-J) and is sufficient to activate STAT in progenitors and the visceral muscle (Fig. 4J; Fig. EV4G-K). We retested if constitutively active Src42A required the Domeless receptor for ISC proliferation. We found that depletion of *domeless* in progenitors inhibited ISC proliferation after *P.e.* infection (Fig. EV4L), as previously described (Jiang *et al*., 2009; Buchon *et al*., 2009). However, we also surprisingly found that depletion of *domeless* inhibited ISC proliferation caused by constitutively active Src42A signalling (Fig. 4K). This suggests that cytokine-Domeless signaling is required for STAT activation in progenitors due to Draper-Src-Shark signalling. Unlike the other studies, we showed our tool to inhibit Domeless signalling ($dome^{RNAi}$) suppressed ISC proliferation after *P.e.* infection.

To test whether cytokine (Upd1-3) expression in the midgut epithelium was regulated by Draper signalling in progenitors, we performed qPCR for *upd1-3* expression in infected midguts with progenitors depleted for *draper*. We found that Draper signalling in progenitors was not required for *upd1-3* expression by the midgut epithelium (Fig. 4L).

Together, this suggests that Draper signalling in progenitors regulates STAT activation in progenitors, and likely too in the VM, via cytokine-Domeless signalling.

Unfortunately, currently it is technically not possible for us to overexpress constitutively active Src42A in progenitors and simultaneously deplete *domeless* in the VM. JAK-STAT activity in VM stimulates EGF (Vein) production, which stimulates ISC proliferation (Jiang *et al*., 2011, *Cell Stem Cell*; Zhou *et al*., 2013, *Dev Biol*). It has been shown that depleting *domeless* in the VM reduces ISC proliferation after infection (Zhou *et al*., 2013). We have included this in the Discussion.

6. No data are provided to support the claim that phosphatidylserine on the membrane of apoptotic enterocytes activates Draper/Src/Shark signaling.

We had proposed that phosphatidylserine (PS) activates Draper-Src-Shark signalling. As described above, we have now tested this by expressing milk fat globule-EGF-8 in progenitors with *esg*^{ts} in infected midguts and found that this indeed blocks ISC proliferation (Fig. 1M-N).

7. No mechanism is tested or proposed for how Src activates STAT.

We found Src42A to be required and sufficient for STAT activation in progenitors (Fig. 3G-H", L; Fig. EV3C-H"; EV4G-I). We have proposed that Src can activate STAT via Draper and Shark based on their similar effects on STAT activity in progenitors and the VM after *P.e.* infection. Several studies have shown that immunoreceptors such as Draper and the tyrosine kinase Syk (or Shark) are activated by Src family kinases (Ziegenfuss *et al*., 2008, *Nature*; Williamson and Vale, 2018, *J. Cell Biology*; Mocsai *et al*., 2010), and that this signalling module can activate STAT (Doherty *et al*., 2014, *PLOS Biology*). Moreover, STAT activity promotes ISC proliferation after midgut damage (Jiang *et al*., 2009, *Cell*; Buchon *et al*., 2009, *Genes Dev*), and we have also shown that both Draper and Shark are required for ISC proliferation due to constitutively active Src42A expression in progenitors (Fig. 2I-J). We have also now shown that constitutively active Src42A requires the cytokine receptor, Domeless, to induce ISC proliferation (Fig. 4K; Fig. EV4L). Moreover, we have shown that Draper signalling is not required in progenitors for cytokine *upd1-3* expression in the midgut epithelium after *P.e.* infection (Fig. 4L). Together, our work suggests that Src activates STAT through Draper-Shark signalling in progenitors and cytokine signalling in both progenitors and the VM.

8. The non-autonomous effect is surprising but unexplored. A potential mechanism that would explain both how Draper-Src-Shark activates STAT in the ISC and in the muscle would be if Upds are secreted, which is a well-established effect of injury.

We thank the reviewer for this suggestion. We included effects on STAT activity in the VM because it is intriguing and for completeness, as STAT activity is also induced in the VM after *P.e.* infection. Nevertheless, we have determined by qPCR if *upd1-3* expression could explain these effects. As described above (point 5), we found that inhibiting Draper signalling in progenitors did not block cytokine *upd1-3* expression in the midgut epithelium after damage (Fig. 4L). Thus, the reduction in STAT activity in progenitors and in the VM by inhibiting Draper-Src-Shark signalling in progenitors is not due to *upd1-3* expression in the midgut epithelium. We propose that Draper-Src-Shark signalling influences cytokine-JAK-STAT signalling in both progenitors and VM by either a) modulating progenitors or b) modulating both progenitors and the regenerative microenvironment. We have elaborated about this in the Discussion. The precise mechanism by which Domeless signalling contributes to Draper-mediated ISC proliferation following midgut damage is still unclear. However, our key findings- that ISCs directly sense midgut damage, the mechanism by which they do so, and the resulting downstream responses are clearly established by our current data.

Minor issues
1. ISC is not defined when first mentioned

We apologise for this oversight, and we have added this to our revised summary.

2. Figure 1D-G, show representative images along with quantifications.
Why are the scales different on the different STAT graphs? makes it hard to compare between the figures.
Why are axes different numbers in 1F ?

The height of the y-axis in 1F (now 2F) is different to improve the visibility of the data. For the STAT graphs: 1) the scales on the y-axis are every 5 progenitors for all STAT graphs, but the scales vary for the x-axis in Figure 2 (every 2000 in Fig. 2 vs 5000 in Figs. 3 and 4) because the STAT activity was induced less in this experiment. These data can now be found in Appendix Fig. S1.

3. Please move Supplemental figures 1C, 2C 3C into the main figures

We have moved Supplemental Figs. 1C, 2C and 3C to the main figures (Fig. 3) in our revised manuscript.

Reviewer #1 (Significance (Required)):

This work in principle provides a conceptual advance in stem cell biology and regeneration by proposing a new function for the molecular machinery best known for innate immune responses: specifically that the Draper - Src - Shark pathway that is best characterized in phagocytosis plays a role in stimulating STAT upon tissue damage. This work will be of interest to basic scientists studying stem cell responses to tissue injury. It has relevance for tissue repair, regeneration, and aging. The limitations of the study are described above and include the confusing presentation as well as missing controls and insufficient data to support some of the conclusions.

We thank the reviewer for noting that "our work will be of interest to basic scientists studying stem cell responses to tissue injury", and its "relevance for tissue repair, regeneration, and aging".

Reviewer 2

Reviewer #2 (Evidence, reproducibility and clarity (Required)):

Damage recognition by intestinal stem cells via Draper-Src-Shark-STAT signalling promotes adult Drosophila midgut regeneration.

In this manuscript, Legido et al present evidence that the Src42A kinase in Drosophila is essential for epithelial regeneration in damaged fly midguts. The work is performed to a very high standard; images are excellent throughout; statistics are appropriate, and

sample sizes are high; the authors clarify size effects throughout their work; all data are easily followed and appropriately interpreted.

As a result, I am highly enthusiastic about the value and quality of this study and commend the authors for their work. Most of my concerns are relatively minor and are detailed below.

We thank the reviewer for this positive evaluation of our work. We are delighted to hear that the reviewer is highly enthusiastic about the value and quality of our study.

MAJOR ISSUES

1. Src as a bacteria sensor. I struggle with the rationale behind Figure 1E. The authors claim these data exclude possible roles for Src as a molecule that "detects pathogenic bacteria". I may be incorrect, but it appears the experiment is intended to test if Src is a classical pattern recognition receptor (PRR) that senses microbial patterns. It's unclear what would lead to this hypothesis, as Src does not have the molecular structure or prior literature we would expect to see in PRRs. Nonetheless, I do not agree that the results exclude roles for Src in sensing bacterial patterns (regardless of how unlikely the possibility is). The experiment in Figure 1E was performed with conventional flies with a conventional microbiome, so we cannot formally exclude the possibility that Src "senses" dysbiotic commensal bacterial patterns in an SDS-treated gut. To exclude this possibility, it would be important to repeat Figure 1E in axenic flies. For me, the data in Figure E are valuable, but primarily because they demonstrate that Src is also capable of initiating repair in response to chemical stress, suggesting that the phenotype is not exclusive to a unique feature of Pe infection. If the authors wish to exclude roles for Src as a PRR, they should be encouraged to stress axenic guts. As it is highly unlikely that Src is a PRR, I feel the authors would be best advised to consider reorganizing the text to clarify that their work extends prior observations by showing that Src also promotes renewal in response to a chemical stress.

We thank the reviewer for highlighting this issue. It was indeed not our aim to determine whether Src acts as a pattern recognition receptor (PRR). We agree with the reviewer that this would require exposing axenic flies to detergent (SDS) stress to rule out detection of dysbiotic commensal bacteria. We have edited the text to point out that Src is required for ISC-mediated regeneration in response to damage caused by both pathogenic bacteria and chemicals (detergents).

2. Possible involvement of the UPD/Domeless pathway. At times, it appears that the authors argue their data exclude roles for UPD signaling in STAT activation by Src (e.g. penultimate sentence of Introduction, relevant text in Results section, paragraph of Discussion). The authors are correct that prior studies argue against Domeless involvement, but none of the experiments in this manuscript test that hypothesis further. As a result, I encourage the authors to be more precise in their discussion of Upd signaling.

We thank the reviewer for pointing this out, and we agree completely. Our data did not exclude a role for Upd1-3 in STAT activation by Src, thus we have now modified the text to reflect our current data.

To our surprise, we found that Domeless signalling is required for constitutive active Src to promote ISC proliferation (see Reviewer 1, point 5). This is contrary to the findings from Cordero *et al*., 2014, *The EMBO Journal* and Kohlmaier *et al*., 2015. Furthermore, we found that Draper signalling in progenitors does not regulate *upd1-3* expression in the midgut epithelium. Thus, our data suggests that Draper-Src-Shark signalling promotes STAT activation through cytokine-Domeless signalling in progenitors and in the VM.

MINOR ISSUES

1. Regenerative Niche: I struggle with the concept of a regenerative niche in the fly gut. As the authors point out essentially all intestinal cells (enteroblasts, enterocytes, enteroendocrine, muscle, trachea) influence stem cell behaviour in the fly gut. As a result, it's difficult to agree that there is a subset of specialist "niche" cells that specifically regulate stem cell proliferation in the fly midgut. I find the term "microenvironment" (used in the Introduction) more appropriate for flies.

We thank the reviewer for their suggestion to use “microenvironment” over “niche”. We agree that there is not a subset of specialist “niche” cells that specifically regulate ISC proliferation during midgut regeneration. Therefore, we have now used the term “regenerative microenvironment”.

2. This sentence from the Introduction requires modification: "Mechanistically, Draper-Src-Shark signalling stimulates ISC proliferation by increasing STAT activity in ISCs, which has previously been thought to be exclusively activated by cytokine (Upd1-3)/JAK signalling." As the authors point out at an earlier stage, prior work had already established that Upd1-3 are not needed for Src-dependent STAT activation: "Domeless and its ligands, Upd1-3, are not required for Src42A to stimulate ISC proliferation through STAT activation".

We thank the reviewer for pointing this out. We agree with the reviewer and have now modified our text and model figure (Fig. 5) to reflect our new data.

As described above (see Reviewer 1, point 5), we surprisingly found that Domeless signalling is required for constitutively active Src42A to promote ISC proliferation. Furthermore, we found that Draper signalling in progenitors is not required for *upd1-3* expression in the midgut epithelium. Together, our data suggest that Draper-Src-Shark signalling activates STAT through cytokine-Domeless signalling in progenitors and in the VM.

3. Methods: More detail is needed for the infection protocol. The authors should detail their handling of Pe (growth medium; all steps taken to centrifuge, resuspend, etc. Pe prior to infection; definition of 20X Pe ideally as CFU/ml, or OD). Otherwise, it will be

very difficult for others to follow up on this study. Also, please clarify if females used in these experiments were mated or virgin.

We thank the reviewer for helpful recommendation. We have included more detail about our infection protocol to improve the reproducibility of our experiments. In this study, we used mostly virgin females, but we have performed a few experiments with mated females. We have indicated which experiments used mated females in our methods section. We find that ISC regenerative response is similar between mated and virgin females; the mean number of mitoses (proliferating ISCs) per midgut is similar between *P.e.*-infected mated and virgin females.

4. Quantifications: When introducing data in Figure 3, please consider including one or two sentences explaining what we are looking at in panels L-O. For many within the field, it is likely intuitive, but I wonder if those less familiar with Drosophila will follow along.

We thank the reviewer for highlighting this issue. We apologise for not making this clear in our manuscript. In Figure 3, Panels L-O are histograms of STAT-GFP levels in progenitors of uninfected control midguts (L), *P.e.*-infected control midguts (M), control midguts expressing *shark*^{RNAi} in progenitors (N) and *P.e.*-infected midguts expressing *shark*^{RNAi} in progenitors (O). This data has now been moved to Appendix Fig. S1. We have now better described what these data are in the Results section.

5. Organization of data. For now, the arrangement of the figures is slightly confusing, and seldom aligns with the text. I find this diminishes the quality of the manuscript. I recommend the authors consider rearranging all figures to directly match the text. For example, STAT:GFP images and quantifications are discussed in the section "Draper-Src-Shark signalling promotes ISC proliferation through STAT activation". It would make most sense for these data to all exist in a single matching figure. These issues apply throughout (Figure 3 and 4 are discussed prior to Figure 2, text for Figure 5 follows text for figure 6, etc.). This does not require any additional experiments but would make the text much more accessible to readers.

We thank the reviewer for this helpful suggestion. We completely agree, and we have improved our organisation of the data by rearranging the figures, so they align with the text.

6. Figure 7: I appreciate the inclusion of a speculative model in Figure 7. However, I feel the accompanying text is more appropriate for the discussion than the result, and there are cases where the wording could be moderate (e.g. "This allows cortically-localised activated Src to phosphorylate the ITAMs on Draper" is not supported by data demonstrating phosphorylation of ITAMs on Draper by Src).

We thank the reviewer for pointing this out. We agree that this text describes our speculation of how Draper-Src-Shark signalling functions in progenitors after midgut damage and belongs in the Discussion. Our speculation is based on a number of studies describing immunoreceptor-Src family kinase-Syk/Shark signalling in both

Drosophila and humans (Ziegenfuss *et al*., 2008, *Nature*; Williamson and Vale, 2018, *J. of Cell Biology*; Mocsai *et al*., 2010). We have now moved this speculation to the Discussion.

7. Returning to the theme of Src as a potential PRR, the following sentence from the discussion confuses me: "This suggests that Src42A in ISCs senses tissue damage rather than pathogenic bacteria themselves, possibly through the recognition of pathogen-associated molecular patterns (PAMPs)." Here, the authors appear to suggest that Src detects PAMPs. Is that correct? The hypothesis is not supported by the accompanying data. Presumably they are arguing that Src does not detect PAMP. If so, the text could be clarified. Additionally, I would encourage the authors to consider microbe-associated molecular pattern (MAMP) over PAMP.

We thank the reviewer for these suggestions. We apologise for the confusion. Our aim was to highlight that Src senses tissue damage. Our data shows that Src42A is required in progenitors for the ISC response to tissue damage caused by both pathogenic bacteria and detergents. We have modified the text to reflect this better.

Reviewer #2 (Significance (Required)):

The data are consistent with earlier reports by Cordero et al (EMBO 2014), and Kohlmaier et al. (Oncogene 2015). However, this study goes further by presenting two important discoveries. First the authors present convincing genetic evidence that Src-dependent regeneration involves participation of Draper and Shark. Secondly, the authors provide data that indicate Draper initiates Src signaling in a phagocytosis-independent manner. As a result, the work constitutes an important extension of previous work and adds to our understanding of epithelial regeneration in fly guts. The exact mechanism by which dying enterocytes activate Draper in ISCs requires clarification, and possible involvement of visceral muscle hinted at in figure six also requires clarification. However, the current study will doubtlessly be of interest to researchers using flies to characterize epithelial cell dynamics during periods of stress and should be of appeal to a larger community interested in epithelial regeneration. I

We thank the reviewer for this positive evaluation and for highlighting our two important discoveries: 1) Src-dependent regeneration requires Draper and Shark, and 2) Draper-Src-Shark signalling can promote regeneration in a phagocytosis-independent manner. We have now provided additional data to support these conclusions.

As discussed above, we have clarified how dying enterocytes activate Draper in ISCs by examining one of the ligands that activate Draper, phosphatidylserine (PS). We found that masking externalised phosphatidylserine on dying ECs by expressing milk fat globule EGF-8 reduced ISC proliferation in infected midguts and in midguts with ECs expressing the pro-apoptotic factor, Reaper (see Fig. 1M-N).

As for the possible involvement of the visceral muscle, we currently believe that increased STAT activity in the VM will stimulate EGF production (Vein) that will further boost ISC proliferation as proposed by Jiang *et al*., 2011, *Cell Stem Cell*; Zhou *et al*.,

2013, *Dev Biol*. We will include some text in the Discussion describing this work and placing it into the context of our work.

Reviewer 3

Reviewer #3 (Evidence, reproducibility and clarity (Required)):

In this manuscript, Legido et al. investigate the role of Draper-Src-Shark-STAT signaling in regulating intestinal stem cell (ISC) proliferation upon bacterial infection using the Drosophila intestine model. They demonstrate that Draper-Src-Shark signaling acts in ISCs to recognize midgut damage and is required for ISC proliferation following bacterial infection. In addition, they show that Draper-Src-Shark signaling regulates STAT activation both in progenitor cells and the visceral muscle.

Major concerns:
1. While the authors use Mex-Gal4 to drive gene expression in Ecs and examine pSrc, it would be better to co-express UAS-GFP to clearly mark Ecs.

We thank the reviewer for this suggestion. We agree that this would be ideal. The differentiated cells in the epithelium include ECs and EEs. The ECs are much larger than EEs and can be identified by their large polyploid nucleus. We have identified ECs here using these two criteria. We have now marked a few EC nuclei with an asterisk and used green arrows to indicate pSrc increases at EC membranes (Fig. EV1A-B'). As Draper is a transmembrane immunoreceptor located in the plasma membrane, we wanted to show where activated Src (pSrc) is localised after *P.e.* infection. We found that pSrc accumulates cortically in progenitors after EC ablation and *P.e.* infection (Fig. 1H-J), where it could phosphorylate the ITAM within Draper. Moreover, we have now found that Draper is highly expressed on the plasma membranes of *esg*$^{+}$ progenitors (Fig.1G-G').

2. It is difficult to see the upregulation of pSrc in ECs (Supplementary Fig. 1D-G'). To confirm the specificity of the pSrc antibody, the authors should knock down Src42 using esg-Gal4 during P.e. infection and examine the results.

We thank the reviewer for this suggestion. We have included arrows where there are changes in pSrc in progenitors (yellow arrows) and ECs (green arrows) after infection (Fig. EV1A-D'). We have further included a quantitation of pSrc levels from the whole epithelium, which is about 70% ECs, showing that pSrc levels increase with infection but decrease in midguts with ECs expressing *Src42A*^{RNAi} by 97.1% (Fig. EV1E). While these measurements are not cell type-specific, these data suggest that Src42A increases in the midgut epithelium after infection and that the antibody is specific for Src42A phosphorylation. Please note that several studies have used this pSrc antibody in the midgut but never tested its specificity (Cordero *et al.*, 2014, *The EMBO Journal*; Perea *et al.*, 2017, *The EMBO Journal*; Houtz *et al.*, 2017, *PLOS Genetics*).

Furthermore, we have used another anti-pSrc antibody (used in Torres *et al.*, 2023, *J. Cell Biology*), which showed a similar cortical increase in pSrc levels in progenitors

after *P.e.* infection. Images below show *P.e.*-infected midguts (B-B') and control uninfected midguts (A-A'). Yellow arrows indicate progenitors.

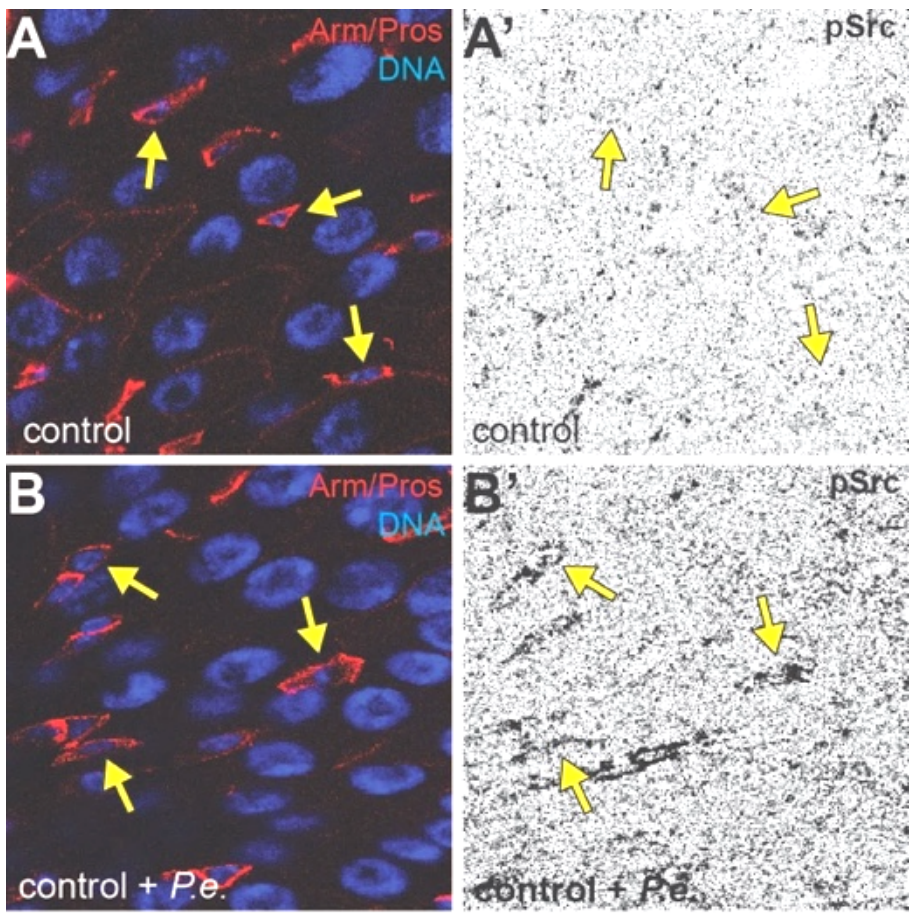

3. Although the authors show that Shark is required for Src42A-mediated ISC proliferation, they should also test whether Src42A is required for Draper-induced ISC proliferation to complete the signaling pathway analysis.

We thank the reviewer for this suggestion. Based on the literature, we expect Src family kinases to phosphorylate the ITAM within immunoreceptors such as Draper to allow Shark/Syk signalling (Ziegenfuss *et al*., 2008; Williamson and Vale, 2018, *J. of Cell Biology*; Mocsai *et al*., 2010). Thus, it is highly likely that Src42A is required for Draper signalling.

Based on our study and prior work, we can only activate Draper by expressing constitutively active Src42A, by inducing apoptosis in ECs or by *P.e.* infection. We have already shown that, like Draper, Src42A is required in progenitors for ISC proliferation and STAT activation after *P.e.* infection. Thus, we examined ISC proliferation in Src42A heterozygous midguts expressing *reaper* in ECs, which induces EC apoptosis, thereby activating Draper. Whilst we found an increase in ISC proliferation after EC ablation, we found a 44.7% decrease in ISC proliferation in midguts of Src42A heterozygotes expressing *reaper* in ECs (Fig. 2B).

4. The authors conclude that "these data indicate that Draper-Src-Shark signaling promotes ISC proliferation through STAT activation." However, the current data only show that STAT activation upon P.e. infection depends on Draper-Src-Shark signaling. To strengthen this conclusion, the authors should activate Draper-Src-Shark signaling and determine whether STAT knockdown can rescue the ISC proliferation phenotype.

We thank the reviewer for this suggestion. STAT has been shown to be essential in progenitors for ISC proliferation after midgut damage (Jiang *et al*., 2009, *Cell*; Buchon *et al*., 2009, *Genes Dev*). Furthermore, both Cordero *et al*., 2014 and Kohlmaier *et al*., 2015 showed that STAT is required for ISC proliferation caused by expression of constitutively active Src42A in progenitors. Moreover, the 10X STAT-dGFP reporter for STAT activity requires Stat92E (Bach *et al*., 2007), and we have shown that Draper-Src-Shark signalling promotes STAT activation (10X STAT-dGFP) after *P.e.* infection (Fig. 3).

As mentioned above, we can only activate Draper-Src-Shark signalling either by expressing constitutively active Src42A, by *P.e.* infection or by expressing pro-apoptotic factors in ECs. Thus, we examined ISC proliferation in *Stat92E* heterozygous midguts expressing *reaper* in ECs. Whilst EC ablation increased ISC proliferation, in *Stat92E* heterozygotes, we found a 53.5% decrease in ISC proliferation in midguts expressing *reaper* in ECs (Fig. 2C). Since Draper is highly expressed on progenitors, our data suggest that EC apoptosis, which activates Draper, promotes ISC proliferation through STAT.

5. The authors state that "p35 expression in ECs partially blocked ISC proliferation by 40.1% in P.e.-infected midguts compared to controls (Fig. 5A), suggesting that some infected ECs undergo apoptosis." However, they should directly examine apoptosis markers in response to P.e. infection to support this claim.

We thank the reviewer for this suggestion. Apoptosis in the midgut epithelium after infection has been previously described (Buchon *et al*., 2009, *Cell Host and Microbe*; Buchon *et al*., 2010, *BMC Biol*; Loudhaief *et al*., 2017, *Development*; Zhai *et al*., 2018, *Immunity*). These studies concluded that apoptosis can be either observed in the epithelium or in ECs shed into the lumen or both. Further, it has been shown that expressing p35 or DIAP1 in ECs can block ISC proliferation after *P.e.* infection (our study and Phillips *et al*. DOI: https://doi.org/10.1101/2025.04.16.649133) and DNA-damage (bleomycin) (Amcheslavsky *et al*., 2009), indicating that EC apoptosis is required for ISC proliferation after infection. We have now directly examined apoptosis in the midgut epithelium after *P.e.* infection using an antibody against cleaved Dcp-1 ($cDcp1^+$). We found that *P.e.* infection increased the number of cDcp-1^+ ECs in the midgut epithelium (See Fig. 1E-F and Reviewer 1 point 4). As a positive control, we induced apoptosis in ECs by expressing *reaper* with mex^{ts}. We found a strong increase in cDcp-1^+ ECs in midguts expressing *reaper* with mex^{ts} (see Fig. 1B-D). Together, these data suggest that ECs undergo apoptosis after *P.e.* infection.

In support of our data suggesting that EC apoptosis is required for ISC proliferation, we found that overexpression of the anti-apoptotic factor, DIAP1, in ECs blocked ISC proliferation in *P.e.*-infected midguts by 57.8% (Fig 1L). Taken together with our data suggesting a role for externalised phosphatidylserine on ECs for ISC proliferation after infection, these data strongly suggest a role for apoptosis recognition in ISC proliferation after midgut damage. Our data showing that inhibiting apoptosis with p35 can suppress ISC proliferation after *P.e.* infection can now be found in Fig. EV1I.

6. To further test the relationship between apoptosis and Draper-Src-Shark signaling, the authors should induce apoptosis in ECs while blocking Draper-Src-Shark signaling and assess whether ISC proliferation is rescued.

We thank the reviewer for this suggestion. Currently it is technically not possible for us to simultaneously manipulate both the ECs and progenitors. Thus, we examined ISC proliferation in midguts of *draper* heterozygotes expressing *reaper* in ECs. Whilst EC ablation increased ISC proliferation, removing one copy of *draper* suppressed ISC proliferation by 35.2% (Fig. 2A). These data suggest that ISC proliferation triggered by

EC apoptosis requires Draper. Indeed, Draper is likely required in progenitors as it is highly expressed on these, and depleting *draper* in ISCs inhibits their proliferation after *P.e.*-induced damage.

7. The authors demonstrate that Draper-Src-Shark signaling in progenitor cells regulates STAT activity non-cell autonomously in the visceral muscle. To strengthen this conclusion, they should activate Draper-Src-Shark signaling specifically in progenitor cells and examine STAT-GFP levels in muscle cells.

We thank the reviewer for this suggestion. We expressed constitutively active Src42A in progenitors and examined its effects on STAT-GFP in both progenitors and the visceral muscle. We found that Draper is partially required for constitutively active Src42A signalling to promote ISC proliferation (Fig. 2I), which requires STAT. Moreover, we found that expressing constitutively active Src42A in progenitors strongly activated STAT-GFP in both progenitors and in the visceral muscle (see Fig. 4J; Fig. EV4G-K). These data suggest that Draper-Src-Shark signalling in progenitors is sufficient to activate STAT in both progenitors and the VM.

Minor concerns:

• The authors claim that "Draper-Src-Shark signaling acts in ISCs to recognize midgut damage and is required for full STAT activation in ISCs to promote their proliferation." However, Draper-Src-Shark signaling does not seem to completely block STAT activation in response to P.e. infection. The authors should review the manuscript carefully and revise claims that are not fully supported by the experimental data.

We thank the reviewer for pointing this out. We completely agree and did not mean to imply that all STAT activation after *P.e.* infection is due to Draper-Src-Shark signalling. We meant that together with cytokine signalling from the regenerative microenvironment, Draper-Src-Shark signalling was required for full STAT activation. As STAT activation is only partially decreased by depleting Draper-Src-Shark signalling, we believed that cytokine-Domeless signalling partly regulates STAT activation after *P.e.* infection.

Nevertheless, we have now found that Draper-Src-Shark signalling requires cytokine-Domeless signalling to activate STAT in progenitors and likely in the VM too. Further, we found that Draper signalling in progenitors does not activate STAT in the progenitors and VM by regulating cytokine *upd1-3* expression in the midgut epithelium. See Reviewer 1, point 5). We have modified the text and the Figure 5 model to make this clearer.

Reviewer #3 (Significance (Required)):

Although the role of Src42A in ISC proliferation upon bacterial infection has been previously reported, the mechanism elucidated in this study provides new insights into intestinal stem cell regulation and could contribute meaningfully to the stem cell field.

We thank the reviewer for the positive evaluation and for pointing out that our “study provides new insights into intestinal stem cell regulation and could contribute meaningfully to the stem cell field”.

Dear Dr Patel,

Thank you again for submitting your amended manuscript (EMBOJ-2025-121711R) to The EMBO Journal. Please accept my sincere apologies for the protraction with the reassessment due to delayed expert input at this time of the year and detailed discussion in the editorial team. Your revised study was sent back to the referees for their scientific reassessment, and we have received detailed from one of them, which I attach below. Please note that while the other referees were at this time not able to reassess your revised work, we have evaluated your response to their critique editorially and found the issues to be addressed satisfactorily. As you will see, the other referee states that the work has been substantially enhanced by the revisions and s/he is now in favour of publication.

Thus, we are pleased to inform you that your manuscript has been accepted in principle for publication in The EMBO Journal.

We now need you to take care of a number of minor issues related to formatting and data annotation as detailed in a separate information during the next days. There will also be requests by our production team for Source Data provision sent shortly.

As you might have seen on our web page, every paper at the EMBO Journal now includes a 'Synopsis', displayed on the html and freely accessible to all readers. The synopsis includes a 'model' figure as well as 2-5 one-short-sentence bullet points that summarize the article. I would appreciate if you could provide this figure and the bullet points.

Please submit a revised version of the manuscript using the link enclosed below.

Thank you again for giving us the chance to consider your manuscript for The EMBO Journal. I look forward to receiving your final revised version of the manuscript for swift acceptance and online publication.

Best regards,

Daniel Klimmeck

Daniel Klimmeck PhD
Senior Editor
The EMBO Journal

Please remember: Digital image enhancement is acceptable practice, as long as it accurately represents the original data and conforms to community standards. If a figure has been subjected to

significant electronic manipulation, this must be noted in the figure legend or in the 'Methods' section. The editors reserve the right to request original versions of figures and the original images that were used to assemble the figure.

Referee #1:

My concerns have been addressed. I have no further questions at this time.

***

Rev_Com_number: RC-2025-02969
New_manu_number: EMBOJ-2025-121711R1
Corr_author: Patel
Title: Damage recognition by intestinal stem cells via Draper promotes adult Drosophila midgut regeneration

Dear Dr Patel,

Further to below, I attach the mentioned list of formatting changes required for your final resubmission.

Please let us know any time should you have additional questions related.

Best regards,

Daniel Klimmeck

Daniel Klimmeck PhD
Senior Editor
The EMBO Journal

******

Formatting changes required for the revised version of the manuscript:

>> Author Contributions: Remove the author contributions information from the manuscript text. Note that CRediT has replaced the traditional author contributions section as of now because it offers a systematic machine-readable author contributions format that allows for more effective research assessment. and use the free text boxes beneath each contributing author's name to add specific details on the author's contribution.

More information is available in our guide to authors.
https://www.embopress.org/page/journal/14602075/authorguide

>> As you have seen on our webpage, every paper at the EMBO Journal includes a 'Synopsis', displayed on the html and freely accessible to all readers. The synopsis includes a 'model' figure as well as 2-5 one-short-sentence bullet points that summarize the article. I would appreciate if you could provide this figure and the bullet points.

>> Please correct the order and headings of the manuscript sections to Abstract / Introduction / Results / Discussion / Methods / Data Availability / Acknowledgements / Disclosure and Competing Interests Statement / References / Figure Legends / Expanded View Figure Legends

>> 'Methods and Methods' should be correct to 'Methods'.

>> Correct the title of the Disclosure and competing interest statement' section to 'Disclosure and Competing Interests Statement'.

>> Appendix file with ToC: add a cover page: Appendix for 'MS Title' and Table of Contents with each item listed (figures, tables) with its page number.

>> Reagents and Tools table: needs to be removed from manuscript and uploaded as a separate file.

Please compare also our Guide-to-Authors instructions:
https://www.embopress.org/page/journal/14602075/authorguide#structuredmethods

>> Please provide source data for the study as to the separate request e-mail. Source data should be uploaded as one (zipped) file per figure.

>> Please recheck references for the bioRxiv entry Mishra et al. (2025) and update the citation if in the meantime published as regular article.

>> Consider additional changes and comments from our production team as indicated below:

Figure Legends - Comments

- Please define the annotated p values ****/***/**/* as well as provide the exact p-values for the same in the legend of figure 1J, L, M, N; 2A-J; 4I, K; EV1 E, I, J; EV2 A, B, D, E, G, H; EV4 I, L; is appropriate.
- Please note that the yellow arrows are not defined in the legend of figure EV1 F-G'. This needs to be rectified.

Further information is available in our Guide For Authors: https://link.springer.com/journal/44318/submission-guidelines

The authors addressed the remaining editorial issues.

2nd Revision - Editorial Decision 22nd Jul 2026

Dear Parthive,

Thank you for submitting the final revised version of your manuscript. I have now evaluated your amended manuscript and concluded that the remaining minor concerns have been sufficiently addressed.

I am thus pleased to inform you that your manuscript has been accepted for publication in the EMBO Journal.

Your manuscript will be processed for publication by EMBO Press. It will be copy edited and you will receive page proofs prior to publication. Please note that you will be contacted by Springer Nature Author Services to complete licensing and payment information.

Please note that it is The EMBO Journal policy for the transcript of the editorial process (containing referee reports and your response letter) to be published as an online supplement to each paper. If you do NOT want this, you will need to inform the Editorial Office via email immediately. More information is available here: https://www.embopress.org/transparent-process#Review_Process

You may qualify for financial assistance for your publication charges - either via a Springer Nature fully open access agreement or an EMBO initiative. Check your eligibility: https://www.embopress.org/page/journal/14602075/authorguide#chargesguide

Should you be planning a Press Release on your article, please notify the production team via this form in order to coordinate publication and release dates: https://forms.cloud.microsoft/e/eH4A8JVP6g

If you have any questions, please do not hesitate to contact the Editorial Office. Thank you for your contribution to The EMBO Journal.

Thank you again for this contribution to The EMBO Journal and congratulations on a successful publication! Please consider us again in the future for your most exciting work.

Best regards,

Daniel

Daniel Klimmeck, PhD
Senior Editor
The EMBO Journal
EMBO
Postfach 1022-40
Meyerhofstrasse 1
D-69117 Heidelberg
contact@embojournal.org

*****
Please note that it is The EMBO Journal policy for the transcript of the editorial process (containing referee reports and your response letters) to be published as an online supplement to each paper. If you should prefer removal of any referee-only figures included in the point-by-point response(s), e.g. because they may still be used for future publication or because they have been reproduced from published work by others, please do let us know immediately via response email.
More information is available here: https://link.springer.com/partners/embo-press/editorial-policies#Peer%20review
*****

***
Rev_Com_number: RC-2025-02969
New_manu_number: EMBOJ-2025-121711R2
Corr_author: Patel
Title: Damage recognition by intestinal stem cells via Draper promotes adult Drosophila midgut regeneration

EMBO Press Author Checklist

Corresponding Author Name: Parthive H. patel	
Journal Submitted to: EMBO Journal	
Manuscript Number: EMBOJ-2025-121711R2	

USEFUL LINKS FOR COMPLETING THIS FORM
The EMBO Journal - Author Guidelines
EMBO Reports - Author Guidelines
Molecular Systems Biology - Author Guidelines
EMBO Molecular Medicine - Author Guidelines

Reporting Checklist for Life Science Articles (updated January
This checklist is adapted from Materials Design Analysis Reporting (MDAR) Checklist for Authors. MDAR establishes a minimum set of requirements in transparent reporting in the life sciences (see Statement of Task: 10.31222/osf.io/9sm4x). Please follow the journal's guidelines in preparing your
Please note that a copy of this checklist will be published alongside your article.

Abridged guidelines for figures
1. Data
The data shown in figures should satisfy the following conditions:
- the data were obtained and processed according to the field's best practice and are presented to reflect the results of the experiments in an accurate and unbiased manner.
- ideally, figure panels should include only measurements that are directly comparable to each other and obtained with the same assay.
- plots include clearly labeled error bars for independent experiments and sample sizes. Unless justified, error bars should not be shown for technical
- if n<5, the individual data points from each experiment should be plotted. Any statistical test employed should be justified.
- Source Data should be included to report the data underlying figures according to the guidelines set out in the authorship guidelines on Data

2. Captions
Each figure caption should contain the following information, for each panel where they are relevant:
- a specification of the experimental system investigated (eg cell line, species name).
- the assay(s) and method(s) used to carry out the reported observations and measurements.
- an explicit mention of the biological and chemical entity(ies) that are being measured.
- an explicit mention of the biological and chemical entity(ies) that are altered/varied/perturbed in a controlled manner.
- the exact sample size (n) for each experimental group/condition, given as a number, not a range;
- a description of the sample collection allowing the reader to understand whether the samples represent technical or biological replicates (including how many animals, litters, cultures, etc.).
- a statement of how many times the experiment shown was independently replicated in the laboratory.
- definitions of statistical methods and measures:
  - common tests, such as t-test (please specify whether paired vs. unpaired), simple $\chi 2$ tests, Wilcoxon and Mann-Whitney tests, can be unambiguously identified by name only, but more complex techniques should be described in the methods section;
  - are tests one-sided or two-sided?
  - are there adjustments for multiple comparisons?
  - exact statistical test results, e.g., P values = x but not P values < x;
  - definition of 'center values' as median or average;
  - definition of error bars as s.d. or s.e.m.

Please complete ALL of the questions below.
Select "Not Applicable" only when the requested information is not relevant for your study.

Materials

Newly Created Materials	Information included in the manuscript?	In which section is the information available? (Reagents and Tools Table, Materials and Methods, Figures, Data Availability Section)
New materials and reagents need to be available; do any restrictions apply?	Not Applicable	

Antibodies	Information included in the manuscript?	In which section is the information available? (Reagents and Tools Table, Materials and Methods, Figures, Data Availability Section)
For **antibodies** provide the following information:<br>- Commercial antibodies: RRID (if possible) or supplier name, catalogue number and or/clone number<br>- Non-commercial: RRID or citation	Yes	Reagents and Tools Table

DNA and RNA sequences	Information included in the manuscript?	In which section is the information available? (Reagents and Tools Table, Materials and Methods, Figures, Data Availability Section)
**Short novel DNA or RNA including primers, probes**: provide the sequences.	Not Applicable	

Cell materials	Information included in the manuscript?	In which section is the information available? (Reagents and Tools Table, Materials and Methods, Figures, Data Availability Section)
**Cell lines:** Provide species information, strain. Provide accession number in repository **OR** supplier name, catalog number, clone number, and/**OR** RRID.	Not Applicable	
**Primary cultures:** Provide species, strain, sex of origin, genetic modification status.	Not Applicable	
Report if the cell lines were recently **authenticated** (e.g., by STR profiling) and tested for mycoplasma contamination.	Not Applicable	

Experimental animals	Information included in the manuscript?	In which section is the information available? (Reagents and Tools Table, Materials and Methods, Figures, Data Availability Section)
**Laboratory animals or Model organisms:** Provide species, strain, sex, age, genetic modification status. Provide accession number in repository **OR** supplier name, catalog number, clone number, **OR** RRID.	Yes	Materials and Methods, Reagents and Tools Table
**Animal observed in or captured from the field:** Provide species, sex, and age where possible.	Not Applicable	
Please detail **housing and husbandry conditions**.	Yes	Materials and Methods

Plants and microbes	Information included in the manuscript?	In which section is the information available? (Reagents and Tools Table, Materials and Methods, Figures, Data Availability Section)
**Plants:** provide species and strain, ecotype and cultivar where relevant, unique accession number if available, and source (including location for collected wild specimens).	Not Applicable	
**Microbes:** provide species and strain, unique accession number if available, and source.	Yes	Material and Methods

Human research participants	Information included in the manuscript?	In which section is the information available? (Reagents and Tools Table, Materials and Methods, Figures, Data Availability Section)
If collected and within the bounds of privacy constraints report on age, sex and gender or ethnicity for all study participants.	Not Applicable	

Core facilities	Information included in the manuscript?	In which section is the information available? (Reagents and Tools Table, Materials and Methods, Figures, Data Availability Section)
If your work benefited from core facilities, was their service mentioned in the acknowledgments section?	Not Applicable	

Design

Study protocol	Information included in the manuscript?	In which section is the information available? (Reagents and Tools Table, Materials and Methods, Figures, Data Availability Section)
If study protocol has been **pre-registered, provide DOI in the manuscript**. For clinical trials, provide the trial registration number **OR** cite DOI.	Not Applicable	
Report the **clinical trial registration number** (at ClinicalTrials.gov or equivalent), where applicable.	Not Applicable	

Laboratory protocol	Information included in the manuscript?	In which section is the information available? (Reagents and Tools Table, Materials and Methods, Figures, Data Availability Section)
Provide DOI OR other citation details if **external detailed step-by-step protocols** are available.	Not Applicable	

Experimental study design and statistics	Information included in the manuscript?	In which section is the information available? (Reagents and Tools Table, Materials and Methods, Figures, Data Availability Section)
Include a statement about **sample size** estimate even if no statistical methods were used.	Yes	Materials and Methods
Were any steps taken to minimize the effects of subjective bias when allocating animals/samples to treatment (e.g. **randomization procedure**)? If yes, have they been described?	Yes	Materials and Methods
Include a statement about **blinding** even if no blinding was done.	Yes	Materials and Methods
Describe **inclusion/exclusion criteria** if samples or animals were excluded from the analysis. Were the criteria pre-established?<br><br>If sample or data points were omitted from analysis, report if this was due to attrition or intentional exclusion and provide justification.	Not Applicable	
For every figure, are **statistical tests** justified as appropriate? Do the data meet the assumptions of the tests (e.g., normal distribution)? Describe any methods used to assess it. Is there an estimate of variation within each group of data? Is the variance similar between the groups that are being statistically compared?	Yes	Materials and Methods

Sample definition and in-laboratory replication	Information included in the manuscript?	In which section is the information available? (Reagents and Tools Table, Materials and Methods, Figures, Data Availability Section)
In the figure legends: state number of times the experiment was **replicated** in laboratory.	Yes	Figure legends
In the figure legends: define whether data describe **technical or biological replicates**.	Yes	Figure legends

Ethics

Ethics	Information included in the manuscript?	In which section is the information available? (Reagents and Tools Table, Materials and Methods, Figures, Data Availability Section)
Studies involving **human participants**: State details of **authority granting ethics approval** (IRB or equivalent committee(s), provide reference number for approval.	Not Applicable	
Studies involving **human participants**: Include a statement confirming that **informed consent** was obtained from all subjects and that the experiments conformed to the principles set out in the WMA Declaration of Helsinki and the Department of Health and Human Services Belmont Report.	Not Applicable	
Studies involving **human participants: For** publication of **patient photos**, include a statement confirming that consent to publish was obtained.	Not Applicable	
Studies involving experimental **animals**: State details of **authority granting ethics approval** (IRB or equivalent committee(s), provide reference number for approval. Include a statement of compliance with ethical regulations.	Not Applicable	
Studies involving **specimen and field samples:** State if relevant **permits** obtained, provide details of authority approving study; if none were required, explain why.	Not Applicable	

Dual Use Research of Concern (DURC)	Information included in the manuscript?	In which section is the information available? (Reagents and Tools Table, Materials and Methods, Figures, Data Availability Section)
Could your study fall under dual use research restrictions? Please check biosecurity documents and list of **select agents and toxins** (CDC): https://www.selectagents.gov/sat/list.htm	Not Applicable	
If you used a select agent, is the security level of the lab appropriate and reported in the manuscript?	Not Applicable	
If a study is subject to dual use research of concern regulations, is the name of the **authority granting approval and reference number** for the regulatory approval provided in the manuscript?	Not Applicable	

Reporting

The MDAR framework recommends adoption of discipline-specific guidelines, established and endorsed through community initiatives. Journals have their own policy about requiring specific guidelines and recommendations to complement MDAR.

Adherence to community standards	Information included in the manuscript?	In which section is the information available? (Reagents and Tools Table, Materials and Methods, Figures, Data Availability Section)
State if relevant guidelines or checklists (e.g., **ICMJE, MIBBI, ARRIVE, PRISMA**) have been followed or provided.	Not Applicable	
For **tumor marker prognostic studies,** we recommend that you follow the **REMARK** reporting guidelines (see link list at top right). See author guidelines, under 'Reporting Guidelines'. Please confirm you have followed these guidelines.	Not Applicable	
For **phase II and III randomized controlled trials**, please refer to the **CONSORT** flow diagram (see link list at top right) and submit the CONSORT checklist (see link list at top right) with your submission. See author guidelines, under 'Reporting Guidelines'. Please confirm you have submitted this list.	Not Applicable	

Data Availability

Data availability	Information included in the manuscript?	In which section is the information available? (Reagents and Tools Table, Materials and Methods, Figures, Data Availability Section)
Have **primary datasets** been deposited according to the journal's guidelines (see 'Data Deposition' section) and the respective accession numbers provided in the Data Availability Section?	Not Applicable	
Were **human clinical and genomic datasets** deposited in a public access-controlled repository in accordance to ethical obligations to the patients and to the applicable consent agreement?	Not Applicable	
Are **computational models** that are central and integral to a study available without restrictions in a machine-readable form? Were the relevant accession numbers or links provided?	Not Applicable	
If publicly available data were reused, provide the respective **data citations in the reference list**.	Not Applicable	
